# Mesenchymal stem cells in preclinical cancer cytotherapy: a systematic review

**DOI:** 10.1186/s13287-018-1078-8

**Published:** 2018-12-07

**Authors:** Ioannis Christodoulou, Maria Goulielmaki, Marina Devetzi, Mihalis Panagiotidis, Georgios Koliakos, Vassilis Zoumpourlis

**Affiliations:** 1 Institute of Biological Research and Biotechnology, National Hellenic Research Foundation (NHRF), Konstantinou 48 Av., 116 35 Athens, Greece; 2 Northumbria University Newcastle upon Tyne, Newcastle upon Tyne, UK; 3 Aristotle University of Thessaloniki, Thessaloniki, Greece

**Keywords:** Adult mesenchymal stem cells, Umbilical cord matrix stem cells, Wharton’s jelly, Tumor microenvironment, Experimental cancer cytotherapy, Gene delivery vehicles

## Abstract

Mesenchymal stem cells (MSC) comprise a heterogeneous population of rapidly proliferating cells that can be isolated from adult (e.g., bone marrow, adipose tissue) as well as fetal (e.g., umbilical cord) tissues (termed bone marrow (BM)-, adipose tissue (AT)-, and umbilical cord (UC)-MSC, respectively) and are capable of differentiation into a wide range of non-hematopoietic cell types. An additional, unique attribute of MSC is their ability to home to tumor sites and to interact with the local supportive microenvironment which rapidly conceptualized into MSC-based experimental cancer cytotherapy at the turn of the century. Towards this purpose, both naïve (unmodified) and genetically modified MSC (GM-MSC; used as delivery vehicles for the controlled expression and release of antitumorigenic molecules) have been employed using well-established in vitro and in vivo cancer models, albeit with variable success. The first approach is hampered by contradictory findings regarding the effects of naïve MSC of different origins on tumor growth and metastasis, largely attributed to inherent biological heterogeneity of MSC as well as experimental discrepancies. In the second case, although the anti-cancer effect of GM-MSC is markedly improved over that of naïve cells, it is yet apparent that some protocols are more efficient against some types of cancer than others. Regardless, in order to maximize therapeutic consistency and efficacy, a deeper understanding of the complex interaction between MSC and the tumor microenvironment is required, as well as examination of the role of key experimental parameters in shaping the final cytotherapy outcome. This systematic review represents, to the best of our knowledge, the first thorough evaluation of the impact of experimental anti-cancer therapies based on MSC of human origin (with special focus on human BM-/AT-/UC-MSC). Importantly, we dissect the commonalities and differences as well as address the shortcomings of work accumulated over the last two decades and discuss how this information can serve as a guide map for optimal experimental design implementation ultimately aiding the effective transition into clinical trials.

**Electronic supplementary material:**

The online version of this article (10.1186/s13287-018-1078-8) contains supplementary material, which is available to authorized users.

## Background

In the turn of the millennium after over four decades of intensive research in the genetics of cancer, an important paradigm shift has occurred, in which instrumental for the development of the disease is not only the link between cumulative instability of key genes and the rise of aberrant cell cycle control, but also the orchestration of a heterogeneous cell-based stromal network supportive of tumor invasion and metastasis. This switch in focus has led to the elucidation of the composition of this microenvironmental niche and of the interaction dynamics of its multiple cellular and extracellular components. Concomitant have been the emergence of the epithelial-to-mesenchymal transition (EMT) process as a key cellular mechanism in tumorigenesis, as well as the important discovery that stem cells of mesenchymal origin (mesenchymal stem cells (MSC)) can home towards tumor sites. The latter has formed the basis of an exciting new anti-cancer strategy, namely cancer cytotherapy, in which allogeneic MSC can be used to specifically target and deregulate the local tumor microenvironment, as an answer to modern oncology’s desperate need for a promising alternative to the current gold-standard therapy of combined chemotherapy (Additional file 1: Figure S1).

Towards this end, various MSC populations have been recruited in different experimental setups in dozens of in vitro and in vivo preclinical studies. Unfortunately, until recently, MSC-based cancer cytotherapy has to a large extent failed to deliver its initial promise of a high-end translational oncology option. In their vast majority, the 500 or more clinical trials employing the use of MSC take advantage of the immunomodulatory, anti-inflammatory, and tissue regenerative properties of these cells for treating, for example, autoimmune diseases, graft rejection and osteochondral degeneration, without, until very recently, any significant data on the anti-cancer efficacy of MSC in humans. The problem of translating experimental MSC-based cancer cytotherapy work into successful human clinical trials is multi-faceted. In the case where MSC are engineered to overexpress molecules with anti-cancer properties, tumor suppression is achieved with relatively high efficiency and reproducibility in the majority of experimental protocols applied. Nevertheless, genetic engineering of cells not only adds extra costly and time-consuming manipulation steps in vitro, but also raises safety concerns since it usually relies on the use of viral vectors that introduce instability and chances of mutation at the genome integration sites of the host. On the other hand, the use of naïve, unmodified stem cells, although more straightforward, is hampered by contradictory findings, largely driven by the immense biological variability inherent to cell-based products and therapies (compared to traditional pharmaceuticals), as well as by multi-parameter variability relating to the methods, processes and manipulation steps mediating transformation of lab findings to clinical-grade products.

With a mortality rate of nearly 60% for the over 14 million people diagnosed with cancer yearly worldwide, it is imperative that any novel therapeutic regime should be optimized to its full potential. Obviously, experimental cancer cytotherapy is at a nodal point, where the critical mass of information accumulated by the series of studies performed over the last two decades needs to be thoroughly revised, carefully analyzed, and properly evaluated in the context of immunobiology of the tumor microenvironment. With the first-in-human clinical trials assessing genetically modified MSC in gastrointestinal and lung cancer settings only very recently and more to follow, this task has become an essential one more than ever before [1]. More specifically, in order for the field to move forward, any new experimental attempt should be built upon (a) a clear understanding of the interactions between components of the local tumor environment during its progression from benign stromal tissue to a niche tuned to support growth, invasion, and metastasis of malignant cells and (b) careful experimental design that minimizes unnecessary duplication of work as well as the emergence of technical heterogeneity.

With all the above in mind, we present here a systematic review of the advances in the MSC-based cancer cytotherapy field from its inception onwards, including the state-of-the art of the biology and relationship between the effectors (MSC populations) and targets (tumor niche components) implicated (discussed in the section “MSC and the tumor microenvironment—attributes and inter-relationship in the context of cancer cytotherapy”). Amalgamated in this review is a meta-analysis of the related bibliography, which was carefully designed and implemented in such a way to allow the extraction and decoding of the overwhelming number of experimental data presented therein and, hence enable the identification of commonalities and differences between studies, the dissection of emerging trends and patterns in reported findings, and possibly highlight any significant omissions or weaknesses in experimental design (presented in the “Methodology of the meta-analysis” and “Overview and discussion of the meta-analysis’ main findings” sections). Finally, in the concluding part (“Conclusions” section) of this review, an effort is made to both present the main findings as concise summary points in the context of state-of-the-art knowledge, which potentially can serve as a guide map, aiding the adoption of optimal conditions for the development of more efficient, translational cytotherapy protocols with extended clinical relevance.

## MSC and the tumor microenvironment—attributes and inter-relationship in the context of cancer cytotherapy

### The composition and dynamics of the tumor stromal microenvironment and the role of EMT in tumor development

Cells within the tumor parenchyma are not self-sustaining entities, but develop in a symbiotic manner by interacting via paracrine and juxtacrine signaling with the surrounding stroma. The stromal microenvironment is the non-neoplastic compartment of tumors and comprises a dynamic and highly heterogeneous network of tumor vasculature (including pericytes), infiltrating inflammatory and immune cells, extracellular matrix (ECM), (myo) fibroblastic cells (also known as tumor-associated fibroblasts (TAFs)), mesenchymal stromal/stem cells, and sometimes adipocytes [2], as well as reactive stroma [3]. Most if not all solid tumors have some degree of tumor stroma, and the presence of reactive stroma is often an indicator of poor prognosis [4]. Communication between cellular components of the tumor microenvironment is achieved through cytokines, chemokines, growth factors, and inflammatory and matrix remodeling enzymes that contribute to the malignant properties of the cells.

Among the immune cells that infiltrate the tumor stroma and are present within the tumor microenvironment, selective T and B lymphocyte populations favor or suppress tumor growth and their detection has been associated with subsequent clinical outcomes [5–7]. Tumor-associated macrophages (TAM) and neutrophils (TAN) are usually pro-tumorigenic. TAMs participate actively in metastasis, being thus implicated in poor prognosis [8], while TANs enhance angiogenesis and immune suppression [9, 10].

Perivascular stromal cells, known as pericytes, are another essential component of the tumor stroma that secures vasculature maintenance by providing structural support to blood vessels [11]. Although pericytes are present within normal blood vessels, tumor vessels are characterized by excess pericyte coverage that contributes to their abnormal physiology.

Fibroblastic stromal cells within the tumor stroma, known as TAFs, have been linked to several processes that promote cancer metastasis and growth, including angiogenesis [12], EMT [13], and progressive genetic instability [14, 15]. TAFs exert their tumor-promoting actions through secretion of growth factors, cytokines, chemokines, structural protein components, and metabolites that act upon tumor cells [2]. Additionally, fibroblastic stromal cells can deregulate antitumor immune responses, as exemplified by experiments demonstrating that allogeneic murine tumor cells, when co-injected with fibroblastic stromal cells, can engraft across immunologic barriers [16]. Together, these studies suggest that tissue-specific fibroblasts are influential players in the progression of metastatic cancer and, at first glance, appear to benefit tumor growth and decrease overall patient survival. However, there is mounting experimental evidence that healthy tumor microenvironments suppress tumor growth, and it is only after acquisition of tumor-like genetic lesions that fibroblasts appear to promote tumor progression [14, 17, 18].

ECM is another key regulator of tumor development and progression by providing both a structural scaffold for the tumor stroma and also active soluble factors including growth and angiogenic factors, cytokines, and chemokines that regulate tumor behavior. During tumor development, the ECM is usually disorganized and collagen deposition as well as cross-linking with other matrix proteins such as elastins, laminins, or fibronectin has been associated with cancer invasion and metastasis [19]. Among the ECM-secreted growth factors, TGF-β is known to promote EMT in cancer cells, thus increasing local and distant invasive potential [20].

ΕΜΤ is the process by which epithelial cells reduce the expression or function of proteins that promote cell-cell and cell-basement-membrane adhesion and thus obtain a mesenchymal-like phenotype. Except for the regulation of metastatic properties, the EMT has been also associated with acquired drug resistance [21]. Furthermore, high expression of EMT-induced markers (vimentin, α-smooth muscle actin (a-SMA), N-cadherin, cadherin 11, SpArC, laminin and fascin) with simultaneous low expression of E-cadherin has been associated with poor prognosis in patients with breast cancer [22].

### Principal common characteristics of isolated human MSC populations used in cytotherapy protocols

Stem cells possess a unique capacity of self-renewal, differentiation into multiple cell types and in vivo tissue repopulation [23]. These functions are triggered by signals that impel a stem cell to undergo either symmetric or asymmetric divisions [24]. Based on their ability to give rise to one or more different cell lineages, stem cells are characterized as totipotent (able to give rise to all cells constituting the developing embryo), pluripotent (e.g., isolated embryonic stem cells (ESC) that can differentiate into cells of all three germ layers), multipotent (capacity for differentiation towards most cell lineages), and unipotent (mono-specific differentiation). Mesenchymal stem cells (MSC) are multipotent stem cells that upon stimulation give rise to most body cell types including those of muscle, bone, fat, and cartilage lineages [25]. MSC were originally isolated from bone marrow (BM) aspirates; nevertheless, they can be isolated from many types of adult and fetal tissues using similar methodologies [26]. Adipose tissue (AT) provides a particularly abundant and accessible source, although many other adult tissue sites can also be utilized including kidney, skin, and the parathyroid gland [27]. In another study, it has also been suggested that a small population of MSC circulates within the peripheral blood and is highly mobilized during hypoxia [28]. MSC or MSC-like cells have been isolated from fetal tissues as well, including skin, cord blood (-CB), umbilical cord (-UC), and placenta [29]. Regardless of their origin, these MSC share similar defining characteristics including plastic adherence, cell surface marker expression (negative for hematopoietic markers CD34, CD35/positive for core markers CD29, CD44, CD73, CD90, CD105), and at least a tri-lineage differentiation potential (towards fat, bone, cartilage) under certain conditions [26]. The native functions are thought to include wound healing and support of hematopoiesis. More interestingly, when engrafted at sites of injury, MSC differentiate into connective tissue elements, support vasculogenesis, and secrete cytokines and growth factors that facilitate healing and tissue regeneration. In addition, due to their complex immunomodulatory properties, MSC can counteract inflammation, suppress host immune responses, and prevent fibrosis [30]. As a consequence of their diverse properties, MSC have been extensively utilized in the last decade in therapeutic applications such as tissue repair and regeneration, as well as autoimmune disease [31]. For example, the immunosuppressive effects of MSC have been used for therapy of graft-vs.-host disease (first MSC biologic drug).

Another common feature of MSC is that they are able to localize to sites of hematopoiesis, inflammation, or injury as well as to solid tumors, by a mechanism characterized as homing. Tumor tropism distinguishes MSC from other mesenchymal cells, such as differentiated fibroblasts [32]. This homing ability of MSC to injured sites and their interaction with the local microenvironment has encouraged investigation into the possibility of using these cells, either unmodified or as gene or even drug delivery vehicles for targeted cytotherapy, most notably cancer treatment [33, 34]. Interestingly, it has also been shown that co-injection of MSC promotes growth of various tumors in vivo, possibly due to the immunosuppressive effects that help cancer cells escape immunosurveillance [16, 35].

Tumor tropism of MSC and the dynamic interaction between MSC and the tumor microenvironment, due to their complexity as well as their significance for cancer therapy, require a deeper understanding and merit further investigation and hence are discussed in more detail in the following sections of this review.

### The tropism of MSC homing to tumors as the basis of cancer cytotherapy

**Fig. 1:**
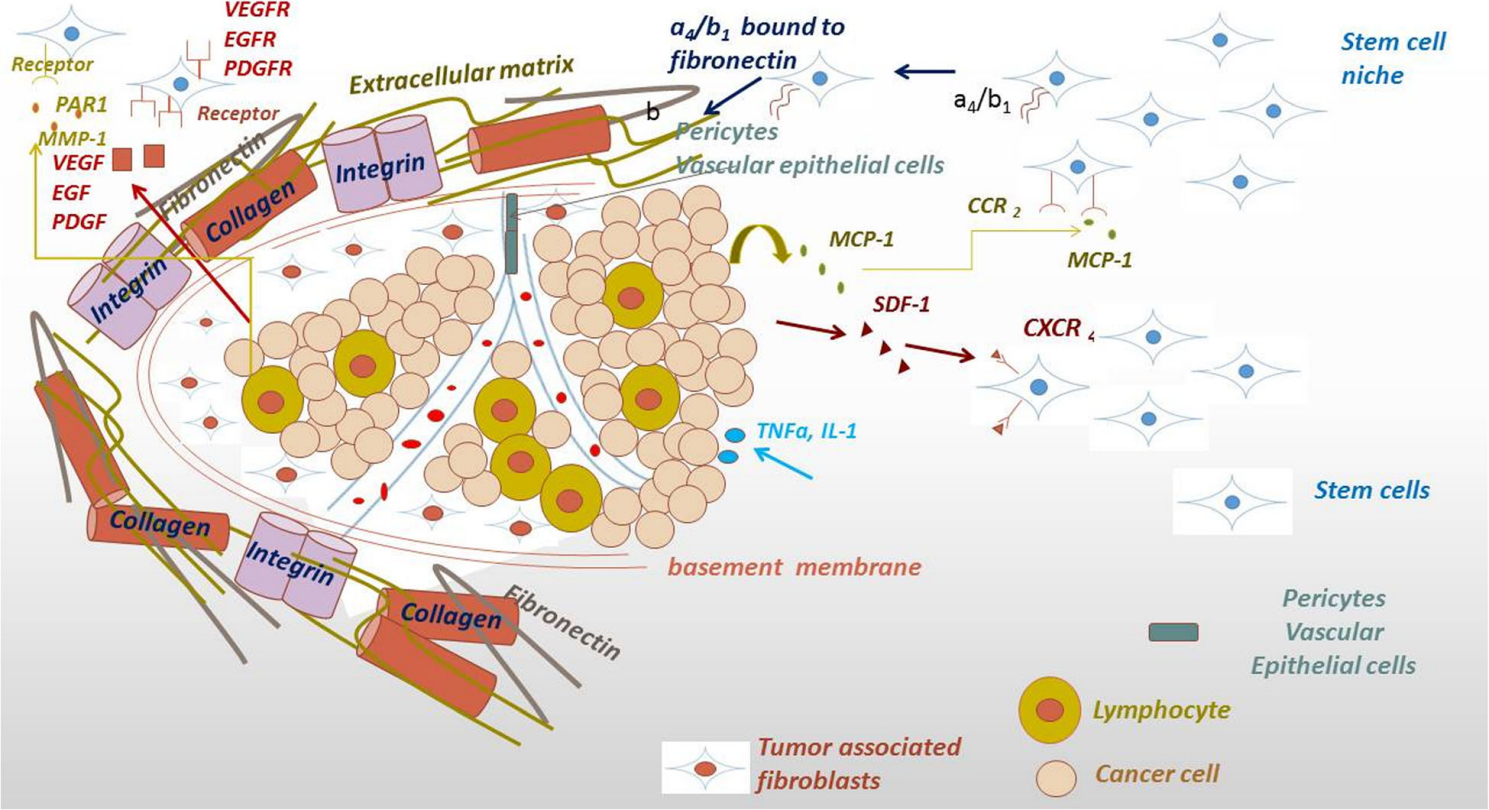
Mechanisms of MSC homing to tumor sites. Binding of monocyte chemotactic protein-1 (MCP-1 or CCL2), secreted by breast cancer cells or of stromal cell-derived factor 1 (SDF-1) secreted by breast, colon, and prostate cancer cells, on their receptors expressed on MSC surface can modulate the tropism of MSC to tumor sites. Matrix metalloproteinase 1 (MMP-1), localized in the extracellular matrix (ECM), stimulates MSC homing through cleavage and subsequent activation of the G-protein protease-activated receptor (PAR)-1. In correspondence with the homing process of MSC to sites of injury, the interaction between integrin α4/β1 on MSC and its binding site on fibronectin of the ECM plays a major role in the transmigration of MSC into the extracellular matrix. Finally, MSC recruitment can also be achieved through interaction of VEGF, secreted by cancer cells, with its receptor on MSC. After incorporating in tumor site, MSC in turn secrete various pro-angiogenic factors, such as VEGF, fibroblast-derived growth factor, PDGF, and SDF-1 that facilitate angiogenesis

Homing is the process by which cells migrate to, and engraft in, the tissue in which they can exert local functional effects. It is well established that MSC are recruited to sites of injury to support tissue repair, stem cell homeostasis, and immunomodulation. Tumors can be considered as chronic wounds and, thus, attract MSC in similar ways as injured tissues [36]. The homing ability of MSC towards tumors has been verified and studied in animal models in a variety of experimental settings, and it has been shown that MSC are active regulators of tumor progression [37, 38]. Homing and migration of MSC to tumor sites has been proved to be mediated by monocyte chemotactic protein-1 (MCP-1 or CCL2) secreted by primary breast cancers [39], or stromal cell-derived factor 1 (SDF-1), a small chemotactic cytokine that activates leukocytes and is often induced by proinflammatory stimuli such as TNF-α or IL-1 [40] in response to prostate, colorectal, and breast cancer in vitro [41]. On the other hand, in cases of malignant gliomas, MSC recruitment is achieved through interaction with a large array of angiogenesis-related cytokines including IL-8, TGF-β, and VEGF secreted by cancer cells [42]. Moreover, a component of the extracellular matrix, matrix metalloproteinase 1 (MMP-1) stimulates MSC homing through cleavage and subsequent activation of the G-protein protease-activated receptor (PAR)-1 [43] (Fig. 1).

It is believed that the mechanisms orchestrating MSC homing to sites of injury, and consequently to tumors, resemble the migration of leukocytes towards sites of injury and inflammation, which is well studied. It has been demonstrated that bone marrow stroma-derived MSC (BM-MSC) express high levels of adhesion molecules, including integrins β1 and a4, which can mediate the engraftment of MSC to the bone marrow [44, 45]. The α4/β1-VCAM-1 interaction has a central role in MSC communication with endothelial cells [46]. In the same context, the interaction between a4/β1 on MSC and its binding site on fibronectin of the ECM has been reported to play a major role in transmigration of MSC into the extracellular matrix [47] (Fig. 1). Other mechanisms contributing to the homing ability of MSC include P-selectin, MMP-2 secretion, and a number of cytokines [46].

Once in the tumor, attracted MSC interact with the tumor microenvironment leading to its remodeling and promoting cancer progression. It has been reported that MSC that home to tumor sites are transformed into TAFs [2] and promote tumor angiogenesis and invasive properties [48]. MSC interact with tumor cells in myriads of ways and modulate their behavior. Interactions between tumor cells and tumor-associated MSC are highly plastic and bidirectional. The activity of MSC within the tumor stroma includes enhanced secretion of TGF-β that contributes to the EMT process and immune-suppressive activities. Moreover, they release VEGF, which contributes to neovascularization within the tumor microenvironment and they also produce SDF-1 to support tumor cell growth and survival [2]. MSC also secrete CC-chemokine ligand 5 (CCL5), also known as RANTES, which interacts with specific cytokine receptors such as CCR1, CCR3, or CCR5. CCL5 paracrine signaling was found to promote the migratory, invasive, and metastatic properties of breast cancer cells [49].

Another way of communication between MSC and cancer cells includes the exchange of microparticles, like exosomes. MSC-derived exosomes can modulate the function of tumor cells through induction of MMP-2 and ecto-5′-nucleotidase activity, thus enhancing the heterogeneity within the tumor microenvironment [50], while they also enclose tumor supportive micro RNAs, which enhance tumor growth [51]. Simultaneously, cancer cells secrete exosomes that stimulate BM-MSC to differentiate into pro-angiogenic myofibroblasts with tumor-promoting properties [52]. Nevertheless, in many cases, MSC have been shown to exhibit tumoricidal behavior within tumors, as it will be discussed below.

The tumor microenvironment is a highly dynamic, plastic, and heterogeneous welter of cells and cellular factors that provide support and protection to the developing tumor and thus its therapeutic targeting constitutes a popular anti-cancer approach [13, 53]. The ability of MSC to home to and interact with tumors is being exploited for the effective targeting of the tumor microenvironment [54, 55].

### Divergent outcomes of MSC-based strategies for cancer cytotherapy

The unique homing property of MSC has been exploited in cancer cytotherapy protocols using various MSC populations (mainly of adult origin), either naïve or engineered to carry genes with antitumorigenic properties. But whilst genetically modified adult MSC have shown good efficacy in ex vivo and animal cancer models, naïve cells, on the contrary, have produced largely conflicting results by either promoting or suppressing tumor development. For a long period of time, mainly between 2000 and 2006, the research involving MSC-based cancer cytotherapy was limited only in the use of BM-MSC, with, in most cases, lack of encouraging results [56–58]. On the contrary, though, in an in vivo study by Khakoo et al., intravenously administered BM-MSC homed to tumorigenic regions and dynamically suppressed tumor growth in a mouse model of Kaposi’s sarcoma [59]. Since then, an increasing number of studies have come up with rather contradictory results regarding the use of MSC for experimental cancer treatment. Such conflicting (pro- vs. antitumorigenic) results have been recorded both in vitro and in vivo, for various cancer types in some cases even for the same cancer cell line. In support of these observations, the use of BM-MSC against colon cancer cell lines has either led to promotion of the tumorigenic properties of these MSC [60] positively affecting proliferation of cancer cells, or, on the contrary, was cytotoxic for the latter [57]. Furthermore, an onco-suppressive effect could not be established in vivo either, since human fetal or adult-derived BM-MSC were found to promote tumor growth, following their subcutaneous co-injection with SW480 colon cancer cells, in BALB/c-nu/nu mice [61]. Bone marrow-derived MSC are, though, not the only stem cells that show this erratic behavior. Due to the relatively greater ease and efficiency of isolation, adipose tissue-derived MSC (AT-MSC) have also been recruited for the development of experimental cancer cytotherapy protocols. Similarly to BM-MSC, AT-MSC have been shown both to promote and inhibit the survival of brain [62–64], breast [65], and prostate [66, 67] cancers in vitro and in vivo. The number of reports implicating umbilical cord matrix-derived MSC (UC-MSC) in conflicting cytotherapy outcomes is, so far, scarce and limited to in vitro experimentation. For example, a study by Li et al. [68], reporting the promotion of proliferation and metastasis of MDA-MB-231 and MCF-7 breast cancer cells in vitro, is outweighed by a series of studies showcasing the opposite effect on the two cell lines in vitro and in vivo [69–73].

Besides conflicting results observed within specific MSC (effector) and tumor (target) combinations, contradictory findings have also been obtained with regard to the effect of different MSC populations on the same tumor target, strongly suggesting a close association between the antitumorigenic properties of the former and their developmental origin. With respect to breast cancer, for example, a great number of studies (mainly in vitro) have quite convincingly showed a robust tumor-promoting effect of both BM-MSC [49, 56, 58, 74–78] and AT-MSC [62, 65, 79, 80] on at least two different breast cancer cell lines. On the other hand, an equally large group of studies using UC-MSC clearly demonstrate the opposite (onco-suppressive effect) on the same cell lines both in vitro and in vivo, as stated previously [69–73].

Finally, apart from the cases where the dubious efficacy of certain MSC against some tumors arises from contrasting reports in the literature, there are also cases where questions are raised over the therapeutic value of those naïve MSC that have been shown to possess strong onco-suppressive characteristics, albeit by yet unconfirmed reports. Such examples are the cases of AT-MSC vs. pancreatic cancer [66], BM-MSC vs. non-Hodgkin’s lymphoma [81] and UC-MSC vs. prostate [82] and bladder tumors [83].

Clearly, more focused work will lead to data accumulation, analysis of which will help in identifying trends and will fill in knowledge gaps regarding effectiveness of experimental cancer cytotherapy, at the same time shedding more light on the underlying biological mechanisms.

In the following sections, we discuss some of these mechanisms that may attribute to the complexity and adversity of experimental cancer cytotherapy outcomes and next present our meta-analysis strategy for decoding all available data from the field at its current state of maturity.

### Possible mechanisms mediating the bimodal effects of exogenous MSC on tumors

Once MSC migrate to the sites of tumor development, they interact with components of the neoplastic parenchyma as well as with supportive stroma. The exact dynamics of this crosstalk is dependent on the developmental stage and origin of the MSC as well as the type and site of tumor targeted. Nevertheless, the final outcome, tumor support or suppression, is most likely determined by the resulting tilt in balance between the respective mediating mechanisms (reviewed in [84]). In the first case, instrumental processes for MSC are the promotion of angiogenesis and contribution to the fibrovascular stromal network, mitigation of immune reactions, and stimulation of EMT and metastatic processes, while in the case where MSC exert a suppressive effect on the developing tumor, mechanisms such as cancer cell control, apoptosis induction, and regulation of Wnt and AKT signaling are involved and dominate.

#### Support of tumor vasculature and fibrovascular network

Α significant amount of in vivo experimental data, including labeled MSC tracking, support the contribution of MSC to the tumor vascular and fibrovascular network, either directly by differentiating into pericytes, fibroblasts, and myofibrobasts that transform into TAFs [35, 85] or indirectly through secretion of specific growth factors [86]. A MSC-like population expressing the characteristic surface markers CD10, CD13, and CD90 has been identified within pericytes isolated from the stromal-vascular compartment [87, 88].

Once in the tumor microenvironment, MSC acquire expression of TAF antigens, such as a-smooth muscle actin (a-SMA), fibroblast-specific protein, vimentin, and SDF-1 in vivo and in vitro following co-culture with tumor cells or using tumor-conditioned media [35, 89]. In accordance with this data, human BM-MSC have been found to promote angiogenesis and tumor blood vessel reorganization in a murine mammary adenocarcinoma model, with increased a-SMA expression, when hMSC were injected in the tumor periphery or intravenously [90]. In another orthotopic mouse model of colon cancer, co-injected MSC were incorporated into the tumor stroma and expressed a-SMA, PDGFRb, desmin, FSP, and FAP as TAF markers [91]. However, when hAT-MSC were co-injected with U87MG and H460 brain tumor cells in BALB/c nude mice, no vascular support was observed [63].

MSC also secrete various pro-angiogenic factors, such as VEGF, fibroblast-derived growth factor, PDGF, and stromal-derived factor-1 (SDF-1), that facilitate angiogenesis through promotion of endothelial and smooth muscle migration and proliferation towards the tumor site [92]. MSC-expressing VEGF caused increased microvessel density in pancreatic xenografts [93] and enhanced neovascularization in syngeneic mouse models of melanoma and lung tumors [94]. However, VEGF is not the basic factor that promotes angiogenesis and other pro-angiogenic cytokines must be involved in tumor vasculature expansion by MSC, as recombinant VEGF did not have the same effect on vessel growth as did the MSC-conditioned media [92]. VEGF, IL-8, angiogenin, and CCL2 were significantly enhanced by the concomitant presence of MSC and lymphoma cells in C.B-17 severe combined immunodeficiency (SCID) mice, contributing to the migration of endothelial cells in transwell assays. However, when MSC were directly co-cultured with endothelial cells, a significant induction of endothelial cell apoptosis was recorded [81]. Other MSC-secreted growth factors implicated on tumor vasculature facilitation include hepatocyte growth factor, cyclooxygenase, IGF-1, PDGF-a, and transforming growth factor-a1 [93]. Finally, it has been proposed that gastric cancer exosomes can trigger differentiation of UC-MSC to carcinoma-associated fibroblasts and thus enhance the tumor fibrovascular network, an effect that can be eliminated through blockade of the TGF-β pathway [95].

On the other hand, a number of studies support the involvement of MSC in suppression of the tumor angiogenic network. MSC were found to migrate towards and inhibit growth of endothelial cell-derived capillaries in vitro through production of reactive oxygen species. The growth-suppressing effect of MSC was also observed in vivo, where established melanoma tumors injected with MSC exhibited lower vascular density [96].

#### Immunomodulatory effects on tumors

MSC are thought to promote tumorigenesis through their immunomodulatory behavior, which is characterized mainly by immunosuppressive effects that can be beneficial for cancer cells to escape the immune system surveillance [97]. MSC act directly on immune cells, including B and T lymphocytes, dendritic cells, and natural killer cells [97–99]. MSC can suppress T cell activity by either inhibiting their proliferation or, in case of activated T lymphocytes, by leading them to apoptosis [100, 101]. Inhibition of T cell proliferation has been found enhanced by different mechanisms, like interferon (IFN)-gamma-mediated upregulation of an inhibitory cell surface marker, B7-H1 [102], or by Stro-1 expression [103]. In addition, Toll-like receptor (TLR) signaling has also been shown to contribute to the immunomodulatory properties of MSC, as expression of certain receptors can lead MSC to switch from a predominately immune-suppressive to a proinflammatory phenotype [104]. On the other hand, Puissant et al. showed that both AT-MSC and BM-MSC can inhibit lymphocytes only when they are in close contact [105], while in another study, UC-MSC (isolated from Wharton’s Jelly (WJ)-MSC) were able to suppress proliferation of peripheral blood lymphocytes [106].

The in vivo immunomodulatory effects of MSC are still poorly studied within tumors. In a study by Djouad et al., the reported immunosuppressive action of MSC led to a higher incidence of melanoma formation in an allogeneic mouse model [16]. Based on the immunosuppressive properties of MSC, these cells have been proposed to suppress the graft-vs.-leukemia effect and the graft-vs.-host response [107, 108].

#### Pro-metastatic effects and stimulation of EMT

The contribution of MSC to the establishment of distant metastases is rather controversial, ranging from promotion to suppression in a number of studies. For example, intravenous injection of MSCs derived from umbilical cord blood or adipose tissue reduced the formation of lung metastases in mice with established mammary tumors [109]. In contrast, mice that received subcutaneous co-injections of human breast cancer cells with human BM-MSC displayed a marked increase in the numbers of micro- and macroscopic lung metastases. The pro-metastatic effect of MSC was found mediated through paracrine action, by the secretion of chemokine CCL5 from MSC in two out of four cell lines tested, as the metastatic potential was abolished when CCL5 expression was eliminated [49]. In a 3D model of hepatocellular carcinoma, co-culture with UC-MSC led to increased secretion of MMP2, which in turn enhanced the invasive ability of cancer cells [110]. Additionally, hBM-MSC secrete IL-17B, which may act through IL-17BR—a prognostic indicator of breast cancer progression and metastasis—to stimulate metastasis. This hypothesis was tested in a humanized model of breast cancer metastasis to bone, where co-injection of cancer cells with BM-MSC increased the frequency of metastases with increased expression of IL-17BR [75].

MSC may also modulate EMT, a developmental process that is subverted by tumor cells, resulting in a more invasive phenotype. Co-culture of breast cancer cells with MSC resulted in upregulation of EMT-specific markers (N-cadherin, vimentin, Twist, and Snail) and a decrease in E-cadherin [111]. Another mechanism of MSC-mediated promotion of metastasis includes the formation of early metastasis through vasculogenesis or growth factor secretion. Accordingly, MSC facilitated the entry of breast cancer cells into the bone marrow through Tac-1 regulation of SDF-a1 and C-X-C chemokine receptor type 4 (CXCR4) [112]. In support of this observation, Zhang et al. showed that MSC from the bone marrow can promote pulmonary metastasis of osteosarcoma tumors in mice, through a mechanism involving the CXCR4/VEGF axis [113].

#### Regulation of signaling pathways

In many cases, the tumoricidal action of MSC on tumors has been associated with suppressive effects on signaling pathways crucial for cancer progression, proliferation, and survival, mainly involving the PI3K/AKT and Wnt pathways. Inhibition of AKT was reported in a Kaposi’s sarcoma model, where intravenously injected MSC migrated to tumors and effectively inhibited tumor proliferation [59]. MSC can also suppress the WNT/β-catenin pathway through induced expression and secretion of DKK-1 in human carcinoma cell lines. Interestingly, when DKK-1 was inhibited, tumor cell proliferation was restored [114, 115]. The ERK/MAPK signaling pathway has also been implicated in tumor promotion by MSC, since drug-mediated inhibition of ERK in breast cancer cells cultured in MSC-CM resulted in decreased proliferation of tumor cells [68].

Many studies have concentrated on the anti-cancer functions of neonatal MSC, highlighting the primitive characteristics that render them non-tumorigenic. Among these, WJ-MSC show modest expression of pluripotency genes and high levels of several tumor suppressor genes, while they secrete a variety of growth factors and cytokines that inhibit tumor growth [70, 116, 117].

#### Regulation of cell cycle and apoptosis

Studies investigating the effects of MSC on tumors have shown that they either promote or inhibit apoptosis, leading to tumor attenuation or progression, respectively. Interactions between MSC and cancer cells in the bone marrow have been shown to promote survival of acute myelogenous leukemia through upregulation of anti-apoptotic bcl-2 with reduced rates of apoptosis in response to cytotoxic chemotherapy [118]. Furthermore, culture of colorectal cancer cells in the conditioned medium (CM) of MSC downregulated the expression of the apoptosis-related proteins Bax and p53 and upregulated the anti-apoptotic protein Bcl-2, leading to inhibition of apoptosis, while under the same conditions cancer cell cycle was promoted with an increased percentage of cells found in the S phase [60]. Except for promoting anti-apoptotic events, MSC have been reported to protect osteosarcoma cells from drug-induced apoptosis, through activation of the STAT3 pathway [119].

On the other hand, Lu et al. demonstrated that MSC had an inhibitory effect on mouse tumor cells and ascitogenic hepatoma cells in a cell-dependent manner involving Caspase 3, an apoptotic protein, and p21, a negative regulator of cell cycle, implying that MSC exert tumor inhibitory effects in the absence of host immunosuppression, by inducing G0/G1 phase arrest and apoptosis of cancer cells [120]. G1 phase arrest was also observed when leukemia cells were cultivated with MSC in vitro [34]. Similar results were obtained after intratumoral administration of MSC into melanoma-bearing mice, where interaction of MSC with the neocapillary network in the tumors caused cytotoxicity and apoptosis of the tumor-associated endothelial cells [96].

### MSC-based cancer cytotherapy—current challenges and gaps of knowledge

Unquestionably over the last quarter of the century, the explosion of research in molecular cell biology and immunobiology of stem cells and cancer have shed light on the composition and establishment of the tumor microenvironment, on mechanisms governing MSC homing to tumor sites and interaction with components therein, as well as on signaling cascades and molecular events mediating the pro- or antitumorigenic effects. In its majority, this bulk of knowledge has stemmed from applied work using MSC in variety of in vitro/in vivo tumor models. However, the complexity of the field and the heterogeneity and diversity of the outcomes have inevitably led to new elementary questions that remain to be resolved. Some of these are: Have research work efforts so far been correctly prioritized or have they been prone to partiality and duplication? What are the crucial areas that we may need to focus on both experimentally and conceptually in order to maximize clinical relevance/impact? To what extent is the discrepancy in results attributed to technical rather than biological heterogeneity? What are the mechanisms mediating anti-cancer efficacy in different MSC populations? Can we improve consistency and efficiency of primary outcome by optimization of experimental parameters? Is there an MSC type/source bearing “universally” consistent tumor suppressive action against a wider range of tumor targets and with fewer adverse effects in comparison to others? If so, what are the properties that give it an advantage, are they uniquely inherent, or can they be mimicked by specific experimental interventions/adaptations? Are there some MSC/tumor combinations which decisively give better results than others in experimental research work and should therefore be given priority in clinical trials? To what extent does genetic modification of MSC ameliorate the anti-cancer behavior of naïve MSC? Are specific genetic modification methodologies applied on MSC effector cells more efficient than others against tumor targets? Given the uncertainty of naïve MSC-based tumor cytotherapy and on the other hand the more robust performance of genetically modified MSC, should bench research and clinical trials focus primarily on the latter?

In the following sections of this review, we present a strategy for identifying the sources of heterogeneity, for evaluating their relative impact on cytotherapy outcome, and we summarize trends and patterns ultimately using our findings to address the aforementioned issues.

## Methodology of the meta-analysis

Until this day, several reviews have focused on summarizing the aforementioned conflicting results involving the efficacy of MSC in cancer treatment and have, in some cases and to a limited extent, tried to dissect the causal relationship to those discrepancies [84, 121]. The novelty of this review lies in the specific strategy that we adopted for extracting, recording, and organizing experimental parameters, sourced from selected publications in order to, first, investigate associations and patterns concerning reported discrepancies and, second, highlight the optimal conditions for the development of more efficient and reliable protocols for naïve MSC-based cancer cytotherapy.

As part of this effort to elucidate the conditions and factors that could possibly affect the behavior of MSC against tumors, we conducted a small-scale meta-analysis based on information extracted from the literature using a four-step strategy: (1) compilation of a relevant publication library, (2) deconstruction of literature methodology and reported findings, (3) classification and organization of extracted experimental data, and (4) data consolidation and statistical analysis (Additional file 2: Figure S2). In the first stage, the PubMed online bibliographic database was queried for the combinations of keywords “x AND y AND z” (where “x” was “adipose stem cell” or “bone marrow stem cell” or “umbilical cord stem cell” or “Wharton jelly stem cell”, “y” was “mesenchymal”, and “z” was “cancer therapy” or “cancer treatment”), using the following limiters: Humans, English, Journal article, and published from year 2000 onwards. The reference lists generated from the above searches were merged (replicates removed) into a single reference library comprising a total of 861 unique articles, using Endnote X2 citation manager software (Thomson Corp.). The list was then carefully examined manually for specific relevance to experimental cancer cytotherapy studies employing the use of human post-natal stem cells of non-hematopoietic origin, on human tumor cell lines or primary tumor cells. This resulted in a nearly 10-fold compacted library of 108 highly relevant publications whose reported findings formed the basis of our meta-analysis. In the second step, the articles were initially divided into two categories: (a) referring to studies using only unmodified (naïve) human MSC (n-MSC; *n* = 55) and (b) relevant to experimental cytotherapy models utilizing genetically modified human MSC (GM-MSC; *n* = 53). Subsequently, detailed information available in the methodology and results sections of the manuscripts were extracted and recorded in spreadsheets using Microsoft Excel. Strings of information were classified into five broad categories, namely type of stem cells used (effectors) and type of their tumor target (tissue/ cell lines), characteristics of effector-target interface (e.g., dosing, route/timing/method of administration, culture format/animal model adopted), methodology applied for cell modification/labeling and endpoint analyses/assays, and key findings and primary outcome (i.e., pro-/antitumorigenic effect), thus populating a preliminary descriptive database. In the following step, this compartmentalized information was further fragmented and reassigned into over 20 categories (fields) designated “experimental parameters” (see field titles of Additional file 3: Figure S3) to construct a detailed database using Microsoft Access 2010. In this third step, data stratification (expansion of number of categories/fields with simultaneous simplification of field contents) allowed us to recognize individual effector-target combinations with matched primary outcomes, termed “experiments.” More than one such experiments per article were reported in some cases, leading to the identification of a total of 156 experiments, which were allocated in three distinct groups: (1) experiments describing the effect of unmodified (naïve) MSC (*n* = 89), belonging to the first category of articles above, (2) experiments of the second article category which refer to the use of naïve MSC as controls for evaluating GM-MSC anti-cancer efficacy (*n* = 43), (3) experiments (again included in the second article category) that describe the anti-cancer efficacy of GM-MSC without a direct comparison with naïve MSC (*n* = 24). The primary outcomes of these experiments were flagged as tumor promotion or suppression (or neutral, if no significant difference was observed between treatment and relevant controls) depending on the results of the in vitro/in vivo assays as described in the respective publications. In the final step, the experimental parameters were used to derive database queries and to perform statistical analyses, the outcome of which is discussed in the following section.

## Overview and discussion of the meta-analysis’ main findings

### Naïve MSC-based cancer cytotherapy: from trend identification to protocol optimization

#### Relevance of preclinical cancer cytotherapy targeting to global cancer incidence

**Fig. 2:**
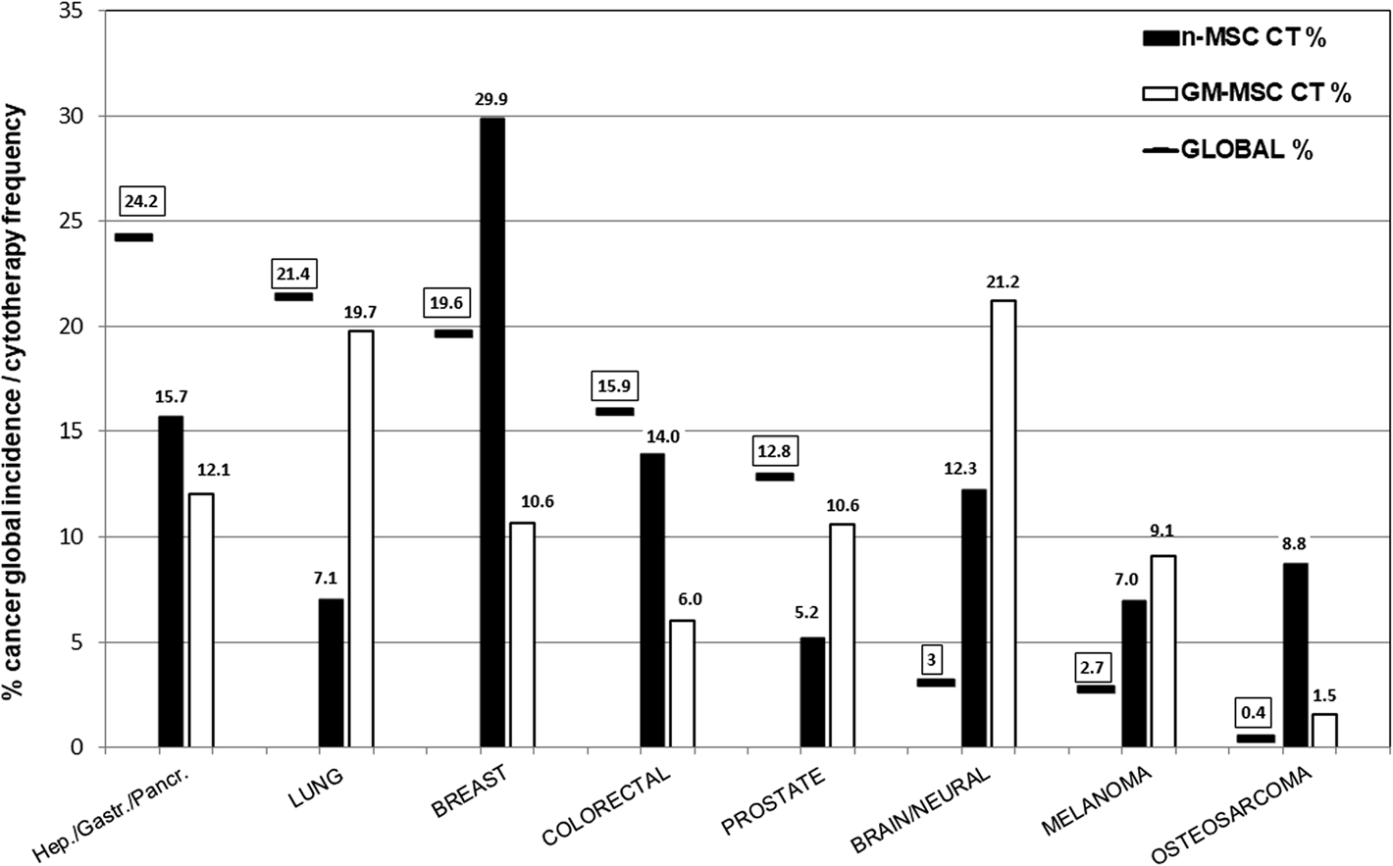
Representation of common cancer types in MSC-based cytotherapy studies compiled in this review in comparison to global cancer incidences (clinical relevance). The most frequently targeted tumors in cancer cytotherapy studies have been ranked in descending order of worldwide cancer incidence (2012 data) [122] from left to right on the *x* axis. Global cancer incidence rates are depicted as solid line symbols (boxed values), while the frequencies of tumors targeted by unmodified/naïve MSC (n-MSC) or genetically modified MSC (GM-MSC; see also the “Genetically modified MSC as delivery vehicles for antitumorigenic molecules—overview and meta-analysis results” section) in experimental cancer cytotherapy (CT), as determined by our meta-analysis, are represented by black (n-MSC CT) or white (GM-MSC CT) columns, respectively. Sample sizes: *N* = 79/*N* = 67 for n-MSC- and GM-MSC-based work, respectively. For each cancer type, the difference in height between the solid line symbols and the column bars denotes the divergence in representation of global cancer incidence by CT work, with positive differences (global % > MSC CT %) signifying under-representation, and negative ones (MSC CT % > global %) over-representation of cancer incidence. For example, the two most under-represented tumors targeted by n-MSC CT are those of lung as well of liver/stomach/pancreas (HGP) (by 12.3% and 8.5%, respectively), while the most over-represented ones are those of breast and brain (difference of − 11.3 and − 8.3, respectively), see the main text (the “Relevance of preclinical cancer cytotherapy targeting to global cancer incidence” section) for further discussion. Overall, the data suggest that in order for experimental CT work to become more clinically relevant, more focus should be put on the following, under-represented tumor targets: * Hepatic/gastric/pancreatic (HGP) tumors (using both n-MSC and GM-MSC). * n-MSC-based lung cancer cytotherapy. * Colorectal cancer targeted by both n-MSC (especially UC-MSC) and GM-MSC. * Prostate cancer targeted by GM-MSC (especially UC-MSC), as well as n-MSC. * GM-UC-MSC-based brain tumor cytotherapy

Statistical analysis of the experimental data extracted from the literature has highlighted eight types of tumors as most frequently targeted in naïve MSC-based cancer cytotherapy experimental protocols (Fig. 2). In an effort to put these tumors into clinical context, they were compared to global incidence [122]. Interestingly, the list of the 10 most frequently targeted tumors has a corresponding total incidence of just over 60% globally; the remaining 40% includes important cancers (cervical, esophageal, bladder, N-H lymphoma) which conclude the top 10 global cancer incidence list, but nevertheless are not the main focus of experimental cytotherapy efforts. Moreover, a more thorough comparison of the data revealed a predisposition of the use of naïve MSC towards specific cancer types, as well as a notable inverse relationship between the frequency of cancer targets used in MSC-based cytotherapy experiments and the cancer types with the highest clinical incidence and mortality worldwide. Thus, while breast cancer with frequencies of 30% is the most popular target and is over-represented in comparison to its global incidence (19.6%), lung cancer, on the other hand, which is the most frequent and one of the most lethal types of cancer worldwide, is greatly under-represented in in vitro/in vivo experimentation (7.1% vs. an incidence of 21.4%). Actually, in cancer epidemiology, nearly one in two cancers are lung or hepato-gastro-pancreatic (HGP) cancers with a top-ranking, combined mortality rate of nearly 90%, yet they comprise less than 25% of cytotherapy targets. It is worth noting that HGP tumors are the only type featuring in the top three most frequently targeted by all three MSC types in cytotherapy studies, albeit with a frequency less than half than they affect patients globally (Fig. 2). Even more impressive is the under-representation of prostate cancer (the second most frequent cancer in adult male population), as well as the absence of studies regarding the use of UC-derived MSC on colorectal cancer. On the opposite side, we have the over-representation of brain tumors (glioma/glioblastoma), which is partly justified by the highly aggressive nature of these cancers (first year prevalence of 1.6%), as well as of melanoma (an extremely rare cancer). This misrepresentation is largely attributed to AT-MSC (Additional file 4: Figure S4).

The shortage of naïve MSC-based research on targeting highly frequent and lethal cancer types clearly necessitates more focused work to be carried out. Moreover, there is an excessive concentration of research efforts utilizing MSC against tumors (e.g., breast), which have failed to produce patient-beneficial results, as it is discussed below.

#### Differential effects of naïve BM-, AT-, and UC-MSC on tumor targets

**Fig. 3:**
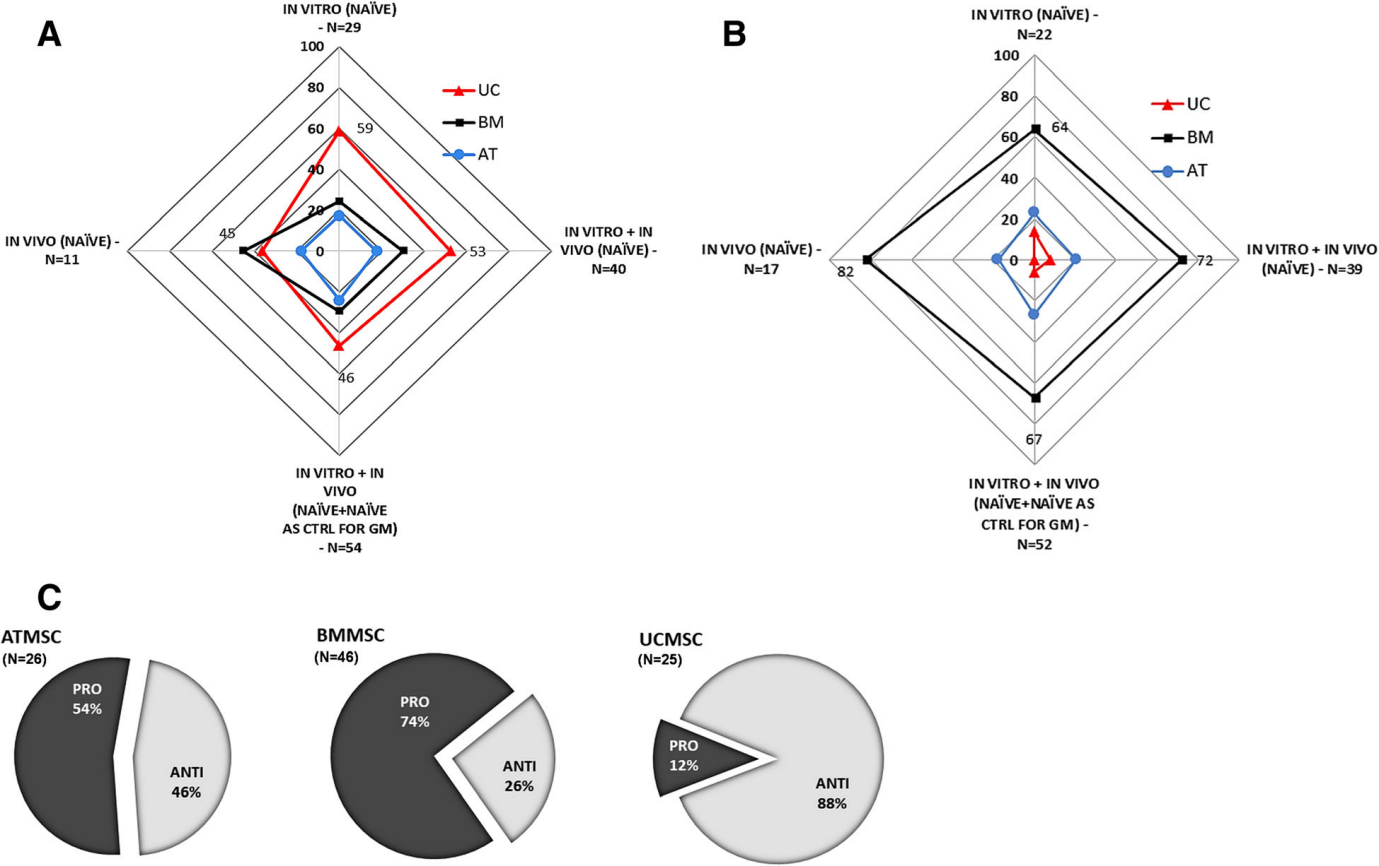
Anti-cancer efficiencies of naïve MSC isolated from adult bone marrow (BM), adipose tissue (AT) or fetal umbilical cord (UC). **a** Radar graph depicting differences in frequencies of UC- (red), BM- (black), or AT-derived (blue) MSC associated with tumor suppressive activity in vivo and/or in vitro. The tumor suppression frequency for each MSC type relative to the other two is represented by vertical distances from graph center. Each outer vertex represents the maximum frequency (100%), equal to the sum of the three relative frequencies of the respective MSC types. Suppression rates are shown for four consolidated groups (presented in ascending order of size clockwise starting from “in vivo”). Values for the highest suppression rate in each of the four groups are shown. **b** Radar graph depicting differences in frequencies of UC- (red), BM- (black), or AT-derived (blue) MSC with respect to pro-tumorigenic behavior in vivo and/or in vitro. The tumor promotion frequency for each MSC type relative to the other two is represented by vertical distances from graph center. Each outer vertex represents the maximum frequency (100%), equal to the sum of the three relative frequencies of the respective MSC types. Promotion rates are shown for four consolidated groups (presented in ascending order of size clockwise starting from “in vivo”). Highest observed values for each of the groups are shown. **c** *Distribution of pro- vs. antitumorigenic effects* for each of the three naïve MSC types in vitro and in vivo (*N* = 26/46/25 for AT-/BM-/UC-MSC, respectively). In the case of UC-MSC, all pro-tumorigenic effects were observed in vitro

Following on from addressing the frequency, distribution and clinical relevance of cancers targeted in naïve MSC-based cancer cytotherapy, we set on to evaluate the anti-cancer behavior and efficiencies of the three MSC types relative to each other. Our meta-analysis showed a differential pattern of anti-carcinogenic behavior, with BM-MSC generally being pro-tumorigenic, and UC-MSC having a more clear onco-suppressive character (Fig. 3c). More specifically, overall, UC-MSC is the MSC type most commonly associated (about 50% frequency) with suspension of development of its tumor targets (Fig. 3a), while concomitantly having the lowest association (< 10%) with tumor promotion (Fig. 3b), which notably is observed only in relation to in vitro experimental work (Additional file 5: Figure S5). On the contrary, BM-MSC promote cancer progression in the majority (~ 70%) of the experiments conducted both in vitro and in vivo, at least twice as frequently as AT-/UC-MSC (Fig. 3b). On the other hand, AT-MSC’s anti-cancer behavior is the least prominent relative to the other two MSC types (20.5% for AT-MSC, vs. 29.5%/49.5% for BM-/UC-MSC frequency of onco-suppressive action towards tumor targets in vitro and in vivo) (Fig. 3a).

**Fig. 4:**
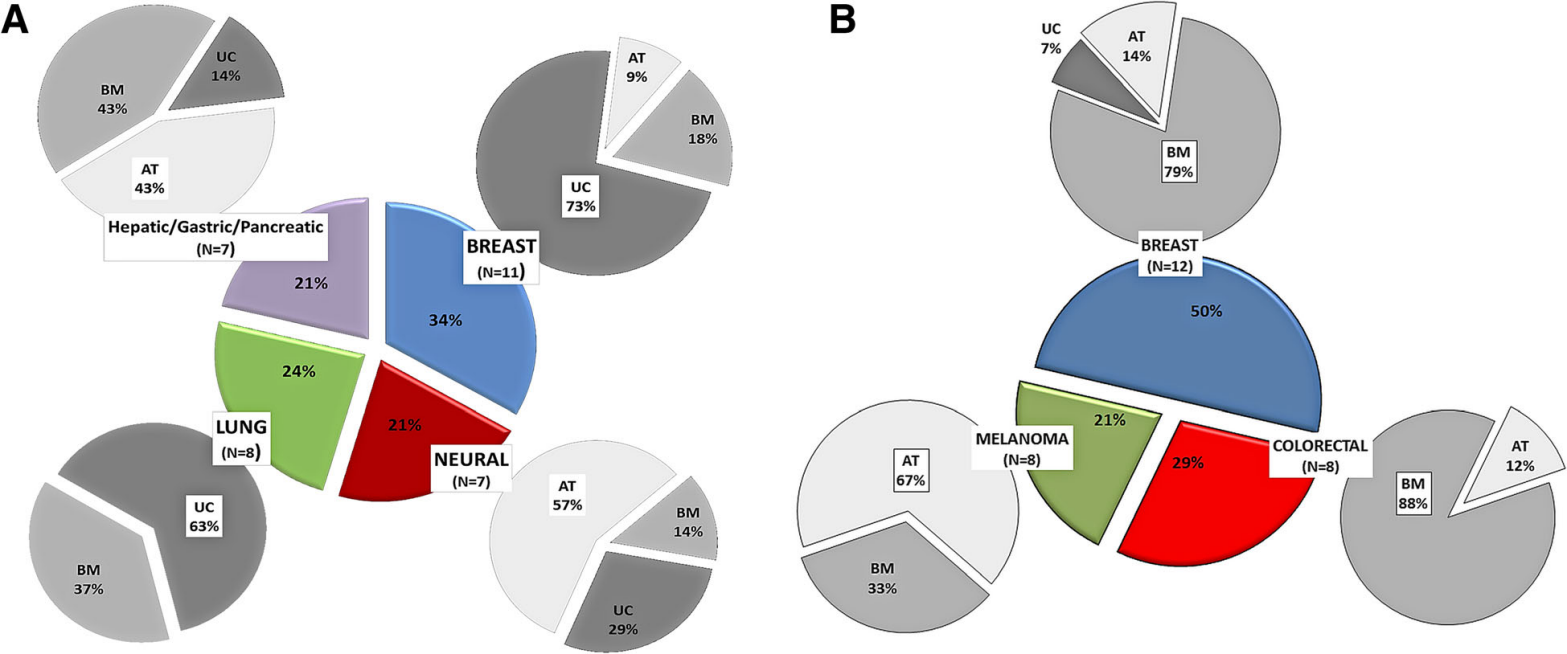
Cancer types most frequently associated with naive AT-/BM-/UC-MSC according to the type of anti-cancer effect produced (suppression or promotion) in vitro and in vivo. **a** The colored central pie chart depicts the distribution of the four most common tumors upon which naïve MSC were found to have a detrimental (onco-suppressive) effect. The percentage of relative association of each MSC type with each of the tumors is represented by the respective gray-scale peripheral pie chart. The type of MSC most frequently associated with its target tissue and the frequency (%) value are highlighted with a white box within the peripheral pie chart. Total sample size = 54 experiments using solely naïve MSC or naïve MSC as controls for GM-MSC-focused work. Only tumor targets with a minimum sample size of seven (*N* ≥ 7) are shown. **b** The colored central pie chart depicts the distribution of the three most common tumors associated with a supportive function (tumor promotion) of MSC. The % relative association of each MSC type with each of the tumors is represented by the respective gray-scale peripheral pie chart. The type of MSC most frequently associated with its target tissue and the frequency (%) value are highlighted with a white box within the peripheral pie chart. Total sample size = 50 experiments using solely naïve MSC in addition to naïve MSC used as controls for GM-MSC-focused work. Only tumor targets with a minimum sample size of seven (*N* ≥ 7) are shown. In the case of colorectal cancer, the absence of UC from the MSC pie is due to the lack of relevant studies, rather than the lack of tumor promotion on the specific target

Switching the focus to the specific types of tumors with markedly affected progression—either positively or negatively—by MSC administration, our meta-analysis revealed the following associations (Fig. 4). First, the tumors most frequently suppressed by naïve MSC in the available cytotherapy in vivo and in vitro studies are those of the breast, lung, and brain, as well as the liver, stomach, and pancreas (Fig. 4a). UC-MSC are mainly responsible for suppressing breast and lung cancers (73% and 63% contribution, respectively), while AT-MSC are associated with glioma/glioblastoma inhibition in the majority of cases (57%), relative to the other MSCs. Second, with respect to tumor promotion, there is a strong association of BM-MSC with breast and colorectal cancer (79% and 88%, respectively) and of AT-MSC with melanoma. UC-MSCs, on the other hand, have minimal or no association with carcinogenesis induction for these three tumors (Fig. 4b). A parallel analysis based on consolidated MSC groups confirmed the pro-tumorigenic effect of BM-MSC on breast and colon, as well as the antitumorigenic effect of UC-MSC on breast and lung cancers, and of AT-MSC on neural tissue tumors (Table 1). Unfortunately, due to sample size constrains, the analysis could not resolve any possible differences between in vivo and in vitro effects. Nevertheless, it validated breast cancer, as not only the most common cytotherapy target but also the only one being both suppressed and promoted by naïve MSC (UC-MSC and BM-MSC, respectively).

**Table 1:** Summary table of main experimental parameters and outcome as determined by the meta-analysis of cancer cytotherapy studies using naïve (unmodified) MSC

Consolidated groups	*N*	MSC	MSC *N*	Tumors most frequently targeted (%)	Tumor target frequency (%)	Mean antitumor effect*
In vivo (naïve)	30	AT	5	Brain/neural	60	ND
		BM	20	Breast	25	ND
				Colorectal	25	ND
		UC	5	Breast	40	ND
In vitro (naïve)	53	AT	10	Breast	30	ND
		BM	22	Breast	32	??
		UC	21	Breast	33	ANTI-
In vitro + in vivo (naïve)	83	AT	15	Brain/Neural	40	??
		BM	42	Breast	29	PRO+
				Colorectal	19	PRO+
		UC	26	Breast	31	ANTI-
In vitro + in vivo x(naïve+naïve as CTRL for GM)	126	AT	31	Brain/Neural	32	ANTI-/NEUTRAL
		BM	59	Breast	24	PRO+
				Colorectal	15	PRO+
				Liver	12	??
		UC	36	Breast	33	ANTI-
				Lung	17	ANTI-

Findings based both on studies focusing solely on naïve MSC, as well as on selected studies where these cells were used as controls for experiments focusing on genetically modified MSC (GM-MSC) are shown. Results are listed in consolidated groups of increasing size (number of observations = *N*). MSC N column depicts MSC sample size. Primary outcomes include percentage of the most frequently targeted tumor types by the respective MSC, as well the mean anti-cancer effect for each MSC (effector): tumor combination. The minimum accepted number of MSC (cut-off) most commonly associated with respective tumors was set to six

*PRO+ denotes tumor-promoting effect exerted by >50% of the associated MSC

ANTI denotes inhibition of tumor by >50% of the associated MSC

?? denotes no clear effect

*ND* not determined due to insufficient data (*N* < cut-off)

**Fig. 5:**
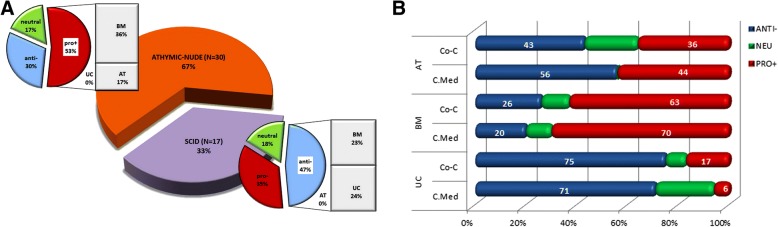
Relationship between adoption of experimental model in vivo (animal model) and in vitro (culture format) and outcome of naïve MSC-based cancer cytotherapy experiments. **a** In vivo. Bar of pie chart categories denote relative contribution of MSCs to the most frequently observed outcome for each animal model. Naïve MSC plus naïve MSC used as controls for GM-MSC-focused work were used to derive total sample size. For experiments on athymic- nude mice, *N* = 30. For experiments on SCID mice, *N* = 17. **b** In vitro. Co-C = direct or indirect co-culture of naïve MSC with cancer cells. C.Med = use of naïve MSC-conditioned media on cancer cells. Sample sizes: *N* = 23/29/29, for AT-/BM-/UC-MSC, respectively

Obviously, the origin of the MSC population used for administration in animal tumor models plays an instrumental role in determining the fate of the treatment. Our analysis has shown that, in terms of anti-cancer efficacy, naïve BM-MSC despite the plethora of studies referring to their use in cytotherapy protocols, perform rather inefficiently. This is supported by the fact that they present the worst in vitro anti-cancer performance as discussed further on, they are frequently associated with tumor promotion both in vivo and in vitro (Fig. 3), and they are the only MSC type with validated tumor-promoting effects against at least three different tissues, including breast and colon (Fig. 4, Table 1). Most notable is the case of breast, in which tumor promotion was verified in vitro using three different cancer cell lines by five independent groups [33, 56, 58, 74, 76]. Furthermore, also worth noting is that, in contrast to BM-MSC, UC-derived stem cells show much greater potential as candidates for cancer cytotherapy. This is supported by the following trends (Figs. 3, 4, and 5 and Table 1); UC-MSC are (1) the only MSC type with no reports of tumor promotion in vivo (regardless of the xenograft model they are used in) and no confirmed reports in vitro in over 30 studies so far). This attribute alone makes the safest candidate for clinical trials for MSC-based cancer therapy. (2) UC-MSC are the MSC type with the most robust antitumorigenic activity (against breast and lung). The association with breast tumor suppression is particularly strong with many independent studies confirming it [69–73]. In comparison, the majority of studies targeting brain tumors using AT-MSC attribute an antitumorigenic behavior to the latter, while naïve BM-MSC, as stated before, have yet no proven therapeutic value. Moreover, with breast and lung accounting for 41% of tumors, the anti-cancer behavior of UC-MSC is clinically relevant too. With respect to the last point made, one should take into account the limited number of studies available on other important tumors such as colorectal, prostate, and liver (see also Fig. 2) that do not allow any sound conclusions to be made regarding the effect of naïve UC-MSC on them. (3) UC-MSC are the only MSC with essentially no contradictory outcome reported (e.g., both pro- and antitumorigenic against the same tumor target). On the contrary, the latter is true for both BM-MSC and AT-MSC (BM-MSC vs. colon cancer in vitro [57, 60]; AT-MSC vs. brain tumors [62–64]). (4) UC-MSC are the only MSC type (as witnessed by in vitro experimentation, discussed further on) that can efficiently act upon its tumor target indirectly through its secretome, without any physical contact or crosstalk with components of the tumor microenvironment.

#### Exploring the role of experimental parameters on MSC-based cancer cytotherapy outcome

As discussed earlier, one of our main hypotheses was that the experimental conditions could be determining factors for the pro- or antitumorigenic behavior of MSC. Thus, technical variation can supplement biological heterogeneity as a co-founding factor for the discrepancies observed in tumor modulation by naïve MSC in cancer cytotherapy protocols. The propensity of MSC to either support or suppress tumor growth is the outcome of the combination of multiple parameters, such as donor-to-donor (epi-) genetic variability, heterogeneity in the isolation of the original MSC population, heterogeneity due to in vitro cell propagation, differences in the characteristics of the in vivo tumor model adopted, divergence in the choice of cell dosing (MSC: cancer cell infusion ratios), and timing of administration (co-infusion vs. MSC administration at early or advanced stage pre-established tumors).

Consequently, the classification of data extracted from studies in the literature as experimental parameters in our database was integral to our meta-analysis methodology approach. The parameters examined included choice of cell culture format/ animal model, cancer cell lines, in vivo cell administration scheme (route and timing), and MSC to cancer cell ratio. This was done in order to investigate the influence of each parameter alone or in combination in shaping cytotherapy outcomes and contributing to heterogeneity (Fig. 5, Tables 2 and 3).

**Table 2:** Contribution of experimental parameters/variables on cytotherapy outcome. The following parameters were examined

A. Tumor suppression vs. cancer cell lines (in vivo/in vitro)								
MSC		Breast cancer cell line						
		MCF-7			MDA-MB-231		All cell lines	
BM/AT/UC		17/ND/*75 (75)*			0/ND/*60 (85.7)*		36/20/*56.3 (82)*	
All MSC		36			46		34.4	
B. Tumor suppression vs. cell administration scheme (in vivo)								
MSC		In vivo cell administration scheme						
		Co**-**injection of MSC + cancer cells			Administration of MSC in established tumors			
BM/AT/UC		11.8 (50)/10 (0)/ND			*64.7 (72.7)/50 (100)/64 (66.7)*			
All MSC		14.3 (25)			61.5 (75)			
C. Tumor suppression vs. MSC: cancer cell ratio (in vivo)								
MSC		MSC: cancer cell ratio (in vivo)						
		MSC: cancer < 1			MSC: cancer ≥ 1			
BM/AT/UC		36.4/*33.3*/71.2			25/*12.5*/66.7			
All MSC		45.8			30			
D.								
Consensus of in vivo cytotherapy experiments								
Effect on tumor	MSC type	Mouse model	Admin. route	MSC dosage (millions)	Ratio (MSC to cancer cells)	Repeated dosing	Effector-target overlap period (days)	Total experiment duration (days)
ANTI- (*N* = 19)	NA	NA	NA	1	1:2	NA	22 ± 19	30 ± 23
PRO- (*N* = 27)	BM	Athymic/nude	s.c./s.c.	1	1:1.35	No repeat	39 ± 22	40 ± 24

**A.** *Type of tumor-specific cell line targeted*. Tabulated values denote breast tumor suppression rates (%) caused by MSC after interaction with various breast cancer cell lines, including the two most common ones, MCF-7 and MDA-MB-231, in vitro and/or in vivo. Νumber of experiments *N* = 11 (MCF-7), 14 (MBA-MB-231), and 32 (all eight cell lines). *ND* not determined due to insufficient data (*N* < 5). Numbers in brackets (UC-MSC) correspond to percentage of suppression if neutral effects are not taken into consideration. Data show a more robust tumor suppressive effect of UC-MSC (in italics) which seems be cancer cell line-independent. **B.** *Timing of in vivo cell administration*. Tabulated values denote percentages (%) of inhibitory effects caused by MSC on tumors in in vivo experiments, in which MSC were administered either simultaneously (co-infused) with cancer cells in animal models or sequentially in established tumors. The number of experiments recorded for the two modes of administration were *N* = 27 and 39, respectively*. ND* not determined due to insufficient data (*N* < 5). Percentages in brackets: sub-portion of samples in which a strong (over twofold) suppressive effect was observed. Data show that MSC have a more prominent suppressive effect when delivered to pre-established tumors. **C**. *Cell dosing ratio*. Tabulated values denote percentages (%) of tumor suppressive effects of MSC in in vivo experiments, depending on the relative number of administered MSC (effector cells) to cancer (target) cells (MSC to cancer ratio). The effects of two such ratios are compared: MSC: cancer < 1 (cancer cells in excess) vs. MSC: cancer ≥ 1 (MSC in excess). Percentages refer to the proportion of experiments with antitumorigenic results out of the total number of experiments in each ratio. The number of experiments recorded for the two ratios were *N* = 24 and 30, respectively. Data indicate a stronger, by over twofold, tumor suppressive effect of AT-MSC when used at the lower MSC dosing ratio (values in italics). See also section 4.1.3 for further discussion. **D.** Combination of the typical, most frequently observed (> 50% frequency) parameters of in vivo experiments using naïve MSC. Typical experimental parameters related to anti- or pro-tumorigenic effect of MSC are shown. *NA* data associated with the respective parameter gave no clear consensus. Highest suppression rates are highlighted in italics (where applicable)

**Table 3:** Possible causes of discrepancies in the antitumorigenic effects of MSC populations

Contributing parameters to outcome disparity of MSC-based cancer cytotherapy	Variability range of differentially expressed parameters	Proposed optimal experimental parameter
MSC isolation source	BM (human fetal or adult, mouse) AT (human), UC (human, rat)	Human UC
MSC in vitro/ex vivo expansion	MSC passage, MSC confluence, high serum or growth factor supplemented media, possible contamination with tumor cells	Determine maximum passage No. for MSC/check senescence status, minimize serum of animal origin
In vivo tumor model	Over 60 cell lines representing 15 tumor types (including sarcoma, hepatoma, adenocarcinoma, melanoma, glioblastoma, lymphoma)	At least two different tumor cell lines per cancer
	SCID, athymic nu/nu mice	Athymic nu/nu mice (or nude variants)
MSC species origin	Syngeneic, xenogeneic	Human xenograft
MSC : Cancer cell ratio	BM-MSC: 2:1–1:1–1:12 AT-MSC: 1:1–1:10 UC-MSC: 6:1–1:1–1:6 (ratios more frequently associated with tumor suppression)	Dependent on MSC type and in vivo or in vitro experiment. 1:1 and 1:2 should be used as starting ratios
Cell administration route	Orthotopic/intratumoral, subcutaneous (s.c.), intraperitoneal (i.p), intravenous (i.v.)	Ideally, i.v. if homing also needs to be demonstrated. Otherwise, orthotopic, good for mimicking human carcinogenesis
Timing (latency) of MSC administration	Simultaneously with tumor cells, successive **(**variable time lag**)**	Successive (lag depends on type of tumor model; usually 7 days)
Repetition of administration	Single (“one-off”) administration or repeated dosing	Repeated (once or twice); doses > 1 week apart

Although we are anything but close to identifying the unequivocally ideal conditions under which MSC will be able to diminish tumor growth, findings that stemmed from analyses of core parameters in our study combined with the state-of-the-art knowledge could help build some generalizations regarding optimal conditions; these are discussed in the following lines and summarized in Table 3.

With regard to the experimental model adopted, our analysis showed that the animal model employed in in vivo cytotherapy protocols, and, in parallel, the cultivation conditions (culture format) of effector and target cells in vitro, could be decisive factors for the fate of MSC-based treatments (Fig. 5). At this point, it is worth highlighting that the in vivo work included in our analysis comprised preclinical cancer cytotherapy models in which human primary MSC were implanted in immune deficient/immunocompromized rodent hosts (SCID or athymic nude mice variants) bearing human tumor xenografts. Syngeneic models or models bearing non-human MSC (which constituted 10–15% of the total) were not taken into consideration. Although this exclusion limited the amount of studies, hence, the number of data available for statistical analysis, it on the other hand constrained heterogeneity attributed to differences in native antitumor immune responses and, most importantly, in homology of expressed molecules between therapeutic cells and target cells [123]. Although both *xenograft models* included in our analyses provide excellent representation of human disease allowing the engraftment, growth, and interaction of human MSC and human cancer cells, the majority (two thirds) of the studies were conducted on athymic mice. The latter bear the advantage of being less immunodeficient than SCID variants, therefore more convincingly recapitulate immune system- affiliated antitumor therapeutic responses and disease progression [124]. As shown above, overall, the vast majority of tumor promotion effects are exerted by BM-MSC (Fig. 3b), while in the case of suppression there is no such clear contribution by a specific MSC type (Fig. 3a). With regard to the animal models used, one would expect tumor promotion to be more frequently observed in SCID mutants (severely deficient in functional B and T lymphocytes), rather than in athymic nude mice, which are less immunocompromized, bearing more active B cells and intact innate immunity through robust natural killer (NK-) cell responses. Yet, it is the majority of experiments (53%) in athymic mice in which tumorigenesis is promoted; largely responsible for this outcome are BM-MSC (Fig. 5a). This could be possibly explained by the immunomodulatory function of MSC which have proved to be able to inhibit T and B, as well as NK cells, suppressing thereby any possible adaptive immune responses [97–99]. Moreover, it has been proposed that active B cells, which in this case may escape MSC targeting, promote acute innate inflammation, which in turn impels malignant progression [125]. Furthermore, in the late 1990s, Barbera-Guillem and colleagues showed that immune complexes formed by antibodies and tumor-associated antigens (TAA) can promote tumor progression, through a mechanism that involves the activation of a crosstalk between polymorphonuclear (PMN) leukocytes and monocytes [126]. From this aspect, the potential interactions between injected MSC and the nude animal’s activated B lymphocytes could trigger tumor progression through a mechanism that is T cell independent. In any case, all these mechanisms might be more prominently activated in the case of BM-MSC, since impressively, as aforementioned, UC-MSC do not promote tumorigenesis in vivo, and this is regardless of the immunological background of the xenograft host. This is most likely attributed to the unique immunomodulatory properties of these cells, with their antigenicity not necessarily triggering tumorigenicity (further discussed in the following section).

*In vitro cancer cytotherapy experimental models* comprise two main types: (a) co-cultures of MSC and cancer cells, where cells communicate (two-way interaction) either directly through physical contact and the formation of gap junctions, or indirectly via exchange of soluble factors (crosstalk), and (b) monocultures of target (tumor) cells, which are grown in and react to culture media in which the effector cells (MSC) have been grown for 24–48 h (conditioned media (CM)). This second culture model basically allows only unilateral signal communication (from the effector to the target/responder cell). In both experimental settings, BM-MSC had mainly pro-tumorigenic effects on cancer cells, while on the contrary, UC-MSC were mostly antitumorigenic (Fig. 5b). In vitro, the frequency of association of UC-MSC with tumor-promoting events is very low (2–12 times lower than in case of adult MSC). More interestingly, the association of UC-MSC with antitumorigenic activity is quite robust (> 70% frequency), irrespective of the culture model adopted. A moderate or strong tumor suppressive effect of UC-MSC-CM has been observed against a group of cancer targets much more diverse than in the case of adult MSC; these include the bladder [83], breast [68, 71], larynx [127], lung [128], glioma [129], lymphoma [130], melanoma [131], osteosarcoma, and ovarian adenocarcinoma [70]. Consequently, UC-MSC bear quite robust anti-malignant behaviors *in vitro*, with their secretome alone being strongly tumoricidal, suggesting that they possess superior inherent anti-cancer properties, being effective solely in a paracrine fashion with a non-essential need for physical cell contact. Taken together with their performance in vivo, UC-MSC exhibit very low probability of eliciting tumor initiation or progression in experimental cancer cytotherapy, thus laying the foundation for their safe inclusion in clinical trials.

With respect to the diversity of tumors targeted, our analysis included over 15 tumor types represented by more than 60 well-characterized *human cancer cell lines*. The cancer types with the highest number of available cell lines were the breast and brain (eight and seven cell lines each, respectively). Nevertheless, it is interesting that only 12% of the studies used two or more representative cell lines to describe and confirm the observed effects on the tumor tested. In an effort to test whether MSC exhibit distinct behavioral imprints on different cell lines of the same cancer tissue, we focused on breast cancers, since not only they were the most popular tumor target, but also the one represented by a large number of cell lines, as stated above. We compared data from two of the most commonly used cell lines, MDA-MB-231 and MCF-7, vs. all eight available breast cancer cell lines (Table 2A). Interestingly, the tumor suppression rate overall did no differ significantly with respect to the cell line but as a function of the MSC used, with UC-MSC having clearly a more profound effect than the other two MSC types. This is despite the differences in the characteristics of the cell lines examined, with MDA-MB-231, a triple negative, aggressive breast cancer cell line, being more prone to cytotoxicity, and MCF-7 more robust and resistant, as evidenced by chemotherapeutic treatments. Thus, although these results are only indicative, it would be logical to assume that for at least some cancers, the biological differences between MSC are a greater source of heterogeneity than the genotypic and phenotypic diversity of cancer cell lines. Nevertheless, it is proposed that the effects of MSC against a given cancer should be examined and verified by at least two cancer cell lines, ideally with distinct characteristics.

Our analysis has also highlighted the *cell administration scheme* adopted (i.e., the route and timing of MSC and tumor cell infusions) as one of the key factors influencing cancer cytotherapy outcome. Cross-examination of the data in Tables 2B and 2D reveals that simultaneous administration (co-infusion) of MSC and cancer cells in studies in rodents results in poor anti-cancer efficacy (14.3% suppression). In sharp contrast, delayed administration of MSC (usually infused orthotopically or intravenously, 7–10 days after initial tumor cell delivery) dramatically ameliorates both the occurrence and severity of tumor suppression (by over fourfold and threefold, respectively). This observation is consistent with the hypothesis that the presence of MSC during early tumor establishment events may actually facilitate processes, such as angiogenesis, which are required for tumor initiation [93].

In terms of *cell dosing*, MSC-to-cancer cells ratios lower than one (< 1) (i.e., cancer cells in excess) seem overall to be better associated with tumor inhibition in vivo (45.8% vs. 30% for ratios > = 1) (Table 2C). Interestingly, the effect is more prominent for adult MSC, especially. AT-MSC, while UC-MSC seem to retain their high effectiveness towards cancer growth irrespective of the cell dosing ranges applied (Tables 2C and 3). In the case of BM-MSC, tumor promotion occurring when relatively high numbers of MSC are used has been found to correspond to the immunosuppressive functions of these cells that are known to be more active at higher MSC numbers but lost at low doses [132, 133] (see also the “Linking MSC phenotypic differences to variation in anti-cancer efficacy” section).

As a final step in our analysis, we attempted to derive a *consensus of experimental parameters* affiliated to either tumor suppression or promotion by recording the frequently observed (> 50% frequency) value in each case. Although the data did not allow a consensus to be built in the first case, a set of parameters were identified that could be regarded, either alone or in combination, as “tumor promotion-statistically associated experimental parameters” in preclinical cytotherapy studies. Such factors are listed in Table 2D. Thus, according to our findings a single co-injection of tumor cells and human naïve BM-MSC at a ratio of 1.5:1 to 1:1, subcutaneously (unnatural tumor growth site) in athymic mice would most likely maximize tumor initiation and growth.

#### MSC isolation and expansion in experimental cytotherapy—sources of variation and implications for clinical trials

The process of in vitro/ex vivo MSC expansion is necessary in order to obtain clinically significant cell numbers. This procedure is nevertheless time-consuming, costly, and poses cell contamination and loss risks, thus ideally the MSC source should allow the relatively easy, high-yield isolation of populations with propagation characteristics that minimize sub-cultivation time.

A large number of studies have unequivocally demonstrated that variations in ex vivo manipulation conditions, such as age and anatomical origin of donor tissue [134, 135] harvesting procedures [136], type of culture medium, including type of constituent growth serum- [137, 138], initial plating and sub-culture seeding densities, and culture period/number of serial passages [139, 140], either separately or in combination [141], can have a major impact on proliferation and other vital processes (stemness/ differentiation, secretion) of all stem cell types, albeit to different extents.

The contribution of MSC expansion on the discrepancies observed regarding their antitumor behavior can only be speculated, since astonishingly, no preclinical cancer cytotherapy study so far has focused on evaluating the effect of variations in sub-cultivation characteristics of a given MSC type on specific cancer types. This would be particularly interesting as it would not only pinpoint the impact of this source of variation on cytotherapy efficacy but also on adequacy and would aid towards the establishment of some rudimentary good manufacturing practice (GMP) standards in light of the clinical trials [142].

With regard to the differences in ex vivo expansion between various MSC types, a significant amount of studies show that they are ontogeny-related, with many underlining the superiority of MSC isolated from fetal umbilical cord tissue, over those derived from adult tissues (bone marrow and adipose tissue) [143–145]. More specifically, the intervascular matrix of the umbilical cord (Wharton’s jelly) contains the MSC population with arguably the best isolation and expansion characteristics among its other substructures (e.g., perivascular and subendothelial tissues) [146–149]. On the other hand, AT-MSC are regarded superior to BM-MSC, primarily due to the relatively easier, higher-yield harvest method, and secondarily due to their enhanced stability over prolonged sub-culture [150–153]. Ιndeed, BM-MSC have the least favorable characteristics in terms of tissue accessibility/abundance for harvesting purposes since their isolation relies on invasive, painful techniques that use aspiration needles to reach their anatomical source (usually the marrow cavity of the pelvic bones) and which are often associated with patient-to-patient variability and donor site morbidity [150, 152]. In sharp contrast, UC tissue is naturally collected during normal parturition, and unless it is stored for banking or research purposes, it is normally regarded as medical waste and discarded [149].

With respect to ex vivo growth kinetics, UC-MSC bear three distinct advantages over adult MSC. First, the isolation procedure results in larger pool of adherent, clonogenic cells (MSC), especially compared to BM-derived MSC. There are various studies reporting a yield of 10,000–15,000 MSC per cm of umbilical cord tissue in the starting culture [137, 146, 149, 154], while this can be further increased by protocol modification [155, 156]. With lengths of cord harvested ranging between 15 and 48 cm, a typical isolation yield would be about 400,000 highly proliferative UC-MSC [157–159]. On the contrary, adherent BM-MSC represent a quite rare population in the bone marrow, estimated at 0.0001–0.001% of total nucleated cells (1–10 colony-forming unit–fibroblast (CFU-F) per million of peripheral blood mononuclear cells) [160]. This actually equates to no more than a few hundred cells per ml of marrow aspirate, with a typical yield not exceeding 5,000 cells per 5 ml of aspirate [139, 161]. Regarding adipose depots, the reported frequency of nucleated cells isolated is at least 0.4 million per milliliter of processed lipoaspirate. However, plating efficiency of these cells varies considerably (0.2–4 %) [161], so actual AT-MSC turnover can range from 0.24–10 million cells for liposuction volumes between 100–3000 mL (or a mean yield of 0.9–1.3 million stem cells from an average 300 ml (= 270 g) lipoarspirate) [152, 162]; this yield can be 500–2500-fold greater than that of BM-MSC [136, 163, 164]. Second, UC-MSC proliferate at a much faster rate, various studies report population doubling times (PDT) in the range of 26–42 h until the fifth passage (which incidentally is the most common sub-culture threshold used) while for adult MSC it is 50–65 h [136, 137, 139, 149, 165, 166], although there are studies reporting PDT exceeding 96 h especially in the case of AT-MSC [143, 167]. This equates to shorter culture periods required to obtain the desired cell yield and thus makes UC-MSC more suitable for clinical scale expansion (e.g., 30–40 days are required to complete five rounds of expansion, as opposed to 2 months in the case of adult MSC) [168]. A yield of about five billion UC-MSC from a single cord can be obtained within 5–6 passages when large five-cell stacks are used for propagation [138]. Third, UC-MSC can sustain their higher proliferation rates for extended time periods. They not only can be serially propagated up to eight or nine times while maintaining a short PDT (< 30 h), but can be additionally expanded up to 25–50 population doubling (PD) (over 20 passages) while maintaining a normal karyotype as well as their immunophenotypical and stemness characteristics [137, 158, 169, 170]. Differences of up to 22 PD have been observed between the proliferative lifespans of BM-MSC and UC-MSC [168]. Impressively, yields up to 10^17^ UC-MSC (40 PD) over a 2-month culture period have been reported (in sharp contrast to only 5 × 10^7^ (8PD) for BM-MSC) [171]. Interestingly, growth in low O_2_ environment (5%) [138, 172] or in xeno-free culture medium [173, 174] further enhances their proliferative lifespan, while extracts of these cells when coated onto cultures plates ameliorate late-passage proliferation of both UC-MSC and BM-MSC [168]. On the contrary, adult MSC show a limited potential for prolonged propagation, being able to maintain only up to 15–25 PD (9–12 passages) in culture [153, 165, 168]. A senescent phenotype, characterized by a plateau in the rate of PD accumulation, a sharp reduction in cloning efficiency, and an increase in positive β-galactosidase staining, has been observed in some cases as early as only six PD/passages [166, 175, 176]. In fact a cumulative number of 20 or more PD and a PDT > 60 h have been highlighted as thresholds for replicative senescence in BM-MSC, with recommendations to use cells at the lowest passage possible, preferably up to fifth to sixth [142, 177, 178].

With regard to the role of MSC expansion in cancer cytotherapy in specific, we could not draw any conclusions, since no detailed information/experimental data were available (besides number of passages, in some cases) regarding status of cells utilized (such as number of cumulative cell PD, PDT, CFU-F scores, senescence status). However, taking into consideration the growth kinetics and bearing in mind that the optimal MSC: cancer cell ratio should be 1:1 to 1:2 (Table 3), one could attempt to estimate the feasibility of scale up for clinical use for each type of MSC. Assuming that both a tumor (e.g., T1 palpable breast cancer) and a cancer cell (breast cancer cell lines) are spheroids with diameters of 2 cm [179] and 15 μm [180], respectively, one would need 12–24 billion MSC to treat a single tumor. According to our estimations, starting from isolation, AT-MSC would require seven serial passages over a month’s period in order to generate this amount of cells. On the other hand, UC-MSC after 3.5 weeks and the same number of passages would accumulate 19 PD which would be sufficient for treating 8–16 tumors. Last, the ex vivo expansion characteristics of BM-MSC make these cells the least favorable for clinical use. The eight rounds of sub-culture required (equal to 22 PD) are not only the longest (at 56 days, essentially making very difficult to match any need for frequent, repeated dosing) but would also bring the cells close, if not beyond, their senescence threshold. Actually, presuming that this threshold is five to six passages (15–16 PD), then the maximum yield that BM-MSC could give is 100 times less than that required in our example, i.e., these cells can support protocols which require up to approximately 120 million cells.

### Linking MSC phenotypic differences to variation in anti-cancer efficacy

In the introductory section, we gave a brief account of the characteristics that MSC share and form the basis of their adoption as valuable cytotherapy tools. Nevertheless, our literature meta-analysis has revealed that with respect to anti-cancer efficacy it is their differences in genotypic and phenotypic attributes that are more important. This notion is based on the observation that the most prominent disparity in the outcome of cancer cytotherapy is MSC (effector)-related. Some naïve MSC populations are more effective against specific tumor targets, where others underperform and may even prove deleterious for cancer treatment. A prime example of the latter case is the pro-tumorigenic effect of BM-MSC and AT-MSC on metastatic breast cancer. On the contrary, UC-MSC show an anti-carcinogenic response that is both strong and robust, i.e., less sensitive to experimental conditions such as choice of ex vivo model and cell dosing. According to our findings, based on the data available so far, fetal-derived UC-MSC are safer to use and more frequently associated with suppression of tumor growth than MSC derived from human adult tissues. This is backed up by comparative studies evaluating the anti-cancer effects of MSC of different developmental origin [64, 129], although clearly more such studies should be carefully designed and carried out, in order to validate the notion that outcome of cancer cytotherapy is primarily MSC ontogeny-related.

There is sufficient evidence in the literature suggesting that the unique combination of the biological traits suited for giving UC-MSC their special role in human development actually allows them to serve a second purpose as naturally anti-cancer capable cytotherapy agents. UC-MSC are primitive cells falling in the developmental map between hESC and adult stem cells [181]. They share biologic characteristics with both stem cell types; however, they do not combine them with attributes that pose as safety risks for cytotherapy. Thus, similar to hESC and unlike adult MSC, they are consistently positive for *pluripotency and self-renewal markers* such as Oct-4, NANOG, LIF, SSEA-1, SSEA-4, Tra-1–60, and Tra-1–81 [116, 117, 182, 183]. In opposition to hESC, they do not form teratomas when injected in SCID mice [72, 154]. Moreover, as discussed earlier, they are capable of sustaining high proliferation rates for extended periods in culture (due to their longer telomeres and expression of telomerase), while simultaneously maintaining anchorage dependence, contact inhibition and serum dependence as growth requirements, i.e., showing no signs of *spontaneous transformation* [149, 154, 184, 185]. In contrast, human adult MSC have been linked to malignant transformation as well as karyotypic instabilities and epigenetic damage, raising safety concerns [176, 186, 187]. These can arise as a result of extensive ex vivo cultivation or prolonged exposure to tumor cell-generated stimuli. For example, spontaneous malignant transformation occurred in 45.8% of long-term (5–106 weeks) cultures of human BM-MSC, resulting in multiple fast-growing lung deposits when injected into immunodeficient mice [188]. Furthermore, in contrast to UC-MSC, a series of studies have provided evidence for transition of BM-MSC to a TAF-like myofibroblastic phenotype and hence contribution to tumor growth after long-term exposure to conditioned medium of breast and ovarian carcinoma cells [35, 89, 189]. Last, noteworthy is the implication of BM-MSC as originating cells for sarcomas, most notably the highly metastatic osteosarcoma [190, 191]. Nevertheless, there is also evidence in support of the view that adult MSC are safe for cytotherapy applications. With respect to BM-MSC, Bernardo and colleagues did not observe any sign of transformation or genomic instability after 25 passages/44 weeks in culture, respectively [192]. Apparently, this is attributed to their inability to escape replicative senescence due to their short telomeres [193]. Similarly, spontaneous transformation that was initially reported in human primary AT-MSC, following long-term (4–5 months) in vitro culture, was later recognized as an artifact attributed to unnoticed minimal cross-contamination with a fibrosarcoma cell line cultured simultaneously in the same laboratory [194]. Last, a meta-analysis comprehensively summarizing the safety of 36 clinical trials using intravascular delivery of adult MSC for the treatment of non-neoplastic diseases did not find a significant association between MSC use and the appearance of malignancy [195]. Although the occurrence of malignancy as a long-term adverse event in response to systemic MSC infusion was as high as 24% (in non-randomized control studies), the difference compared to control patients was not significant, while none of the malignancies observed were formed de novo.

Our analysis has provided quantitative evidence for the poor anti-cancer performance of BM-MSC which are the MSC type most frequently (74%, Fig. 3c) associated with tumor promotion overall; moreover, their *secretome* scores very low in terms of tumor suppression (20%, Fig. 5b) which means that these cells lack intrinsic tumoricidal properties. On the contrary, UC-MSC bear strong tumoricidal abilities that are both intrinsic, i.e., naturally occurring without the need for cellular crosstalk, and extrinsic, i.e., arise after interaction with malignant tumor cells, dead tumor cells, or other benign cellular components of the tumor supportive stroma [196]. In both cases, the effects can solely occur in a paracrine manner, which nevertheless does not preclude direct physical cell contact, whilst they involve the secretion of multiple proteins (secretome). These include molecules that induce cell cycle arrest and cell death (e.g., tumor suppressors, caspases) on cancer cells [117], as well as variety of immunomodulatory effectors (e.g., pleiotropic cytokines and growth factors) [197–199] with mainly anti-inflammatory action. The antitumorigenic effect of UC-MSC secretome preparations (in the form of cell lysates or conditioned media) has been assessed on a variety of cancer cells in vitro (discussed in the previous section), most often through evaluation of their contribution to cell cycle arrest and apoptosis induction, e.g., by determining changes in expression/activation status of tumor suppressors (e.g., p53) [127, 130], initiator and executor caspases (caspase-9 and caspase-3) [8, 129, 130, 200], and negative/positive apoptosis regulators (Bcl-2, Survivin/Bax) [70, 127, 129, 130]. Upstream apoptosis-mediating mechanisms shown to be involved include JAK/STAT signaling [200] as well as regulation of Akt phosphorylation [83] and intracellular H_2_O_2_ production [130]. In fact it is possible that UC-MSC secretome affects the regulation of key antioxidant enzymes resulting in high levels of intracellular H_2_O_2_ in cancer cells, production of reactive oxygen species, and increased oxidative stress triggering apoptotic cell death, an anti-cancer mechanism similar to that used by many current chemotherapy drugs. Indeed, when UC-MSC extracts are used in combination with doxorubicin, not only they do not increase resistance to the drug, but they also display an additive cytotoxic effect on target cancer cells [201, 202].

Lately, it has become evident that MSC secretome-mediated effects on cancer cells implicate the delivery of bio-molecular cargo (such as mRNA, miRNA, and secreted proteins) packaged into membrane structures called *extracellular vesicles (EV).* MSC-derived EV (MSC-EV), which mainly comprise the nano-scale, endosome-derived exosomes, and the larger microvesicles, are considered as potent regulators of intercellular communication. The main biological potency of MSC-EV is maintenance of tissue homeostasis and mainly involves targeting housekeeping biochemical activities; however, they are also believed to play an important role in the dissemination of cancer phenotype. Data so far suggest that MSC-EV are an integral part of autocrine and paracrine signaling events modulating expansion of the tumor microenvironment, angiogenesis, and metastasis. Little is known about the exact mechanisms but data so far are indicative of complex regulation involving antagonistic effects [203, 204]. Thus, not only MSC-EV cargo composition seems to vary depending on the MSC source ultimately having a differential impact on tumor fate [205], but even more interestingly EV isolated from the same MSC source can have opposing effect depending on the type of tumor target [83, 206].

With respect to the immune system-affiliated antitumor effect of MSC, our meta-analysis did not allow us to make any clear deductions regarding the differences between fetal and adult MSC, since for the reasons explained earlier we chose to exclude data derived from syngeneic models which have proper antitumor responses (tumor infiltrating lymphocytes, macrophages, and myeloid-derived immuno-suppressor cells (MDSCs)) and better represent the contribution of the host microenvironment to the triadic interaction of MSC: cancer cells: stromal supportive cells. Nevertheless, the immunomodulatory properties of fetal and adult MSC have been quite well documented in comparison [105, 207, 208]; moreover, some excellent studies employing syngeneic models provide an insight into the immunomodulation-mediated tumor suppressive effect of UC-MSC [209–211].

Differences in *immunomodulation* are believed to be directly linked to the duality of results in experimental cytotherapy of cancer. For example, polarization of BM-MSC (through specific priming of Toll-like receptors (TLR)) into proinflammatory or immunosuppressive phenotype directly correlates to tumor suppressive or tumor-promoting effects, respectively [104, 212]. The immunomodulatory effect can be so powerful that can actually drive two opposing responses within the same MSC tumor model [213]. With respect to differences in immunomodulation properties of adult and perinatal MSC, three main assumptions can be made by examining the available literature; (1) the immunosuppressive properties of human UC-MSC are significantly stronger than those of BM-MSC. (2) MSC respond to different thresholds of inflammation depending on the type and concentration of proinflammatory cytokines they are exposed to by regulating, in turn, the cytokine secretion and activation marker profiles on immune cells. (3) With respect to activation of immune cells, mitogen-induced and alloantigen-driven lympho-proliferation are modulated probably by different mechanisms, which are unique for BM-MSC and WJ-MSC. The evidence that points towards these conclusions originates mainly from in vitro work. Unstimulated UC-MSC exhibit a strong suppression of lympho-proliferation, even at a very low dose (1% UC-MSC, mononuclear cells (MNC)) compared to BM-MSC, independent of the inductive stimuli (presence of mitogen or alloantigen) [214]. Priming of UC-MSC (but not BM-MSC) with inflammatory stimuli (e.g., IFN-γ) further attenuates lympho-proliferation in a mixed lymphocyte reaction (MLR) containing allogeneic peripheral blood MNC. Nevertheless, mitogen-induced suppression of lympho-proliferative responses is further enhanced upon exposure of BM-MSC (and not UC-MSC) to inflammatory stimuli. UC-MSC (along with AT-MSC) suppress both mitogenically and allogeneically activated proliferation of purified T cells better than BM-MSC in a dose-dependent manner by secreting high levels of LIF; furthermore, they maintain and promote the expansion of T regulatory cells (T-regs) independently of the MSC/T cell ratio [183]. Last, with respect to B cell activation, UC-MSC when co-cultured with PHA-stimulated MNC do not influence acquisition of lymphoblast characteristics by B cells or their progression from non-activated to early activated stage, in contrast to BM-MSC and AT-MSC that exert an inhibitory effect [215].

The differences in immunomodulatory function between adult MSC and UC-MSC are not restricted to adaptive immune responses. Raicevic and colleagues have demonstrated a lower reactivity to bacterial or viral infections involving innate immunity mechanisms. In MLR containing CD3+ T cells, UC-MSC resist the neutralizing effect of inflammation or TLR ligation on their immunosuppressive properties by lacking TLR4 expression and by overproduction of HGF (almost 10-fold higher expression compared to BM-MSC and AT-MSC) [216].

Recently, the unique immunomodulatory properties of UC-MSC have been linked to secreted components of their ECM. In a study involving a mouse corneal transplantation model, Coulson-Thomas and colleagues showed that UC-MSC actively suppress inflammatory immune response of the host microenvironment by favoring M2 macrophage phenotype (anti-inflammatory) over M1 (proinflammatory) and by inducing maturation of T-regs and inflammatory cell death. They do this by secreting components of their unique glycocalyx which constitutes the characteristic matrix in which the cells naturally reside and function [217]. One of the properties with high practical utility in terms of cytotherapy is their *low immunogenicity*, which is a key requirement for allogeneic transplantation. UC-MSC/WJ-MSC, like their adult counterparts, evade immune recognition by lacking co-stimulatory molecule expression (CD40L, CD80/CD86) which are normally implicated in activation of both T and B cell responses. However, crucially, UC-MSC distinguish themselves from adult MSC with respect to expression of major histocompatibility complex (MHC) molecules. UC-MSC express significantly lower levels of MHC class I (HLA-ABC) both at rest and following stimulation with IFN-γ, while MHC class II (HLA-DR) induction with IFN-γ treatment is substantially augmented in BM-MSC but is very negligible in WJ-MSC [169, 218]. The lower HLA class I and II expression profile of UC-MSC leads to dampened immune recognition and promotion of immune ignorance in vivo. It may also partially explain the lack of sensitivity of responder cell proliferation to UC-MSC doses, unlike BM-MSC where the balance between their immunosuppressive/-supportive properties is more emphatically determined by their direct environment (relative cell ratios and physical contact). Indeed, the fact that low MSC numbers may display a stimulatory instead of inhibitory activity on T cell proliferation has been shown using mixed lymphocyte cultures [207]. BM-MSC may express MHC class II antigens and act as antigen-presenting cells eliciting a strong T cell response in the presence of low levels of IFN-γ, i.e., in an inflammatory setting [219]. The expression of MHC class II molecules on the surface of MSC requires autocrine stimulation by endogenous, low levels of IFN-γ, but is decreased at high IFN-γ levels causing the loss of the antigen-presenting cell function of the MSC HLA-DR [220]. Interestingly, in a more recent study, it was shown that HLA-DR-negative BM-MSC can augment or hinder the proliferation of T lymphocytes depending on the cell ratio used. Optimal MSC-mediated inhibition required a high MSC: CD3+ ratio (1:4) and direct physical contact between the cells [183].

Human UC-MSC have been also found to differ from adult MSC with respect to the expression of HLA-G, a non-classical HLA class I molecule which plays a key role in maternal tolerance for the developing fetus [170]. UC-MSC, in contrast to BM-MSC, express high levels of HLA-G which upon IFN-γ stimulation increase even further. This high expression of soluble as well as membrane-bound HLA-G isoforms allow UC-MSC to suppress immune reactions by reducing allo-proliferation of T cells, by disturbing the cytolytic function of NK cells, and by preventing maturation of dendritic cells [221, 222]. HLA-G has been shown to mediate the suppressive function of UC-MSC on lympho-proliferation in MLR and thus their escape from immune recognition by allogeneic peripheral blood lymphocytes [169]. The immunomodulatory activity of UC-MSC has been further demonstrated by their ability to suppress the expression of proinflammatory cytokines in vitro (such as TNF-α and IL-6) after co-culture with peripheral blood MNC, and in vivo by their survival as xenotransplants in immunocompetent mice not receiving any immunosuppression.

Despite their immunosuppressive properties and low immunogenicity, under certain circumstances, UC-MSC can elicit an immune response which should be taken into consideration when establishing a cytotherapy protocol (e.g., timing of MSC administration). In a clinically relevant miniature swine model, while a single injection of MHC-mismatched inactivated UC-MSC did not elicit a detectable immune response, repeated injections in the same region caused the opposite effect, as evidenced by the production of alloantibodies within 1 week [223]. Therefore, care must be taken in order to avoid sensitization, especially against orthotopic cancer cytotherapy.

Overall, ontogeny-related differences in the pattern of expression of HLA molecules (e.g., HLA-DR, HLA-G), as well as in the cytokine secretion profile (e.g., HGF, LIF, IL-6) between adult MSC and UC-MSC are largely responsible for the course of direct or indirect interaction with cellular components and bioactive factors of the tumor microenvironment driving the adoption of a tolerogenic/immunosuppressive or a more immunoreactive/proinflammatory phenotype, which ultimately determines the impact on tumor progression. With this in mind, the optimal dose, frequency, and timing of MSC administration need to be carefully considered for each cancer target.

### Genetically modified MSC as delivery vehicles for antitumorigenic molecules—overview and meta-analysis results

Tumor specificity is the major obstacle hampering the effectiveness of current cancer treatment therapies, such as combination chemotherapy. Advanced drug targeting of tumor cells is particularly hard when treating highly invasive and/or metastatic tumors such as glioblastoma or pulmonary cancer. Uncontrolled drug distribution in the patient’s body, i.e., insufficient concentration at the tumor site and toxic concentration in normal cells, which is attributed to inefficacy of anti-cancer therapy, is often the direct cause not only of severe side effects but also, ironically, of life-threatening complications. MSC tropism towards primary and metastatic tumor locations, which occurs independent of tumor type, MSC immunocompetence and route of delivery, combined with their relatively ease of in vitro manipulation to express molecules with anti-cancer action, naturally provides a solution to drug specificity, bypassing the main issues of stability (short half-life), dosing (effective dose vs. maximally tolerated dose), and off-target toxicity (side effects) associated with their systemic administration. Historically, the therapeutic value of this unique “Trojan horse/smart bomb” approach for the controlled release of potent antitumorigenic agents in situ has been rather convincingly demonstrated in a large number of studies with genetically modified MSC (GM-MSC) targeting an extensive range of cancers in various experimental animal models [reviewed recently by [224, 225].

In this review, we have concentrated on studies referring to human tumor cells specifically targeted by human GM-MSC of adult bone marrow, adipose tissue, and fetal umbilical cord matrix tissue origin, herein termed GM-BM-MSC, GM-AT-MSC, and GM-UC-MSC, respectively (Additional file 6: Figure S6).

Our meta-analysis has revealed that the focus of GM-MSC-based cancer gene therapy experiments is on tumors difficult to treat, i.e., highly invasive and metastatic cancers (Fig. 2 and Additional file 6: Figure S6). Indeed, one of the most frequently used models is that of metastatic lung cancer (19.7%) which is in alignment with highest incidence and mortality of this type of cancer worldwide. Brain tumors, which are practically incurable, feature also quite often as targets in GM-MSC-based work (21.2% frequency) essentially serving as excellent proof-of-principle studies. Nevertheless, GM-UC-MSC are clearly under-represented in relation to brain cancer cell-mediated gene therapy. Indeed, according to our analysis there is a bias (also observed in the case of naïve MSC-based cytotherapy) in favor of BM-MSC (48% frequency of use) with only one in five (21%) of GM-MSC-based studies actually employing the use of UC-MSC over adult MSC. Interestingly, breast cancer’s overall popularity as a target in in vivo anti-cancer work employing GM-MSC is more than threefold reduced (to 11%) relative to naive-MSC-based experiments, probably justified by the strong performance of naïve UC-MSC against this type of tumor. Also in contrast to naïve MSC, the range of tumors targeted in GM-MSC-based cytotherapy is somewhat more limited. Sarcomas (Kaposi’s, osteorsarcoma), lymphomas (Burkit’s, non-Hodgkin’s), and bladder and larynx tumors are notable omissions in GM-focused work.

In terms of anti-cancer efficacy, a number of studies have convincingly demonstrated the therapeutic potential of GM-BM-MSC against lung [33, 226–230] and brain [231–237] cancers and of AT-MSC and UC-MSC on brain [238–241] and breast [72, 73, 200, 242] tumors, respectively (Additional file 6: Figure S6). With regard to GM-AT-MSC and neural tissue tumors, the robust anti-cancer performance of the former counterbalances the erratic behavior of their unmodified precursors, while in the case of breast cancer and UC-MSC the results obtained using GM protocols solidify the already encouraging results obtained using naïve UC-MSC.

#### Overview of GM strategies applied

High clinical relevance of tumor targeting in GM-MSC-based cancer gene therapy is especially important since the latter has showed consistently high anti-cancer efficacy in terms of inhibition of local tumor growth, suppression of metastasis, or prolongation of animal survival, irrespective of the experimental methodology. With regard to the latter, apart from the effector (MSC)-target (tumor) components, the choice of transgene (anti-cancer agent) that is incorporated into the MSC carrier, as well as the enabling genetic modification technology, complete the list of methodological components. The two main strategies adopted are (a) the rather straightforward overexpression of molecules with known antitumorigenic action or (b) gene-directed enzyme/prodrug treatment (GDEPT) (Table 4). In the first case, MSC are transduced with molecules which upon expression are usually secreted acting via different mechanisms on tumors and range from immune regulators such as interferons (e.g., IFN-alpha, IFN-beta, IFN-gamma) [33, 128, 200, 227, 229, 243–245] interleukins (e.g., IL-12, IL-21) [246–248] and chemokines (e.g., CXC3L1) [228], to molecules with pro-apoptotic (e.g., sTRAIL) [226, 232, 234, 235, 249–253], anti-angiogenic (e.g., Alpha1-anitrypsin, NK4) [254, 255], or other properties (e.g., PEDF, TNF-a, HNF4-a) [54, 237, 256–258]. Among these, immune response modulators and pro-apoptotic agents are the most popular transgene classes used, with an adoption rate exceeding 60% (Table 4). The most frequently adopted representatives, IFN-β and TRAIL, which together account for more than 40% of all human transgenes used in GM-MSC-based experimental cancer cytotherapy, are incorporated with the aid of viral vectors (usually adenovirus and lentivirus, respectively) into BM-MSC and UC-MSC markedly enhancing their anti-cancer efficacy.

**Table 4:** Overview of the main MSC genetic modification (GM) strategies and their most commonly used constituents, as utilized in experimental cancer cytotherapy studies (*N* = 67)

GM strategy	Typical GM components				
	Antitumor agent(s)	Adoption rate (%)	Gene introduction vector *	Cellular vehicle *	Tumor target *
Antitumorigenic transgene overexpression	Anti-proliferative immune regulators (e.g., IFN-β)	41	AV	BM-MSC	Various
	Pro-apoptotic molecule (e.g., sTRAIL)	20.5	LV	Various	Various
Gene-directed enzyme prodrug therapy (GDEPT)	Cytosine deaminase (CD) + 5-fluorocytosine (5-FC)	18.6	RV	AT-MSC	Various
	Herpes simplex virus thymidine kinase (HSV-tk) + ganciclovir (GSV)	8.5	RV	AT-MSC	Brain

*Most frequently observed (> 50% frequency) parameters

Tumor necrosis factor-related apoptosis-inducing ligand *(TRAIL)* is a type II transmembrane protein of the tumor necrosis factor (TNF) family of ligands that is expressed by a variety of immune cells and is capable of initiating apoptosis through engagement of its receptors which are differentially expressed in normal and cancer cells [259]. The attractiveness of TRAIL as a therapeutic agent for the treatment of experimental cancer lies in its ability to selectively induce apoptosis in a broad spectrum of tumor and transformed cells, but not most normal cells. TRAIL initiates a caspase 8-dependent apoptosis signaling cascade by forming homotrimers that bind cognate agonistic receptors DR4 (TRAIL-RI) and DR5 (TRAIL-RII) bearing a conserved death motif on target neoplastic cells. Apart from the membrane-bound form, TRAIL can also exist and function as a soluble ligand. Its extracellular C-terminal domain can be proteolytically cleaved from the cell surface in a vesicle-associated or in a soluble form (sTRAIL) that is also able to oligomerise and trigger p53-independent apoptosis via binding to the cytoplasmic part of DR4 and DR5 on target cells [260]. And since most chemotherapeutic drugs and radiation act by inducing tumor cell apoptosis in a p53-mediated manner via activation of the intrinsic mitochondrial pathway, sTRAIL can be used complementarily to overcome resistance to conventional chemotherapy and radiotherapy. Nevertheless, clinical relevance of soluble TRAIL (sTRAIL) has been hampered by its short (< 1 h) pharmacokinetic half-life in plasma, suggestive of relatively low protein stability, as well as by its scavenging/partial elimination through binding to antagonistic decoy receptors (DcR1 and DcR2) expressed mainly by normal tissues which limit its availability at tumor sites [261]. Again, as in the case of IFN-β, local homing and delivery of TRAIL via GM-MSC can overcome these problems. Indeed, GM-MSC have been shown to successfully target and significantly reduce tumor growth in models of multiple myeloma [250], glioma/glioblastoma [232, 234, 235], lung [226, 249, 253], liver [229, 252], colorectal [251], and cervical [249] cancers. Among these are BM-MSC and UC-MSC carrying human recombinant sTRAIL against glioma/glioblastoma [232, 234] and hepatocarcinoma [252], respectively. The significance of TRAIL as an antitumor agent is highlighted by its inclusion in one of the very first clinical trials involving MSC-based cancer gene therapy (NCT03298763; [262]). Set to begin in 2018, this phase I/II clinical trial focuses on the evaluation of safety and antitumor activity of allogeneic BM-MSC-TRAIL on up to 46 metastatic non-small cell lung cancer (NSCLC) patients who are receiving GM-MSC cytotherapy in conjunction with conventional chemotherapy with pemetrexed/cisplatin. Interestingly, the trial addresses the issues of BM-MSC adequacy and variability by pooling cells from multiple donors before transduction [1].

In the second strategy, *GDEPT (also known as suicide gene therapy*), GM-MSC act as carriers of genes encoding for specific enzymes that convert non-toxic prodrugs into active derivatives [263]. The prodrugs are administered systemically following in vivo infusion of GM-MSC and homing towards tumor sites. The cytotoxic effect of the activated drug is then exerted locally on cancer cells at the tumor site, thus minimizing off-target toxicity; of course the production of drug metabolites is also highly toxic for the MSC carriers themselves, which die in the process. The two enzyme/prodrug combinations most widely used are herpes simplex virus thymidine kinase gene (*HSV-tk)/ganciclovir (GSV*) and *Escherichia coli* cytosine deaminase (*CD)/5-fluorocytosine (5-FC*), with suicide genes introduced into the stem cell carriers via viral vectors (most often retroviruses) (Table 4). One obvious advantage of GDEPT over conventional cancer gene cytotherapy, apart from the conditional rather than constitutive type of action of the tumoricidal mechanism, is that it allows the elimination of disseminated cells, thus acting as a safety mechanism against any potential adverse effects (e.g., transformation events) related to the long-term persistence of homed and non-homed MSC in the patient’s body. Most importantly, the main benefit of this anti-cancer approach is the amplification of the drug toxic effects by means of *bystander effect* which is described as the death of non-transfected cells (e.g., cancer cells) due to indirect effects caused by their neighboring transfected cells (MSC), causing more widespread cell death than if transfected cells alone were killed [264, 265]. A requirement for this phenomenon is the active or passive transference of the activated toxic metabolite to neighboring cells which involves the use of gap junctions [266, 267], endocytosis of apoptotic vesicles [265, 268], or paracrine effects that lead to immune system stimulation [269, 270].

The two main GDEPT systems differ to the mechanism by which they accomplish bystander effects and consequently diverge in terms of their tumor growth suppression efficiency. In the case of CD/5-FC system, activated (deaminated) 5-FU rapidly crosses the plasma membrane by passive diffusion and exerts its cytotoxic effects by interfering with both DNA and RNA synthesis. In contrast, the active phosphorylated form of GCV is a charged metabolite unable to readily diffuse out of the cell and thus relies on physical contact and gap junctional intercellular communication (GJIC) between therapeutic and tumor cell to exert its cytotoxic action by interfering with DNA polymerase function. In the case of CD/5-FC system, yeast or *E. coli*, the CD gene can be used for the activation of 5-FC, although the bifunctional fusion protein cytosine deaminase to uracil phosphoribosyltransferase (CD::UPRT) is preferred due to the higher bystander effect achieved in vitro and in vivo. CD::UPRT chimeric protein not only exhibits an enzymatic activity 10-fold greater than that of wild type CD and increases sensitivity to 5-FC by at least 100-fold, but also alleviates cytotoxicity resistance exhibited by some tumors [271, 272].

Various studies have attempted to compare the efficacy of CD::UPRT/5-FC and HSVtk-MSC/GCV systems (in the absence or presence of MSC carriers). Whereas in the first case, CD::UPRT/5-FC proves superior due to its greater bystander effect, the effect on tumors targeted by CD::UPRT-MSC/5-FC or HSVtk-MSC/GCV differs depending on the expression status of enzymes of nucleotide metabolism and ATP-binding cassette (ABC) transporters, irrespective of GJIC status [239, 273, 274]. Thus, for example, while both systems are equally effective against 8-MG-BA gliomas, on the contrary, A375 melanoma and MDA-MB-231 breast adenocarcinoma cells are differentially sensitive to the two systems, with the former being more susceptible to CD::UPRT-MSC/5-FC and more refractory to HSVtk-MSC/GCV treatment, and vice versa.

TK/GCV and CD/5-FC GDEPT comprise the minority of GM-MSC-based strategies, almost exclusively using adult MSC as cellular vehicles (with AT-MSC used in three out of four cases). Cancer types targeted include melanomas [275], sarcomas [276], colon [277], prostate [67, 278, 279], and ovarian [274]. The TK/GCV system has been predominantly applied in combination with AT-MSC for treating neural tumors demonstrating very high efficacy [238, 239, 280]. Furthermore, in a phase I/II clinical trial started in 2015, the very first one worldwide to evaluate the safety and efficacy of GM-BM-MSC against advanced, recurrent, or metastatic gastrointestinal or hepatopancreatobiliary adenocarcinoma, the trial methodology was based on intravenous injection of HSV-tk-engineered MSC in patients, followed by repeated GCV injections [281].

It is worth highlighting that in some cases, in an effort to maximize tumor suppression efficiency, a *combination of strategies* is employed as evidenced for example in the case of UC-MSC-IFN-β plus 5-FU (cytokine-transduced MSC + chemotherapy drug) for the treatment of breast cancer [73], UC-MSC-sTRAIL plus 5-FU (apoptosis agent-transduced MSC + chemotherapy drug) [252], BM-MSC transduced with sTRAIL plus HSVtk/GCV (apoptosis agent-transduced MSC + GDEPT system) for glioblastoma treatment [231], and HSVtk/GCV in combination with CD::UPRT/5-FC (double GDEPT) used on AT-MSC against glioblastoma [239] or breast-to-lung metastatic cancer [273]. These approaches result in various degrees of synergy, ranging from mild, non-significant enhancement of tumor suppression, to additive or stronger synergistic effects depending on the tested cell line and experimental setup. For example, Matuskova and colleagues showed that sequential treatment (single treatment with CD/5-FC followed by HSVtk/GCV) leads to synergic cooperation of CD and HSVtk against MDA-MB-231 metastases to lung, an effect that is stronger at higher GCV doses [273].

Obviously, the multitude of combinations of antitumorigenic molecular cargo, cellular vehicle, tumor model, gene incorporation, and expression technology and method of administration enables the generation of a large number of individual GM-MSC-based anti-cancer strategies laying the basis for personalized cancer cytotherapy. Interestingly, a comparison of the different strategies applied in relation to a specific cancer model frequently used in experimental cytotherapy studies reveals substantial accordance in the results obtained. For example, with respect to the popular xenograft lung metastasis model, administration of human GM-MSC in mice suppressed the metastases of human MBA-MB-231 breast cells to the lung (determined by measuring the increase in median survival of GM-MSC-treated mice) by 62%, 81%, and 73% (72 ± 9.5%) for BM-MSC-IFN-β [33], BM-MSC-TRAIL [226], or AT-MSC receiving double GDEPT (CD::UPRT/5-FC : HSVtk/GCV, 1:1) [273], respectively. Similarly, metastases of MDA-MB-231 breast cells were reduced by 53%, 42%, and 39% (45 ± 7.4%) in mice receiving BM-MSC-IFN-β [33], UC-MSC-IFN-β [72], or UC-MSC-IFN-β+5-FU combination treatment [73], respectively, as evidenced by the reduction of mouse lung masses one month after GM-MSC administration.

Regardless of the strategy followed, successfully marrying high efficacy with safety is the primary challenge in developing a *gene delivery system*. So far all GM efforts have relied on the heavy use of viral vectors (adenovirus—AV, retrovirus—RV, or lentivirus—LV). Generally, viral vectors are more efficient at transferring genes than non-viral vectors; however, clinical studies have revealed that the efficacy of virus-mediated suicide gene therapy was limited due to adverse effects, such as toxicity, oncogenicity, and the possibility of invoking an excessive antiviral host immune response [282]. For example, AV expression is transient and often produces a significant host immune response, whereas RV may cause incorporation errors or even oncogene activation [283, 284]. LV vectors are less likely to cause insertional mutagenesis, since the promoter can be modified extensively. Furthermore, they have the ability to stably transduce both dividing and quiescent cells, which is a further significant advantage when using stem cells that are often quiescent or slow growing [285]. According to our analysis, LV is more frequently associated with MSC transduced with TRAIL (50% adoption rate compared to other antitumor agents). In this setting, LV has been shown to efficiently and stably transduce MSC without affecting their stemness, while at the same time, LV modification does not result in altering DNA copy number and arrangement in MSC [286]. Lately, advances in genetic engineering methods have led to the development of innovative non-viral nano-vectors that combine the low toxicity and immunogenicity of cationic liposomic carriers with the high transfection efficiency of viral systems [287, 288].

#### Naïve MSC vs. GM-MSC-based cytotherapy efficacy

**Fig. 6:**
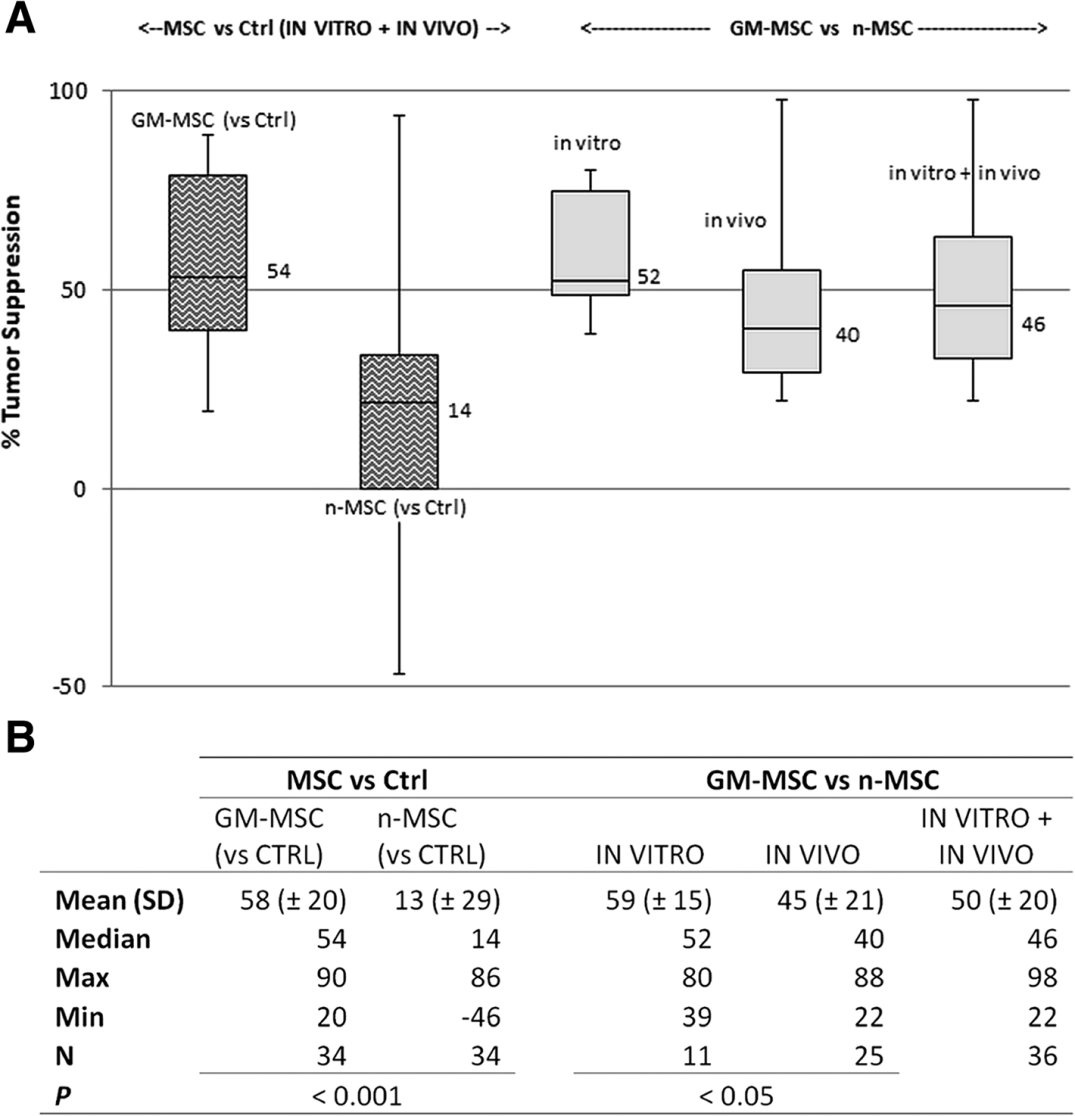
Tumor suppression rates (%) for genetically modified MSC (GM-MSC) in comparison to naïve MSC (n-MSC) and non-MSC controls. Tumor suppression rates were determined using measured experimental outcomes (e.g., tumor size and weight, animal survival rate, proliferation assays) derived from 20 selected publications in which the anti-cancer efficacy of genetically modified human MSC was directly compared to that of unmodified, naïve MSC and non-MSC controls. **a** Whisker plots. Values next to whisker boxes depict median values. **b** Tabular summary of the data plotted in **a**. Sample size (*N*) relates to total number of experimental readouts (e.g., tumor size and weight, animal survival rate, proliferation assays) based on which % tumor suppression was calculated. *P* values correspond to *t* tests comparing the specific groups

As mentioned earlier, GM-MSC-based cancer cytotherapy has shown greater consistency in successfully suppressing tumorigenesis. Nevertheless, the difference in tumor suppression efficiency in comparison to n-MSC has been largely discussed on a qualitative, rather than a quantitative basis. Our meta-analysis approach gives for the first time, to the best of our knowledge, a quantitative dimension to the advantage of GM-MSC over n-MSC-based cancer cytotherapy. With respect to the studies using GM-MSC, they all report attenuation of tumor growth, irrespective of the type of MSC effector, tumor target, GM strategy, or animal/culture model applied. On the contrary, as previously discussed, the frequency of n-MSC studies showing a significant onco-suppressive effect varies greatly (26–88%, mean = 48%) depending on the type of MSC employed (Fig. 3c). In other words, n-MSC-based cancer cytotherapy is more “hit-or-miss” in terms of reliability with the final outcome largely dependent on the MSC-tumor combination. In an effort to verify this observation and to provide a more accurate quantitative estimate of the impact of GM-MSC over n-MSC, we focused our meta-analysis on experimental data from selected studies referring to experimental cytotherapy work on human lung, breast and brain cancers, in which human GM-MSC (of BM, AT, or UC origin) had been directly compared to n-MSC as well as to matched non-MSC controls within the same experimental setup [72, 73, 128, 200, 226, 227, 229, 232–237, 241, 242, 273, 289]. Such experimental readouts relating to anti-cancer efficacy included measurement of size and/or weight of the developing tumors, as well as changes in animal survival rates (in vivo), and determination of cell proliferation and/ or viability (in vitro). The wider range of antitumor effects of n-MSC is largely attributed to BM-MSC. For example, intratumoral (intracranial) or systemic (intracarotid) administration of naïve BM-MSC in athymic mice with established gliomas resulted in reduction in median survival of the mice by 6 and 5 days (or by 20% and 16%), respectively, as compared to controls. On the contrary, infusion of BM-MSC-IFN-β via the same routes extended animal survival by 12 and 8 days (or by 29% and 20%), respectively, over the U87-only control group [236]. Overall, according to our analysis the tumor suppression efficiency of GM-MSC is higher by about 40% compared to that of n-MSC, as depicted by the significant difference in mean and in median values of the two groups (Fig. 6). A point worth highlighting is also the fact that in vitro experimentation tends to generate tumor suppression rates (GM-MSC/n-MSC) that are generally higher than those obtained using rodent tumor models; this is particularly evident in the case of cancer cell apoptosis evaluation in vitro, where GM-MSC show on average an eightfold higher pro-apoptotic effect, compared to their unmodified counterparts (data not shown). Nevertheless, the therapeutic advantage of GM-MSC in vivo is strong, with the latter extending, for example regarding median survival time of tumor-bearing mice by an average of 16 days over n-MSC, equivalent to a life extension by two to four years in humans (data not shown).

Taking all the above into consideration, it can be deduced that GM-MSC exhibit greater antitumor efficiency when used as delivery vehicles in comparison with non-modified cells. For GM-BM-MSC, there is enough preclinical evidence showing the efficacy of IFN-β on solid tumor suppression to warrant the move to clinical trials. Indeed, by mid-2019 a trial involving up to 21 patients with advanced ovarian cancer is expected to provide the first ever data on the maximum tolerability and safety of MSC-IFN-β in humans (NCT02530047; [262]). With respect to GM-AT-MSC, experimental cytotherapy data so far are suggestive of an increased antitumor efficacy in relation to gliomas, especially when combined with a form of GDEPT. Last but not least, in the case of UC-MSC, genetic modification can only enhance their intrinsic suppressive action on tumor growth. Unfortunately, large gaps in GM-UC-MSC work do not allow us to have a more complete picture regarding their anti-cancer performance against important cancers such as colorectal and prostate. Nevertheless, preclinical data so far suggest that breast and lung cancers comprise two potential targets for GM-UC-MSC testing in humans. The hypo-immunogenicity and non-tumorigenicity of human UC-MSC are additional features acting in support of the adoption of the specific cellular vehicle, in an allogeneic setting, for the transition from the bench to the bedside.

## Conclusions

In our effort to decode the diversity of MSC-based cancer cytotherapy outcomes, we methodically recorded and analyzed experimental data reported in the relevant literature. Our approach, although carefully designed, is far from perfect as it is based on a relatively limited number of observations that restrict the statistical resolution to the descriptive statistics level. Nevertheless, the trends obtained are representative and agree with the literature. In summary, our findings allow the following conclusions to be drawn: (a) more studies focusing on frequently occurring tumors are needed. This holds truth for both naïve and GM-MSC-based research work. For example, experimental data on naïve MSC targeting cancers such as hepatic, gastric and lung are rare, rendering any attempt to evaluate the MSC performance against the latter unsuccessful. Prioritizing these targets will provide more information towards a deeper understanding of the biology of MSC: tumor interactions, at the same time setting a solid basis for the development of more efficient MSC engineering protocols. Special emphasis should also be given on cancers for which there are preliminary studies with encouraging data that need confirmation, or there are no studies evaluating the anti-cancer performance of specific MSC types. These are pancreatic (naïve AT-/BM-MSC), colon and prostate (naïve and GM-UC-MSC), and brain cancers (GM-UC-MSC). (b) More attention should be paid to experimental design and implementation to minimize the technical heterogeneity, which comprises a major contributor for inconsistencies and erratic outcomes observed in experimental tumor cytotherapy. Experimental parameters such as the use of multiple cell lines to represent each tumor target, the MSC to cancer cell ratio, timing and mode of cell administration used, and, last but not least, the type of animal model adopted are of particular importance. (c) Genetic modification of MSC offers a significant advantage in anti-cancer efficacy over naïve MSC, successfully alleviating the bimodal effects of some MSC populations against certain tumors shifting the balance robustly toward tumor suppression and can become the new standard once the enabling technologies become safer. More focused clinical trials are needed towards this end. (d) Despite the omissions and inconsistencies, a critical mass of data from available studies so far suggest that the primary outcome of experimental cytotherapy is largely dictated by the biology of the interaction of the specific effector MSC with its target, with some MSC populations (e.g., UC-MSC) generating a strong and robust tumoricidal response against specific tumors (e.g., breast), while others (BM-MSC) are clearly unsuitable for use on the same target, since they display pro-tumorigenic behavior (Tables 5 and 6). Differences in antitumor efficacy of MSC are determined by the dynamics of their interactions with tumor niche components, which in turn are determined by their phenotypic and functional traits (e.g., secretome profile, senescence status, cell surface receptor expression levels), and ultimately depend on the developmental origin of these cells. (e) While a single stem cell type with a ubiquitous anti-cancer behavioral profile is unlikely to exist, our analysis indicates that MSC derived from the umbilical cord of the human fetus possess enhanced antitumorigenic characteristics compared to adult MSC, which are largely likely attributed to their unique immunomodulatory profile as primitive stem cells.

**Table 5:** Summary of evidence-based cancer therapy by MSC as deduced by the meta-analysis of peer-reviewed experimental/ preclinical research studies

MSC	MSC GM status #	Tumor target	Effect on tumor target	Evidence in support
AT	Modified (or naïve)	Neural	Suppression	**
UC	Naïve (or modified)	Breast	Suppression	***
UC	Modified (or naïve)	Lung	Suppression	**
BM	Modified	Neural	Suppression	**
BM	Modified	Lung	Suppression	**
BM	Modified (or naïve)	Pancreas	Suppression	*
BM	Naïve	Breast	Promotion	***
BM	Naïve	Osteosarcoma	Promotion	*

# Primary or secondary/minor (in brackets) contribution of the genetic modification status of the MSC to the overall observed effect on tumor target

No. of studies *≥ 3, **≥ 6, ***≥ 10

**Table 6:** Summary of MSC suitability for experimental cancer cytotherapy applications

				MSC suitability for cytotherapy		
				Most favorable attributes Best option ✓	Some favorable attributes Alternative use (✓)	Detrimental effects questionable/contraindicated ?
				(+ 1)	(+ 0.5)	(− 0.5)
Cytotherapy evaluation areas [links to supportive evidence in the review]	Adequacy	1	MSC donor tissue abundance/availability Ease of isolation/derivation Harvest yield [4.1.3, 4.1.4]	UC AT	BM	
		2	Ease of sub-culture manipulation Expansion/upscaling potential [4.1.4]	UC	AT GM-BM	
	Safety	3	Genomic stability [4.2]	UC	AT	BM GM-MSC
		4	Host integration/immunogenicity [4.2, 4.3.1, Table 4]	UC	GM-AT	
	Efficacy	5	Robust target-specific anti-cancer action [Table 5, Additional file 6: Figure S6]	UC (vs. breast) GM-BM (vs. lung) GM-BM (vs. neural)	GM-UC (vs. breast) GM-UC (vs. lung) GM-AT (vs. neural)	BM (vs. breast)
		6	% frequency anti- vs. pro-tumorigenic events [Table 1, Figs. 3, 4, 5b, and 6]	UC (breast, lung) GM-MSC	AT (neural)	BM (breast, colorectal
	MSC total scores			UC: 6 * (+1) = + 6 GM-BM: 2 * (+1) = + 2 AT: 1 * (+1) = + 1 GM-MSC: 1 * (+1) = + 1	AT: 3 * (+0.5) = + 1.5 BM: 1 * (+0.5) = + 0.5 GM-AT: 2 * (+0.5) = + 1 GM-UC: 2 * (+0.5) = + 1	BM: 3 * (-0.5) = - 1.5 GM-MSC 1 * (-0.5) = - 0.5
	Ranking:					
	Tissue-specific			Cord (+ 7) > fat (+ 3.5) > marrow (+ 1)		
	Fetal vs. adult			Fetal (+ 7) > adult (+ 4.5)		
	Naïve vs. GM			Naïve (+ 7.5) > GM (+ 4.5)		

MSC, both naïve (UC/AT/BM), as well as genetically modified (GM-UC, GM-BM, GM-AT) were evaluated using an arbitrary scoring system based on the possession of favorable (positive scoring) or detrimental (negative scoring) characteristics with respect to six main cytotherapy evaluation areas pertaining to adequacy, safety and efficacy. The evaluation points/areas on which MSC are judged are listed on the left hand–side columns; the links in brackets provide the relative evidence presented throughout the review in support of the allocation of scores which are given to MSC according to the conformity of their traits to the specific cytotherapy requirements. This grading of the suitability of MSC across various aspects of cytotherapy results in the build of a matrix (shadowed area of the table) in which each of the MSC entries in the first, second, and third columns are valued as + 1, + 0.5, and − 0.5, respectively. A score of + 1 is assigned to the MSC that possess the most favorable attributes (e.g., highest isolation yield, greatest expansion potential) and which are classified as an ideal cell choice. The MSC that gather fewer or to a lesser extent favorable characteristics, are regarded as a second, alternative MSC option and score + 0.5, while those associated with detrimental effects (e.g., mutation events, tumor promotion) get a negative score (− 0.5) and are considered of questionable value or contraindicated, for certain cytotherapy applications at least. Final ranking of the MSC types is done by calculating the total sum of points for each of them within the matrix and then listing them in descending order, i.e., from highest suitability score to lowest (bottom row of table, scores in brackets). Note that ranking is only indicative of the relative suitability of MSC for cancer treatment based on experimental work hitherto and does not take into account the relative misrepresentation of some MSC types (e.g., under-representation of UC-GM-MSC against colorectal, prostate, and neural tumors)

At this stage, any attempt to give a detailed account of the mediating mechanisms would be largely speculative, given the lack of studies focusing on the in vivo immunomodulatory effects of administered MSC within tumors, and the inadequacy of the present experimental models to recapitulate the multitude of highly plastic and bidirectional interactions that take place. Nevertheless, data patterns emerging from this meta-analysis (as discussed in the “Exploring the role of experimental parameters on MSC-based cancer cytotherapy outcome” and “Linking MSC phenotypic differences to variation in anti-cancer efficacy” sections) interpreted in the context of co-culture experiments examining MSC-immune effector cell interactions or focusing on the interplay between various tumor stroma cell classes during carcinogenesis can be summarized into a conceptual framework as follows: (i) Different types of exogenous MSC exert variable, often opposing, effects on the same types of tumor targets in vitro and in vivo. (ii) The tumor-promoting/-suppressive effect of MSC may involve direct contact with the components of the tumor microenvironment or may rely on paracrine/juxtacrine effects mediated by the secreted form of induced molecules. (iii) The type and extent of immune response regulation might be a function of the maturity and composition of the microenvironment as well as the differential sensitivity of various MSC types to these maturation levels. (iv) In any case, in an effort to fulfil their primary role of maintaining tissue homeostasis, MSC interfere with innate and immune response both in vitro and in vivo ultimately adopting a pro- or anti-inflammatory phenotype [290]. (v) The inflammatory response shares various molecular targets and signaling pathways with the carcinogenic process, such as increased cell proliferation rate, apoptosis, and angiogenesis. MSC are able to influence such cancer hallmarks by directly adopting a polarized inflammatory phenotype themselves or by directly polarizing infiltrating immune cells, most notably macrophages, into M1 (proinflammatory) or M2 (anti-inflammatory/immunosuppressive) types. (vi) Exogenous adult MSC exhibit a propensity towards adoption of the proinflammatory phenotype under the dictation of mutationally corrupted cancer cells emitting signals (secreted chemokines) deceivingly associated with epithelial injury and infection. Once recruited into the tumor microenvironment BM-MSC and AT-MSC are induced to differentiate towards stromal TAF/CAF (activated myofibroblasts or adipocytes, respectively) that further propagate tumor development by providing mitogenic epithelial growth factors directly to cancer cells, by facilitating EMT or further recruitment of immune cells ([291], references within). Adult MSC may be more susceptible to transformation into TAF due to their genomic instability (accumulated oxidative damage) and elevated senescence status (shorter telomeres). (vii) In vivo, fetal UC-MSC exhibit a more robust anti-cancer behavior by sustaining a healthy microenvironment by actively skewing immune responses to an anti-inflammatory profile (involving TRL3—rather than TLR4—ligation, emergence of T-reg and activation of immune complexes), resembling events occurring during tissue regeneration and resolution of inflammation. (viii) In vitro data showing the strong anti-proliferative effect of culture media containing secreted UC-MSC metabolic products (secretome) against cancer cells suggest that UC-MSC constitutively produce trophic factors that possess intrinsic antitumor properties. The secretome alone can sufficiently mount a strong dose- and time-dependent anti-proliferative response against certain tumor cells mediated by apoptosis signaling (Christodoulou and Goulielmaki, manuscript in preparation). (ix) The unique antitumorigenic properties of UC-MSC most likely are attributed to a key combination of secreted molecules normally associated with the structural and functional traits of the host organ tissue and its specialized role in the feto-maternal interface (e.g., LIF/IL-6: stemness; HLA-G: immune tolerance; glycocalyx matrix hyaluronan; structural integrity)**.**

Overall, the marked anti-cancer efficacy of UC-MSC, in addition to their “off-the-shelf” availability, which comprises the relatively greater abundance (ease of isolation, enhanced expansion potential) and safety (reduced risk of malignant transformation and hypo-immunogenic profile which allows them to be used in an allogeneic setup), compared to their adult counterparts highlights UC-MSC as instrumental tools in cancer cytotherapy (Table 6). Clearly, the outcome of clinical trials will be crucial before these promising cells find their way into the clinic. Nevertheless, after almost two decades of experimentation, it seems that the cancer cytotherapy field is slowly coming off age.

## Additional files

Additional file 1: **Figure S1.** Comparison of research activity (peer-reviewed journal articles) on selected cancer treatment options over the last two decades. The four graphs (solid lines) depict the evolution of publication rates of journal articles reporting research work in PubMed on four types of therapies relative to cancer treatment (radiotherapy, combination chemotherapy, immunotherapy, and MSC-based cytotherapy) over a period of 20 years. Graphs are based on reported rates over seven 3-year periods, with a forward projection of one period (dashed trend line). In all four cases, the rates increase exponentially (*y* = *a e*^*b x*^); nevertheless, the growth for cancer cytotherapy publications (purple) occurs at a relatively higher rate as shown by the steeper trend line and higher value of *b* constant in the respective equation. This publication rate for MSC-based cancer cytotherapy studies has grown over sixfold faster, compared to that for combined chemotherapy, for example, rising from 1:100 to 1:15 over the last decade. This trend is suggestive of increased interest in this field that will most likely be sustained in the near future. PubMed search filters: English only, research articles only. (TIF 30030 kb)

Additional file 2: **Figure S2.** Overview of meta-analysis methodology (TIF 12282 kb)

Additional file 3: **Figure S3.** Example of a database form used to record experimental data used in the meta-analysis. Field titles correspond to the parameters comprising each of the in vitro and in vivo experiments as described in the methodology and results sections of the relevant articles. (TIF 9196 kb)

Additional file 4: **Figure S4.** Distribution of the three most frequently associated tumors in relation to MSC effectors. Sample sizes: adipose-derived MSC (AT-MSC) = 32, bone marrow-derived MSC (BM-MSC) = 56, umbilical cord-derived MSC (UC-MSC) = 34. (TIF 4256 kb)

Additional file 5: **Figure S5.** Comparison of distribution of anti-cancer effects for naïve MSC vs. naïve MSC used as control cells for genetically modified MSC-based cancer cytotherapy studies (Naïve + GM). Each of the 100% stacked columns shows the relative distribution of anti-cancer effect observed (anti- vs. pro-tumorigenic vs. neutral) (TIF 103676 kb)

Additional file 6: **Figure S6.** List and frequency distribution of studies employing the use of genetically modified stem cells (GM-MSC) of human adipose tissue (AT), bone marrow (BM), and fetal umbilical cord (UC) matrix origin. In each row of the table, the length of black-gradient filled horizontal bars is proportional to the total number of studies (value within bar) relevant to specific GM-MSC/tumor combinations; the list of respective citations is shown under the bars. Cancer types are ranked in descending order of world incidence (see also Fig. 2). Only tumors whose use is described by three or more independent studies are shown. Arrows at the beginning of each row of the table symbolize deviation of the frequency of tumor targeted in experimental cytotherapy work from their respective incidence/frequency of occurrence globally (yellow = difference within 5%; green, up = difference > 5% in favor of cytotherapy—tumor over-representation; red, down = difference of > 5% in favor of incidence—tumor under-representation). */**/# Studies referring to cervical cancer/ ovarian cancer/ use of UC-blood MSC, respectively. (TIF 9450 kb)

## Abbreviations

5-FC: 5-Fluorocytosine
AT-MSC: Adipose tissue-derived mesenchymal stem cells
BM-MSC: Bone marrow stroma-derived mesenchymal stem cells
CD::UPRT: Cytosine deaminase to uracil phosphoribosyltransferase fusion protein
CFU-F: Colony-forming unit–fibroblast
CM: Conditioned media
ECM: Extracellular matrix
EMT: Epithelial-to-mesenchymal transition
GDEPT: Gene-directed enzyme/prodrug treatment
GM-MSC: Genetically modified mesenchymal stem cells
GSV: Ganciclovir
HLA: Human leukocyte antigen
HSV-tk: Herpes simplex virus thymidine kinase
i.v.: Intravenous
IFN: Interferon
MHC: Major histocompatibility complex
MLR: Mixed lymphocyte reaction
MNC: Mononuclear cells
MSC: Mesenchymal stem cells
n-MSC: Naïve (unmodified) mesenchymal stem cells
PD: Population doublings
PDT: Population doubling time
s.c.: Subcutaneous
SCID: Severe combined immunodeficiency
TAF: Tumor-associated fibroblasts
TLR: Toll-like receptor
TRAIL: Tumor necrosis factor-related apoptosis-inducing ligand
UC-MSC: Umbilical cord matrix-derived mesenchymal stem cells
WJ: Wharton’s jelly

## References

[1] Sage EK, Thakrar RM, Janes SM (2016). Genetically modified mesenchymal stromal cells in cancer therapy. Cytotherapy.

[2] Balkwill FR, Capasso M, Hagemann T (2012). The tumor microenvironment at a glance. J Cell Sci.

[3] Rowley DR (1998). Cancer Metastasis Rev.

[4] Hasebe T, Sasaki S, Sugitoh M, Ono M, Saitoh N, Ochiai A (2001). Highly proliferative intratumoral fibroblasts and a high proliferative microvessel index are significant predictors of tumor metastasis in T3 ulcerative-type colorectal cancer. Hum Pathol.

[5] Fridman WH, Pagès F, Sautès-Fridman C, Galon J (2012). The immune contexture in human tumors: impact on clinical outcome. Nat Rev Cancer.

[6] Milne K, Köbel M, Kalloger SE, Barnes RO, Gao D, Gilks CB, Watson PH, Nelson BH (2009). Systematic analysis of immune infiltrates in high-grade serous ovarian cancer reveals CD20, FoxP3 and TIA-1 as positive prognostic factors. PLoS One.

[7] Qin Z, Richter G, Schüler T, Ibe S, Cao X, Blankenstein T (1998). B cells inhibit induction of T cell-dependent tumor immunity. Nat Med.

[8] Condeelis J, Pollard JW (2006). Macrophages: obligate partners for tumor cell migration, invasion, and metastasis. Cell.

[9] Nozawa H, Chiu C, Hanahan D (2006). Infiltrating neutrophils mediate the initial angiogenic switch in a mouse model of multistage carcinogenesis. Proc Natl Acad Sci.

[10] Youn J-I, Gabrilovich DI (2010). The biology of myeloid-derived suppressor cells: the blessing and the curse of morphological and functional heterogeneity. Eur J Immunol.

[11] Armulik A, Genové G, Betsholtz C (2011). Pericytes: developmental, physiological, and pathological perspectives, problems, and promises. Dev Cell.

[12] Orimo A, Gupta PB, Sgroi DC, Arenzana-Seisdedos F, Delaunay T, Naeem R, Carey VJ, Richardson AL, Weinberg RA (2005). Stromal fibroblasts present in invasive human breast carcinomas promote tumor growth and angiogenesis through elevated SDF-1/CXCL12 secretion. Cell.

[13] Radisky ES (2015). Matrix metalloproteinases as drivers and therapeutic targets in breast cancer. Front Biosci.

[14] Kurose K (2001). Genetic model of multi-step breast carcinogenesis involving the epithelium and stroma: clues to tumor-microenvironment interactions. Hum Mol Genet.

[15] Moinfar F, Man YG, Bratthauer GL, Ratschek M, Tavassoli FA (2000). Genetic abnormalities in mammary ductal intraepithelial neoplasia-flat type (“clinging ductal carcinoma in situ”). Cancer.

[16] Djouad F, Plence P, Bony C, Tropel P, Apparailly F, Sany J, Noel D, Jorgensen C (2003). Immunosuppressive effect of mesenchymal stem cells favors tumor growth in allogeneic animals. Blood.

[17] Hill R, Song Y, Cardiff RD, Van Dyke T (2005). Selective evolution of stromal mesenchyme with p53 loss in response to epithelial tumorigenesis. Cell.

[18] McCullough KD, Coleman WB, Ricketts SL, Wilson JW, Smith GJ, Grisham JW (1998). Plasticity of the neoplastic phenotype in vivo is regulated by epigenetic factors. Proc Natl Acad Sci.

[19] Provenzano PP, Inman DR, Eliceiri KW, Knittel JG, Yan L, Rueden CT, White JG, Keely PJ (2008). Collagen density promotes mammary tumor initiation and progression. BMC Med.

[20] Siegel PM, Massagué J (2003). Cytostatic and apoptotic actions of TGF-β in homeostasis and cancer. Nat Rev Cancer.

[21] Barr S, Thomson S, Buck E, Russo S, Petti F, Sujka-Kwok I, Eyzaguirre A, Rosenfeld-Franklin M, Gibson NW, Miglarese M, Epstein D, Iwata KK, Haley JD (2008). Bypassing cellular EGF receptor dependence through epithelial-to-mesenchymal-like transitions. Clin Exp Metastasis.

[22] Perou CM, Sørlie T, Eisen MB, van de Rijn M, Jeffrey SS, Rees CA, Pollack JR, Ross DT, Johnsen H, Akslen LA, Fluge Ø, Pergamenschikov A, Williams C (2000). Nature.

[23] Weissman IL, Anderson DJ, Gage F (2001). Stem and progenitor cells: origins, phenotypes, lineage commitments, and transdifferentiations. Annu Rev Cell Dev Biol.

[24] Ulloa-Montoya F, Verfaillie CM, Hu W-S (2005). Culture systems for pluripotent stem cells. J Biosci Bioeng.

[25] Pittenger MF (1999). Multilineage potential of adult human mesenchymal stem cells. Science.

[26] Dominici M, Le Blanc K, Mueller I, Slaper-Cortenbach I, Marini FC, Krause DS, Deans RJ, Keating A, Prockop DJ, Horwitz EM (2006). Minimal criteria for defining multipotent mesenchymal stromal cells. The International Society for Cellular Therapy position statement. Cytotherapy.

[27] Shih YRV, Kuo TK, Yang AH, Lee OK, Lee CH (2009). Isolation and characterization of stem cells from the human parathyroid gland. Cell Prolif.

[28] Rochefort GY, Delorme B, Lopez A, Hérault O, Bonnet P, Charbord P, Eder V, Domenech J (2006). Multipotential mesenchymal stem cells are mobilized into peripheral blood by hypoxia. Stem Cells.

[29] Flynn A, Barry F, O'Brien T (2007). UC blood-derived mesenchymal stromal cells: an overview. Cytotherapy.

[30] Ulicna M, Danisovic L, Vojtassak J (2010). Does cell therapy and tissue engineering represent a promising treatment of diabetic foot ulcers?. Bratisl Lek Listy.

[31] Xi J, Yan X, Zhou J, Yue W, Pei X (2013). Mesenchymal stem cells in tissue repairing and regeneration: progress and future. Burns Trauma.

[32] Spaeth E, Klopp A, Dembinski J, Andreeff M, Marini F (2008). Inflammation and tumor microenvironments: defining the migratory itinerary of mesenchymal stem cells. Gene Ther.

[33] Studeny M, Marini FC, Dembinski JL, Zompetta C, Cabreira-Hansen M, Bekele BN, Champlin RE, Andreeff M (2004). Mesenchymal stem cells: potential precursors for tumor stroma and targeted-delivery vehicles for anticancer agents. J Natl Cancer Inst.

[34] Ramasamy R, Lam EW, Soeiro I, Tisato V, Bonnet D, Dazzi F (2007). Mesenchymal stem cells inhibit proliferation and apoptosis of tumor cells: impact on in vivo tumor growth. Leukemia.

[35] Spaeth EL, Dembinski JL, Sasser AK, Watson K, Klopp A, Hall B, Andreeff M, Marini F (2009). Mesenchymal stem cell transition to tumor-associated fibroblasts contributes to fibrovascular network expansion and tumor progression. PLoS One.

[36] Dvorak HF (1986). Tumors: wounds that do not heal. Similarities between tumor stroma generation and wound healing. N Engl J Med.

[37] Wu X, Hu J, Zhou L, Mao Y, Yang B, Gao L, Xie R, Xu F, Zhang D, Liu J, Zhu J (2008). In vivo tracking of superparamagnetic iron oxide nanoparticle–labeled mesenchymal stem cell tropism to malignant gliomas using magnetic resonance imaging. J Neurosurg.

[38] Kidd S, Spaeth E, Dembinski JL, Dietrich M, Watson K, Klopp A, Battula VL, Weil M, Andreeff M, Marini FC (2009). Direct evidence of mesenchymal stem cell tropism for tumor and wounding microenvironments using in vivo bioluminescent imaging. Stem Cells.

[39] Dwyer RM, Potter-Beirne SM, Harrington KA, Lowery AJ, Hennessy E, Murphy JM, Barry FP, O'Brien T, Kerin MJ (2007). Monocyte chemotactic protein-1 secreted by primary breast tumors stimulates migration of mesenchymal stem cells. Clin Cancer Res.

[40] Fedyk ER, Jones D, Critchley HOD, Phipps RP, Blieden TM, Springer TA (2001). Expression of stromal-derived factor-1 is decreased by IL-1 and TNF and in dermal wound healing. The Journal of Immunology.

[41] Menon LG, Picinich S, Koneru R, Gao H, Lin SY, Koneru M, Mayer-Kuckuk P, Glod J, Banerjee D (2007). Differential gene expression associated with migration of mesenchymal stem cells to conditioned medium from tumor cells or bone marrow cells. Stem Cells.

[42] Birnbaum T, Roider J, Schankin CJ, Padovan CS, Schichor C, Goldbrunner R, Straube A (2007). Malignant gliomas actively recruit bone marrow stromal cells by secreting angiogenic cytokines. J Neurooncol.

[43] Ho IAW, Chan KYW, Ng W-H, Guo CM, Hui KM, Cheang P, Lam PYP (2009). Matrix metalloproteinase 1 is necessary for the migration of human bone marrow-derived mesenchymal stem cells toward human glioma. Stem Cells.

[44] De Ugarte DA, Alfonso Z, Zuk PA, Elbarbary A, Zhu M, Ashjian P, Benhaim P, Hedrick MH, Fraser JK (2003). Differential expression of stem cell mobilization-associated molecules on multi-lineage cells from adipose tissue and bone marrow. Immunol Lett.

[45] Jacobsen K, Kravitz J, Kincade PW, Osmond DG (1996). Adhesion receptors on bone marrow stromal cells: in vivo expression of vascular cell adhesion molecule-1 by reticular cells and sinusoidal endothelium in normal and gamma-irradiated mice. Blood.

[46] Ruster B, Gottig S, Ludwig RJ, Bistrian R, Muller S, Seifried E, Gille J, Henschler R (2006). Mesenchymal stem cells display coordinated rolling and adhesion behavior on endothelial cells. Blood.

[47] Valenick LV, Hsia HC, Schwarzbauer JE (2005). Fibronectin fragmentation promotes α4β1 integrin-mediated contraction of a fibrin–fibronectin provisional matrix. Exp Cell Res.

[48] Direkze NC, Hodivala-Dilke K, Jeffery R, Hunt T, Poulsom R, Oukrif D, Alison MR, Wright NA (2004). Bone marrow contribution to tumor-associated myofibroblasts and fibroblasts. Cancer Res.

[49] Karnoub AE, Dash AB, Vo AP, Sullivan A, Brooks MW, Bell GW, Richardson AL, Polyak K, Tubo R, Weinberg RA (2007). Mesenchymal stem cells within tumor stroma promote breast cancer metastasis. Nature.

[50] Yang Y, Bucan V, Baehre H, Von Der Ohe J, Otte A, Hass R (2015). Acquisition of new tumor cell properties by MSC-derived exosomes. Int J Oncol.

[51] Vallabhaneni KC, Penfornis P, Dhule S, Guillonneau F, Adams KV, Mo YY, Xu R, Liu Y, Watabe K, Vemuri MC, Pochampally R (2015). Extracellular vesicles from bone marrow mesenchymal stem/stromal cells transport tumor regulatory microRNA, proteins, and metabolites. Oncotarget.

[52] Chowdhury R, Webber JP, Gurney M, Mason MD, Tabi Z, Clayton A (2015). Cancer exosomes trigger mesenchymal stem cell differentiation into pro-angiogenic and pro-invasive myofibroblasts. Oncotarget.

[53] Presta LG, Chen H, O'Connor SJ, Chisholm V, Meng YG, Krummen L, Winkler M, Ferrara N (1997). Humanization of an anti-vascular endothelial growth factor monoclonal antibody for the therapy of solid tumors and other disorders. Cancer Res.

[54] Zolochevska O, Yu G, Gimble JM, Figueiredo ML (2012). Pigment epithelial-derived factor and melanoma differentiation associated gene-7 cytokine gene therapies delivered by adipose-derived stromal/mesenchymal stem cells are effective in reducing prostate cancer cell growth. Stem Cells Dev.

[55] Ryu CH, Park S-H, Park SA, Kim SM, Lim JY, Jeong CH, Yoon W-S, W-i O, Sung YC, Jeun S-S (2011). Gene therapy of intracranial glioma using interleukin 12–secreting human umbilical cord blood–derived mesenchymal stem cells. Hum Gene Ther.

[56] Hombauer H, Minguell JJ (2000). Selective interactions between epithelial tumor cells and bone marrow mesenchymal stem cells. Br J Cancer.

[57] Larmonier N, Ghiringhelli F, Larmonier CB, Moutet M, Fromentin A, Baulot E, Solary E, Bonnotte B, Martin F (2003). Freshly isolated bone marrow cells induce death of various carcinoma cell lines. Int J Cancer.

[58] Fierro FA, Sierralta WD, Epuñan MJ, Minguell JJ (2004). Marrow-derived mesenchymal stem cells: role in epithelial tumor cell determination. Clin Exp Metastasis.

[59] Khakoo AY, Pati S, Anderson SA, Reid W, Elshal MF, Rovira II, Nguyen AT, Malide D, Combs CA, Hall G, Zhang J, Raffeld M, Rogers TB (2006). Human mesenchymal stem cells exert potent antitumorigenic effects in a model of Kaposi’s sarcoma. J Exp Med.

[60] Wu X-B, Liu Y, Wang G-H, Xu X, Cai Y, Wang H-Y, Li Y-Q, Meng H-F, Dai F, Jin J-D (2016). Mesenchymal stem cells promote colorectal cancer progression through AMPK/mTOR-mediated NF-κB activation. Sci Rep.

[61] Zhu W, Xu W, Jiang R, Qian H, Chen M, Hu J, Cao W, Han C, Chen Y (2006). Mesenchymal stem cells derived from bone marrow favor tumor cell growth in vivo. Exp Mol Pathol.

[62] Kucerova L, Matuskova M, Hlubinova K, Altanerova V, Altaner C (2010). Tumor cell behaviour modulation by mesenchymal stromal cells. Mol Cancer.

[63] Yu JM, Jun ES, Bae YC, Jung JS (2008). Mesenchymal stem cells derived from human adipose tissues favor tumor cell growthin vivo. Stem Cells Dev.

[64] Akimoto K, Kimura K, Nagano M, Takano S, Yamashita T, Ohneda O, To'a Salazar G (2013). Umbilical cord blood-derived mesenchymal stem cells inhibit, but adipose tissue-derived mesenchymal stem cells promote, glioblastoma multiforme proliferation. Stem Cells Dev.

[65] Kucerova L, Skolekova S, Matuskova M, Bohac M, Kozovska Z (2013). Altered features and increased chemosensitivity of human breast cancer cells mediated by adipose tissue-derived mesenchymal stromal cells. BMC Cancer.

[66] Cousin B, Ravet E, Poglio S, De Toni F, Bertuzzi M, Lulka H, Touil I, Andre M, Grolleau JL, Peron JM, Chavoin JP, Bourin P, Penicaud L (2009). Adult stromal cells derived from human adipose tissue provoke pancreatic cancer cell death both in vitro and in vivo. PLoS One.

[67] Cavarretta IT, Altanerova V, Matuskova M, Kucerova L, Culig Z, Altaner C (2010). Adipose tissue-derived mesenchymal stem cells expressing prodrug-converting enzyme inhibit human prostate tumor growth. Mol Ther.

[68] Li TAO, Zhang C, Ding Y, Zhai WEI, Liu KUI, Bu FAN, Tu TAO, Sun L, Zhu WEI, Zhou F, Qi W, Hu J, Chen H (2015). Umbilical cord-derived mesenchymal stem cells promote proliferation and migration in MCF-7 and MDA-MB-231 breast cancer cells through activation of the ERK pathway. Oncol Rep.

[69] Ayuzawa R, Doi C, Rachakatla RS, Pyle MM, Maurya DK, Troyer D, Tamura M (2009). Naive human umbilical cord matrix derived stem cells significantly attenuate growth of human breast cancer cells in vitro and in vivo. Cancer Lett.

[70] Gauthaman K, Yee FC, Cheyyatraivendran S, Biswas A, Choolani M, Bongso A (2012). Human umbilical cord Wharton’s jelly stem cell (hWJSC) extracts inhibit cancer cell growth in vitro. J Cell Biochem.

[71] Ma Y, Hao X, Zhang S, Zhang J (2012). The in vitro and in vivo effects of human umbilical cord mesenchymal stem cells on the growth of breast cancer cells. Breast Cancer Res Treat.

[72] Rachakatla RS, Marini F, Weiss ML, Tamura M, Troyer D (2007). Development of human umbilical cord matrix stem cell-based gene therapy for experimental lung tumors. Cancer Gene Ther.

[73] Rachakatla RS, Pyle MM, Ayuzawa R, Edwards SM, Marini FC, Weiss ML, Tamura M, Troyer D (2008). Combination treatment of human umbilical cord matrix stem cell-based interferon-beta gene therapy and 5-fluorouracil significantly reduces growth of metastatic human breast cancer in SCID mouse lungs. Cancer Invest.

[74] Sasser AK, Mundy BL, Smith KM, Studebaker AW, Axel AE, Haidet AM, Fernandez SA, Hall BM (2007). Human bone marrow stromal cells enhance breast cancer cell growth rates in a cell line-dependent manner when evaluated in 3D tumor environments. Cancer Lett.

[75] Goldstein RH, Reagan MR, Anderson K, Kaplan DL, Rosenblatt M (2010). Human bone marrow-derived MSCs can home to orthotopic breast cancer tumors and promote bone metastasis. Cancer Res.

[76] Molloy AP, Martin FT, Dwyer RM, Griffin TP, Murphy M, Barry FP, O'Brien T, Kerin MJ (2009). Mesenchymal stem cell secretion of chemokines during differentiation into osteoblasts, and their potential role in mediating interactions with breast cancer cells. Int J Cancer.

[77] Rhodes LV, Muir SE, Elliott S, Guillot LM, Antoon JW, Penfornis P, Tilghman SL, Salvo VA, Fonseca JP, Lacey MR, Beckman BS, McLachlan JA, Rowan BG (2009). Adult human mesenchymal stem cells enhance breast tumorigenesis and promote hormone independence. Breast Cancer Res Treat.

[78] Mandel K, Yang Y, Schambach A, Glage S, Otte A, Hass R (2013). Mesenchymal stem cells directly interact with breast cancer cells and promote tumor cell growth in vitro and in vivo. Stem Cells Dev.

[79] Kucerova L, Kovacovicova M, Polak S, Bohac M, Fedeles J, Palencar D, Matuskova M (2011). Interaction of human adipose tissue-derived mesenchymal stromal cells with breast cancer cells. Neoplasma.

[80] Maffey A, Storini C, Diceglie C, Martelli C, Sironi L, Calzarossa C, Tonna N, Lovchik R, Delamarche E, Ottobrini L, Bianco F (2017). Mesenchymal stem cells from tumor microenvironment favour breast cancer stem cell proliferation, cancerogenic and metastatic potential, via ionotropic purinergic signalling. Sci Rep.

[81] Secchiero P, Zorzet S, Tripodo C, Corallini F, Melloni E, Caruso L, Bosco R, Ingrao S, Zavan B, Zauli G (2010). Human bone marrow mesenchymal stem cells display anti-cancer activity in SCID mice bearing disseminated non-Hodgkin’s lymphoma xenografts. PLoS One.

[82] Han IMY, Kim EO, Kim B, Jung MH, Kim SH (2014). Umbilical cord tissue-derived mesenchymal stem cells induce apoptosis in PC-3 prostate cancer cells through activation of JNK and downregulation of PI3K/AKT signalling. Stem Cell Res Ther.

[83] Wu S, Ju GQ, Du T, Zhu YJ, Liu GH (2013). Microvesicles derived from human umbilical cord Wharton’s jelly mesenchymal stem cells attenuate bladder tumor cell growth in vitro and *in vivo. PLoS One.

[84] Rhee KJ, Lee JI, Eom YW (2015). Mesenchymal stem cell-mediated effects of tumor support or suppression. Int J Mol Sci.

[85] Kidd S, Spaeth E, Klopp A, Andreeff M, Hall B, Marini F (2008). The (in) auspicious role of mesenchymal stromal cells in cancer: be it friend or foe. Cytotherapy.

[86] Roorda BD, ter Elst A, Kamps WA, de Bont ESJM (2009). Bone marrow-derived cells and tumor growth: contribution of bone marrow-derived cells to tumor micro-environments with special focus on mesenchymal stem cells. Crit Rev Oncol Hematol.

[87] Crisan M, Yap S, Casteilla L, Chen C-W, Corselli M, Park TS, Andriolo G, Sun B, Zheng B, Zhang L, Norotte C, Teng P-N, Traas J (2008). A perivascular origin for mesenchymal stem cells in multiple human organs. Cell Stem Cell.

[88] Traktuev DO, Merfeld-Clauss S, Li J, Kolonin M, Arap W, Pasqualini R, Johnstone BH, March KL (2008). A population of multipotent CD34-positive adipose stromal cells share pericyte and mesenchymal surface markers, reside in a periendothelial location, and stabilize endothelial networks. Circ Res.

[89] Mishra PJ, Humeniuk R, Medina DJ, Alexe G, Mesirov JP, Ganesan S, Glod JW, Banerjee D (2008). Carcinoma-associated fibroblast-like differentiation of human mesenchymal stem cells. Cancer Res.

[90] Kéramidas M, de Fraipont F, Karageorgis A, Moisan A, Persoons V, Richard M-J, Coll J-L, Rome C (2013). The dual effect of mscs on tumor growth and tumor angiogenesis. Stem Cell Res Ther.

[91] Shinagawa K, Kitadai Y, Tanaka M, Sumida T, Kodama M, Higashi Y, Tanaka S, Yasui W, Chayama K (2010). Mesenchymal stem cells enhance growth and metastasis of colon cancer. Int J Cancer.

[92] Kinnaird T (2004). Marrow-derived stromal cells express genes encoding a broad spectrum of arteriogenic cytokines and promote in vitro and in vivo arteriogenesis through paracrine mechanisms. Circ Res.

[93] Beckermann BM, Kallifatidis G, Groth A, Frommhold D, Apel A, Mattern J, Salnikov AV, Moldenhauer G, Wagner W, Diehlmann A, Saffrich R, Schubert M, Ho AD (2008). VEGF expression by mesenchymal stem cells contributes to angiogenesis in pancreatic carcinoma. Br J Cancer.

[94] Suzuki K, Sun R, Origuchi M, Kanehira M, Takahata T, Itoh J, Umezawa A, Kijima H, Fukuda S, Saijo Y (2011). Mesenchymal stromal cells promote tumor growth through the enhancement of neovascularization. Mol Med.

[95] Gu J, Qian H, Shen L, Zhang X, Zhu W, Huang L, Yan Y, Mao F, Zhao C, Shi Y, Xu W (2012). Gastric cancer exosomes trigger differentiation of umbilical cord derived mesenchymal stem cells to carcinoma-associated fibroblasts through TGF-β/Smad pathway. PLoS One.

[96] Otsu K, Das S, Houser SD, Quadri SK, Bhattacharya S, Bhattacharya J (2009). Concentration-dependent inhibition of angiogenesis by mesenchymal stem cells. Blood.

[97] Sotiropoulou PA, Papamichail M (2007). Immune properties of mesenchymal stem cells. Methods Mol Biol.

[98] Rutella S (2006). Tolerogenic dendritic cells: cytokine modulation comes of age. Blood.

[99] Corcione A, Benvenuto F, Ferretti E, Giunti D, Cappiello V, Cazzanti F, Risso M, Gualandi F, Mancardi GL, Pistoia V, Uccelli A (2006). Human mesenchymal stem cells modulate B-cell functions. Blood.

[100] Di Nicola M (2002). Human bone marrow stromal cells suppress T-lymphocyte proliferation induced by cellular or nonspecific mitogenic stimuli. Blood.

[101] Plumas J, Chaperot L, Richard MJ, Molens JP, Bensa JC, Favrot MC (2005). Mesenchymal stem cells induce apoptosis of activated T cells. Leukemia.

[102] Sheng H, Wang Y, Jin Y, Zhang Q, Zhang Y, Wang L, Shen B, Yin S, Liu W, Cui L, Li N (2008). A critical role of IFNγ in priming MSC-mediated suppression of T cell proliferation through up-regulation of B7-H1. Cell Res.

[103] Nasef A, Zhang YZ, Mazurier C, Bouchet S, Bensidhoum M, Francois S, Gorin NC, Lopez M, Thierry D, Fouillard L, Chapel A (2009). Selected Stro-1-enriched bone marrow stromal cells display a major suppressive effect on lymphocyte proliferation. Int J Lab Hematol.

[104] Waterman RS, Tomchuck SL, Henkle SL, Betancourt AM (2010). A new mesenchymal stem cell (MSC) paradigm: polarization into a pro-inflammatory MSC1 or an immunosuppressive MSC2 phenotype. PLoS One.

[105] Puissant B, Barreau C, Bourin P, Clavel C, Corre J, Bousquet C, Taureau C, Cousin B, Abbal M, Laharrague P, Penicaud L, Casteilla L, Blancher A. Immunomodulatory effect of human adipose tissue-derived adult stem cells: comparison with bone marrow mesenchymal stem cells. Br J Haematol. 2005;129:118–29. 10.1111/j.1365-2141.2005.05409.x 15801964

[106] Zhou C, Yang B, Tian Y, Jiao H, Zheng W, Wang J, Guan F (2011). Immunomodulatory effect of human umbilical cord Wharton’s jelly-derived mesenchymal stem cells on lymphocytes. Cell Immunol.

[107] Le Blanc K, Rasmusson I, Sundberg B, Götherström C, Hassan M, Uzunel M, Ringdén O (2004). Treatment of severe acute graft-versus-host disease with third party haploidentical mesenchymal stem cells. The Lancet.

[108] Ning H, Yang F, Jiang M, Hu L, Feng K, Zhang J, Yu Z, Li B, Xu C, Li Y, Wang J, Hu J, Lou X (2008). The correlation between cotransplantation of mesenchymal stem cells and higher recurrence rate in hematologic malignancy patients: outcome of a pilot clinical study. Leukemia.

[109] Sun B, Roh KH, Park JR (2009). Therapeutic potential of mesenchymal stromal cells in a mouse breast cancer metastasis model. Cytotherapy.

[110] Liu C, Liu Y, Xu XX, Guo X, Sun GW, Ma XJ, BM C (2016). Mesenchymal stem cells enhance the metastasis of 3D-cultured hepatocellular carcinoma cells. BMC Cancer.

[111] Martin FT, Dwyer RM, Kelly J, Khan S, Murphy JM, Curran C, Miller N, Hennessy E, Dockery P, Barry FP, O’Brien T, Kerin MJ (2010). Potential role of mesenchymal stem cells (MSCs) in the breast tumor microenvironment: stimulation of epithelial to mesenchymal transition (EMT). Breast Cancer Res Treat.

[112] Corcoran KE, Trzaska KA, Fernandes H, Bryan M, Taborga M, Srinivas V, Packman K, Patel PS, Rameshwar P (2008). Mesenchymal stem cells in early entry of breast cancer into bone marrow. PLoS One.

[113] Zhang P, Dong L, Yan K, Long HUA, Yang T-T, Dong M-Q, Zhou Y, Fan Q-Y, Ma B-A (2013). CXCR4-mediated osteosarcoma growth and pulmonary metastasis is promoted by mesenchymal stem cells through VEGF. Oncol Rep.

[114] Zhu Y, Sun Z, Han Q, Liao L, Wang J, Bian C, Li J, Yan X, Liu Y, Shao C, Zhao RC (2009). Human mesenchymal stem cells inhibit cancer cell proliferation by secreting DKK-1. Leukemia.

[115] Qiao L, Xu ZL, Zhao TJ, Ye LH, Zhang XD (2008). Dkk-1 secreted by mesenchymal stem cells inhibits growth of breast cancer cells via depression of Wnt signalling. Cancer Lett.

[116] Tong CK, Vellasamy S, Chong Tan B, Abdullah M, Vidyadaran S, Fong Seow H, Ramasamy R (2011). Generation of mesenchymal stem cell from human umbilical cord tissue using a combination enzymatic and mechanical disassociation method. Cell Biol Int.

[117] Fong CY, Chak LL, Biswas A, Tan JH, Gauthaman K, Chan WK, Bongso A (2011). Human Wharton’s jelly stem cells have unique transcriptome profiles compared to human embryonic stem cells and other mesenchymal stem cells. Stem Cell Rev.

[118] Konopleva M, Konoplev S, Hu W, Zaritskey AY, Afanasiev BV, Andreeff M (2002). Stromal cells prevent apoptosis of AML cells by up-regulation of anti-apoptotic proteins. Leukemia.

[119] Tu B, Zhu J, Liu S, Wang L, Fan QM, Hao YQ, Fan CY, Tang TT (2016). Mesenchymal stem cells promote osteosarcoma cell survival and drug resistance through activation of STAT3. Oncotarget.

[120] Lu YR, Yuan Y, Wang XJ, Wei LL, Chen YN, Cong C, Li SF, Long D, Tan WD, Mao YQ, Zhang J, Li YP, Cheng JQ (2008). The growth inhibitory effect of mesenchymal stem cells on tumor cells in vitro and in vivo. Cancer Biol Ther.

[121] Klopp AH, Gupta A, Spaeth E, Andreeff M, Marini F 3rd. Concise review: dissecting a discrepancy in the literature: do mesenchymal stem cells support or suppress tumor growth? Stem Cells. 2011;29(1):11–9. 10.1002/stem.559. 10.1002/stem.559 PMC3059412 21280155

[122] The GLOBOCAN project. http://globocan.iarc.fr/. Accessed 15 Dec 2017.

[123] Day C-P, Merlino G, Van Dyke T (2015). Preclinical mouse cancer models: a maze of opportunities and challenges. Cell.

[124] Szadvari I, Krizanova O, Babula P (2016). Athymic nude mice as an experimental model for cancer treatment. Physiol Res.

[125] de Visser KE, Korets LV, Coussens LM (2005). De novo carcinogenesis promoted by chronic inflammation is B lymphocyte dependent. Cancer Cell.

[126] Barbera-Guillem E, May KF, Nyhus JK, Nelson MB (1999). Promotion of tumor invasion by cooperation of granulocytes and macrophages activated by anti-tumor antibodies. Neoplasia.

[127] Elias WY (2016). The effect of Wharton’s jelly mesenchymal stem cells on a squamous cell carcinoma cell line. Archives in Cancer Research.

[128] Matsuzuka T, Rachakatla RS, Doi C, Maurya DK, Ohta N, Kawabata A, Pyle MM, Pickel L, Reischman J, Marini F, Troyer D, Tamura M (2010). Human umbilical cord matrix-derived stem cells expressing interferon-beta gene significantly attenuate bronchioloalveolar carcinoma xenografts in SCID mice. Lung Cancer.

[129] Yang C, Lei D, Ouyang W, Ren J, Li H, Hu J, Huang S (2014). Conditioned media from human adipose tissue-derived mesenchymal stem cells and umbilical cord-derived mesenchymal stem cells efficiently induced the apoptosis and differentiation in human glioma cell lines in vitro. Biomed Res Int.

[130] Lin HD, Fong CY, Biswas A, Choolani M, Bongso A (2016). Human umbilical cord Wharton’s jelly stem cell conditioned medium induces tumoricidal effects on lymphoma cells through hydrogen peroxide mediation. J Cell Biochem.

[131] Kim ES, Jeon HB, Lim H, Shin JH, Park SJ, Jo YK, Oh W, Yang YS, Cho DH, Kim JY (2015). Conditioned media from human umbilical cord blood-derived mesenchymal stem cells inhibits melanogenesis by promoting proteasomal degradation of MITF. PLoS One.

[132] Djouad F, Bony C, Apparailly F, Louis-Plence P, Jorgensen C, Noel D (2006). Earlier onset of syngeneic tumors in the presence of mesenchymal stem cells. Transplantation.

[133] Le Blanc K, Tammik C, Rosendahl K, Zetterberg E, Ringdén O (2003). HLA expression and immunologic properties of differentiated and undifferentiated mesenchymal stem cells. Exp Hematol.

[134] Stenderup K, Justesen J, Clausen C, Kassem M (2003). Aging is associated with decreased maximal life span and accelerated senescence of bone marrow stromal cells. Bone.

[135] Schipper BM, Marra KG, Zhang W, Donnenberg AD, Rubin JP (2008). Regional anatomic and age effects on cell function of human adipose-derived stem cells. Ann Plast Surg.

[136] Oedayrajsingh-Varma MJ, van Ham SM, Knippenberg M, Helder MN, Klein-Nulend J, Schouten TE, Ritt MJ, van Milligen FJ (2006). Adipose tissue-derived mesenchymal stem cell yield and growth characteristics are affected by the tissue-harvesting procedure. Cytotherapy.

[137] Fong CY, Subramanian A, Biswas A, Gauthaman K, Srikanth P, Hande MP, Bongso A (2010). Derivation efficiency, cell proliferation, freeze-thaw survival, stem-cell properties and differentiation of human Wharton’s jelly stem cells. Reprod Biomed Online.

[138] Nekanti U, Rao VB, Bahirvani AG, Jan M, Totey S, Ta M (2010). Long-term expansion and pluripotent marker array analysis of Wharton's jelly-derived mesenchymal stem cells. Stem Cells Dev.

[139] Bruder SP, Jaiswal N, Haynesworth SE (1997). Growth kinetics, self-renewal, and the osteogenic potential of purified human mesenchymal stem cells during extensive subcultivation and following cryopreservation. J Cell Biochem.

[140] Barlow S, Brooke G, Chatterjee K, Price G, Pelekanos R, Rossetti T, Doody M, Venter D, Pain S, Gilshenan K, Atkinson K (2008). Comparison of human placenta- and bone marrow-derived multipotent mesenchymal stem cells. Stem Cells Dev.

[141] Sotiropoulou PA, Perez SA, Salagianni M, Baxevanis CN, Papamichail M (2006). Cell culture medium composition and translational adult bone marrow-derived stem cell research. Stem Cells.

[142] Sensebe L, Bourin P, Tarte K (2011). Good manufacturing practices production of mesenchymal stem/stromal cells. Hum Gene Ther.

[143] Blázquez-Prunera A, Díez JM, Gajardo R, Grancha S. Human mesenchymal stem cells maintain their phenotype, multipotentiality, and genetic stability when cultured using a defined xeno-free human plasma fraction. Stem Cell Res Ther. 2017;8. 10.1186/s13287-017-0552-z. 10.1186/s13287-017-0552-z PMC5408419 28449711

[144] Hass R, Kasper C, Böhm S, Jacobs R. Different populations and sources of human mesenchymal stem cells (MSC): a comparison of adult and neonatal tissue-derived MSC. Cell Commun Signal. 2011;9:12. 10.1186/1478-811X-9-12 PMC3117820 21569606

[145] Hu L, Hu J, Zhao J, Liu J, Ouyang W, Yang C, Gong N, Du L, Khanal A, Chen L (2013). Side-by-side comparison of the biological characteristics of human umbilical cord and adipose tissue-derived mesenchymal stem cells. Biomed Res Int.

[146] Weiss ML, Anderson C, Medicetty S, Seshareddy KB, Weiss RJ, VanderWerff I, Troyer D, McIntosh KR (2008). Immune properties of human umbilical cord Wharton’s jelly-derived cells. Stem Cells.

[147] Kim DW, Staples M, Shinozuka K, Pantcheva P, Kang SD, Borlongan CV (2013). Wharton’s jelly-derived mesenchymal stem cells: phenotypic characterization and optimizing their therapeutic potential for clinical applications. Int J Mol Sci.

[148] Subramanian A, Fong CY, Biswas A, Bongso A (2015). Comparative characterization of cells from the various compartments of the human umbilical cord shows that the Wharton’s jelly compartment provides the best source of clinically utilizable mesenchymal stem cells. PLoS One.

[149] Karahuseyinoglu S, Cinar O, Kilic E, Kara F, Akay GG, Demiralp DO, Tukun A, Uckan D, Can A (2007). Biology of stem cells in human umbilical cord stroma: in situ and in vitro surveys. Stem Cells.

[150] Kern S, Eichler H, Stoeve J, Klüter H, Bieback K (2006). Comparative analysis of mesenchymal stem cells from bone marrow, umbilical cord blood, or adipose tissue. Stem Cells.

[151] Strioga M, Viswanathan S, Darinskas A, Slaby O, Michalek J (2012). Same or not the same? Comparison of adipose tissue-derived versus bone marrow-derived mesenchymal stem and stromal cells. Stem Cells Dev.

[152] Zhu Y, Liu T, Song K, Fan X, Ma X, Cui Z (2008). Adipose-derived stem cell: a better stem cell than BMSC. Cell Biochem Funct.

[153] Burrow KL, Hoyland JA, Richardson SM (2017). Human adipose-derived stem cells exhibit enhanced proliferative capacity and retain multipotency longer than donor-matched bone marrow mesenchymal stem cells during expansion in vitro. Stem Cells Int.

[154] Weiss ML, Medicetty S, Bledsoe AR, Rachakatla RS, Choi M, Merchav S, Luo Y, Rao MS, Velagaleti G, Troyer D (2006). Human umbilical cord matrix stem cells: preliminary characterization and effect of transplantation in a rodent model of Parkinson’s disease. Stem Cells.

[155] Salehinejad P, Alitheen NB, Ali AM, Omar AR, Mohit M, Janzamin E, Samani FS, Torshizi Z, Nematollahi-Mahani SN (2012). Comparison of different methods for the isolation of mesenchymal stem cells from human umbilical cord Wharton’s jelly. In Vitro Cell Dev Biol Anim.

[156] Yoon JH, Roh EY, Shin S, Jung NH, Song EY, Chang JY, Kim BJ, Jeon HW (2013). Comparison of explant-derived and enzymatic digestion-derived MSCs and the growth factors from Wharton’s jelly. Biomed Res Int.

[157] Lu LL, Liu YJ, Yang SG, Zhao QJ, Wang X, Gong W, Han ZB, Xu ZS, Lu YX, Liu D, Chen ZZ, Han ZC (2006). Isolation and characterization of human umbilical cord mesenchymal stem cells with hematopoiesis-supportive function and other potentials. Haematologica.

[158] Jo CH, Kim OS, Park EY, Kim BJ, Lee JH, Kang SB, Han HS, Rhee SH, Yoon KS (2008). Fetal mesenchymal stem cells derived from human umbilical cord sustain primitive characteristics during extensive expansion. Cell Tissue Res.

[159] Christodoulou I, Kolisis FN, Papaevangeliou D, Zoumpourlis V (2013). Comparative evaluation of human mesenchymal stem cells of fetal (Wharton’s jelly) and adult (adipose tissue) origin during prolonged in vitro expansion: considerations for cytotherapy. Stem Cells Int.

[160] Haynesworth SE, Baber MA, Caplan AI (1992). Cell surface antigens on human marrow-derived mesenchymal cells are detected by monoclonal antibodies. Bone.

[161] Kolaparthy LK, Sanivarapu S, Moogla S, Kutcham RS (2015). Adipose tissue - adequate, accessible regenerative material. Int J Stem Cells.

[162] Boquest AC, Shahdadfar A, Brinchmann JE, Collas P (2006). Isolation of stromal stem cells from human adipose tissue. Methods Mol Biol.

[163] Fraser JK, Schreiber R, Strem B, Zhu M, Alfonso Z, Wulur I, Hedrick MH (2006). Plasticity of human adipose stem cells toward endothelial cells and cardiomyocytes. Nat Clin Pract Cardiovasc Med.

[164] Mushahary D, Spittler A, Kasper C, Weber V, Charwat V (2018). Isolation, cultivation, and characterization of human mesenchymal stem cells. Cytometry A.

[165] Zuk PA, Zhu M, Mizuno H, Huang J, Futrell JW, Katz AJ, Benhaim P, Lorenz HP, Hedrick MH (2001). Multilineage cells from human adipose tissue: implications for cell-based therapies. Tissue Eng.

[166] Mamidi MK, Nathan KG, Singh G, Thrichelvam ST, Mohd Yusof NA, Fakharuzi NA, Zakaria Z, Bhonde R, Das AK, Majumdar AS (2012). Comparative cellular and molecular analyses of pooled bone marrow multipotent mesenchymal stromal cells during continuous passaging and after successive cryopreservation. J Cell Biochem.

[167] Mitchell JB, McIntosh K, Zvonic S, Garrett S, Floyd ZE, Kloster A, Di Halvorsen Y, Storms RW, Goh B, Kilroy G, Wu X, Gimble JM (2006). Immunophenotype of human adipose-derived cells: temporal changes in stromal-associated and stem cell-associated markers. Stem Cells.

[168] Hao H, Chen G, Liu J, Ti D, Zhao Y, Xu S, Fu X, Han W (2013). Culturing on Wharton’s jelly extract delays mesenchymal stem cell senescence through p53 and p16INK4a/pRb pathways. PLoS One.

[169] Ding DC, Chou HL, Chang YH, Hung WT, Liu HW, Chu TY (2016). Characterization of HLA-G and related immunosuppressive effects in human umbilical cord stroma-derived stem cells. Cell Transplant.

[170] La Rocca G, Anzalone R, Corrao S, Magno F, Loria T, Lo Iacono M, Di Stefano A, Giannuzzi P, Marasa L, Cappello F, Zummo G, Farina F (2009). Isolation and characterization of Oct-4+/HLA-G+ mesenchymal stem cells from human umbilical cord matrix: differentiation potential and detection of new markers. Histochem Cell Biol.

[171] Lund RD, Wang S, Lu B, Girman S, Holmes T, Sauve Y, Messina DJ, Harris IR, Kihm AJ, Harmon AM, Chin FY, Gosiewska A, Mistry SK (2007). Cells isolated from umbilical cord tissue rescue photoreceptors and visual functions in a rodent model of retinal disease. Stem Cells.

[172] Drela K, Sarnowska A, Siedlecka P, Szablowska-Gadomska I, Wielgos M, Jurga M, Lukomska B, Domanska-Janik K (2014). Low oxygen atmosphere facilitates proliferation and maintains undifferentiated state of umbilical cord mesenchymal stem cells in an hypoxia inducible factor-dependent manner. Cytotherapy.

[173] Venugopal P, Balasubramanian S, Majumdar AS, Ta M (2011). Isolation, characterization, and gene expression analysis of Wharton’s jelly-derived mesenchymal stem cells under xeno-free culture conditions. Stem Cells Cloning.

[174] Simoes IN, Boura JS, dos Santos F, Andrade PZ, Cardoso CM, Gimble JM, da Silva CL, Cabral JM (2013). Human mesenchymal stem cells from the umbilical cord matrix: successful isolation and ex vivo expansion using serum-/xeno-free culture media. Biotechnol J.

[175] Digirolamo CM, Stokes D, Colter D, Phinney DG, Class R, Prockop DJ (1999). Propagation and senescence of human marrow stromal cells in culture: a simple colony-forming assay identifies samples with the greatest potential to propagate and differentiate. Br J Haematol.

[176] Madeira A, da Silva CL, dos Santos F, Camafeita E, Cabral JM, Sa-Correia I (2012). Human mesenchymal stem cell expression program upon extended ex-vivo cultivation, as revealed by 2-DE-based quantitative proteomics. PLoS One.

[177] Dai L-J, Li HY, Guan L-X, Ritchie G, Zhou JX (2009). The therapeutic potential of bone marrow-derived mesenchymal stem cells on hepatic cirrhosis. Stem Cell Res.

[178] Bertolo A, Mehr M, Janner-Jametti T, Graumann U, Aebli N, Baur M, Ferguson SJ, Stoyanov JV (2016). An in vitro expansion score for tissue-engineering applications with human bone marrow-derived mesenchymal stem cells. J Tissue Eng Regen Med.

[179] Skinner KA, Silberman H, Sposto R, Silverstein MJ (2001). Palpable breast cancers are inherently different from nonpalpable breast cancers. Ann Surg Oncol.

[180] Liu Z, Lee Y, Jang J, Li Y, Han X, Yokoi K, Ferrari M, Zhou L, Qin L (2015). Microfluidic cytometric analysis of cancer cell transportability and invasiveness. Sci Rep.

[181] Troyer DL, Weiss ML (2008). Wharton’s jelly-derived cells are a primitive stromal cell population. Stem Cells.

[182] Fong CY, Richards M, Manasi N, Biswas A, Bongso A (2007). Comparative growth behaviour and characterization of stem cells from human Wharton’s jelly. Reprod Biomed Online.

[183] Najar M, Raicevic G, Boufker HI, Fayyad Kazan H, De Bruyn C, Meuleman N, Bron D, Toungouz M, Lagneaux L (2010). Mesenchymal stromal cells use PGE2 to modulate activation and proliferation of lymphocyte subsets: combined comparison of adipose tissue, Wharton’s Jelly and bone marrow sources. Cell Immunol.

[184] Jeon BG, Kumar BM, Kang EJ, Ock SA, Lee SL, Kwack DO, Byun JH, Park BW, Rho GJ (2011). Characterization and comparison of telomere length, telomerase and reverse transcriptase activity and gene expression in human mesenchymal stem cells and cancer cells of various origins. Cell Tissue Res.

[185] Tang Q, Chen Q, Lai X, Liu S, Chen Y, Zheng Z, Xie Q, Maldonado M, Cai Z, Qin S, Ho G, Ma L (2013). Malignant transformation potentials of human umbilical cord mesenchymal stem cells both spontaneously and via 3-methycholanthrene induction. PLoS One.

[186] Wagner W, Ho AD, Zenke M (2010). Different facets of aging in human mesenchymal stem cells. Tissue Eng Part B Rev.

[187] Ryu E, Hong S, Kang J, Woo J, Park J, Lee J, Seo JS (2008). Identification of senescence-associated genes in human bone marrow mesenchymal stem cells. Biochem Biophys Res Commun.

[188] Rosland GV, Svendsen A, Torsvik A, Sobala E, McCormack E, Immervoll H, Mysliwietz J, Tonn JC, Goldbrunner R, Lonning PE, Bjerkvig R, Schichor C (2009). Long-term cultures of bone marrow-derived human mesenchymal stem cells frequently undergo spontaneous malignant transformation. Cancer Res.

[189] Subramanian A, Shu-Uin G, Kae-Siang N, Gauthaman K, Biswas A, Choolani M, Bongso A, Chui-Yee F (2012). Human umbilical cord Wharton’s jelly mesenchymal stem cells do not transform to tumor-associated fibroblasts in the presence of breast and ovarian cancer cells unlike bone marrow mesenchymal stem cells. J Cell Biochem.

[190] Xu WT, Bian ZY, Fan QM, Li G, Tang TT (2009). Human mesenchymal stem cells (hMSCs) target osteosarcoma and promote its growth and pulmonary metastasis. Cancer Lett.

[191] Mohseny AB, Szuhai K, Romeo S, Buddingh EP, Briaire-de Bruijn I, de Jong D, van Pel M, Cleton-Jansen AM, Hogendoorn PC (2009). Osteosarcoma originates from mesenchymal stem cells in consequence of aneuploidization and genomic loss of Cdkn2. J Pathol.

[192] Bernardo ME, Zaffaroni N, Novara F, Cometa AM, Avanzini MA, Moretta A, Montagna D, Maccario R, Villa R, Daidone MG, Zuffardi O, Locatelli F (2007). Human bone marrow derived mesenchymal stem cells do not undergo transformation after long-term in vitro culture and do not exhibit telomere maintenance mechanisms. Cancer Res.

[193] He LYZ, Wan Y, Song J (2014). A shorter telomere is the key factor in preventing cultured human mesenchymal stem cells from senescence escape. Histochem Cell Biol.

[194] Garcia S, Bernad A, Martin MC, Cigudosa JC, Garcia-Castro J, de la Fuente R (2010). Pitfalls in spontaneous in vitro transformation of human mesenchymal stem cells. Exp Cell Res.

[195] Lalu MM, McIntyre L, Pugliese C, Fergusson D, Winston BW, Marshall JC, Granton J, Stewart DJ, Canadian Critical Care Trials Group (2012). Safety of cell therapy with mesenchymal stromal cells (SafeCell): a systematic review and meta-analysis of clinical trials. PLoS One.

[196] Coulson-Thomas VJ, Coulson-Thomas YM, Gesteira TF, Kao WW (2016). Extrinsic and intrinsic mechanisms by which mesenchymal stem cells suppress the immune system. Ocul Surf.

[197] Amable PR, Teixeira MV, Carias RB, Granjeiro JM, Borojevic R (2014). Protein synthesis and secretion in human mesenchymal cells derived from bone marrow, adipose tissue and Wharton’s jelly. Stem Cell Res Ther.

[198] Dabrowski FA, Burdzinska A, Kulesza A, Sladowska A, Zolocinska A, Gala K, Paczek L, Wielgos M (2017). Comparison of the paracrine activity of mesenchymal stem cells derived from human umbilical cord, amniotic membrane and adipose tissue. J Obstet Gynaecol Res.

[199] Torsvik A, Bjerkvig R (2013). Mesenchymal stem cell signaling in cancer progression. Cancer Treat Rev.

[200] Shen CJ, Chan TF, Chen CC, Hsu YC, Long CY, Lai CS (2016). Human umbilical cord matrix-derived stem cells expressing interferon-beta gene inhibit breast cancer cells via apoptosis. Oncotarget.

[201] Hendijani F, Javanmard SH, Sadeghi-aliabadi H (2015). Human Wharton’s jelly mesenchymal stem cell secretome display antiproliferative effect on leukemia cell line and produce additive cytotoxic effect in combination with doxorubicin. Tissue Cell.

[202] Hendijani F, Javanmard Sh H, Rafiee L, Sadeghi-Aliabadi H (2015). Effect of human Wharton’s jelly mesenchymal stem cell secretome on proliferation, apoptosis and drug resistance of lung cancer cells. Res Pharm Sci.

[203] Zhang X, Tu H, Yang Y, Fang L, Wu Q, Li J (2017). Mesenchymal stem cell-derived extracellular vesicles: roles in tumor growth, progression, and drug resistance. Stem Cells Int.

[204] Sharma A (2018). Role of stem cell derived exosomes in tumor biology. Int J Cancer.

[205] Del Fattore A, Luciano R, Saracino R, Battafarano G, Rizzo C, Pascucci L, Alessandri G, Pessina A, Perrotta A, Fierabracci A, Muraca M (2015). Differential effects of extracellular vesicles secreted by mesenchymal stem cells from different sources on glioblastoma cells. Expert Opin Biol Ther.

[206] Du T, Ju G, Wu S, Cheng Z, Cheng J, Zou X, Zhang G, Miao S, Liu G, Zhu Y (2014). Microvesicles derived from human Wharton’s jelly mesenchymal stem cells promote human renal cancer cell growth and aggressiveness through induction of hepatocyte growth factor. PLoS One.

[207] Le Blanc K (2003). Immunomodulatory effects of fetal and adult mesenchymal stem cells. Cytotherapy.

[208] Yoo KH, Jang IK, Lee MW, Kim HE, Yang MS, Eom Y, Lee JE, Kim YJ, Yang SK, Jung HL, Sung KW, Kim CW, Koo HH (2009). Comparison of immunomodulatory properties of mesenchymal stem cells derived from adult human tissues. Cell Immunol.

[209] Maurya DK, Doi C, Kawabata A, Pyle MM, King C, Wu Z, Troyer D, Tamura M (2010). Therapy with un-engineered naive rat umbilical cord matrix stem cells markedly inhibits growth of murine lung adenocarcinoma. BMC Cancer.

[210] Doi C, Maurya DK, Pyle MM, Troyer D, Tamura M (2010). Cytotherapy with naive rat umbilical cord matrix stem cells significantly attenuates growth of murine pancreatic cancer cells and increases survival in syngeneic mice. Cytotherapy.

[211] Ganta C, Chiyo D, Ayuzawa R, Rachakatla R, Pyle M, Andrews G, Weiss M, Tamura M, Troyer D (2009). Rat umbilical cord stem cells completely abolish rat mammary carcinomas with no evidence of metastasis or recurrence 100 days post-tumor cell inoculation. Cancer Res.

[212] Waterman RS, Henkle SL, Betancourt AM (2012). Mesenchymal stem cell 1 (MSC1)-based therapy attenuates tumor growth whereas MSC2-treatment promotes tumor growth and metastasis. PLoS One.

[213] Zheng H, Zou W, Shen J, Xu L, Wang S, Fu YX, Fan W (2016). Opposite effects of coinjection and distant injection of mesenchymal stem cells on breast tumor cell growth. Stem Cells Transl Med.

[214] Prasanna SJ, Gopalakrishnan D, Shankar SR, Vasandan AB (2010). Pro-inflammatory cytokines, IFNgamma and TNFalpha, influence immune properties of human bone marrow and Wharton jelly mesenchymal stem cells differentially. PLoS One.

[215] Ribeiro A, Laranjeira P, Mendes S, Velada I, Leite C, Andrade P, Santos F, Henriques A, Grãos M, Cardoso CMP, Martinho A, Pais M, da Silva C (2013). Mesenchymal stem cells from umbilical cord matrix, adipose tissue and bone marrow exhibit different capability to suppress peripheral blood B, natural killer and T cells. Stem Cell Res Ther.

[216] Raicevic G, Rouas R, Najar M, Stordeur P, Boufker HI, Bron D, Martiat P, Goldman M, Nevessignsky MT, Lagneaux L (2010). Inflammation modifies the pattern and the function of Toll-like receptors expressed by human mesenchymal stromal cells. Hum Immunol.

[217] Coulson-Thomas VJ, Gesteira TF, Hascall V, Kao W (2014). Umbilical cord mesenchymal stem cells suppress host rejection: the role of the glycocalyx. J Biol Chem.

[218] Deuse T, Stubbendorff M, Tang-Quan K, Phillips N, Kay MA, Eiermann T, Phan TT, Volk HD, Reichenspurner H, Robbins RC, Schrepfer S (2011). Immunogenicity and immunomodulatory properties of umbilical cord lining mesenchymal stem cells. Cell Transplant.

[219] Chan JL, Tang KC, Patel AP, Bonilla LM, Pierobon N, Ponzio NM, Rameshwar P (2006). Antigen-presenting property of mesenchymal stem cells occurs during a narrow window at low levels of interferon-gamma. Blood.

[220] Fang L, Lange C, Engel M, Zander AR, Fehse B (2006). Sensitive balance of suppressing and activating effects of mesenchymal stem cells on T-cell proliferation. Transplantation.

[221] Lila N, Rouas-Freiss N, Dausset J, Carpentier A, Carosella ED (2001). Soluble HLA-G protein secreted by allo-specific CD4+ T cells suppresses the allo-proliferative response: a CD4+ T cell regulatory mechanism. Proc Natl Acad Sci U S A.

[222] Ristich V, Liang S, Zhang W, Wu J, Horuzsko A (2005). Tolerization of dendritic cells by HLA-G. Eur J Immunol.

[223] Cho PS, Messina DJ, Hirsh EL, Chi N, Goldman SN, Lo DP, Harris IR, Popma SH, Sachs DH, Huang CA (2008). Immunogenicity of umbilical cord tissue derived cells. Blood.

[224] Nowakowski A, Drela K, Rozycka J, Janowski M, Lukomska B (2016). Engineered mesenchymal stem cells as an anti-cancer Trojan Horse. Stem Cells Dev.

[225] Hagenhoff A, Bruns CJ, Zhao Y, von Luttichau I, Niess H, Spitzweg C, Nelson PJ (2016). Harnessing mesenchymal stem cell homing as an anticancer therapy. Expert Opin Biol Ther.

[226] Loebinger MR, Eddaoudi A, Davies D, Janes SM (2009). Mesenchymal stem cell delivery of TRAIL can eliminate metastatic cancer. Cancer Res.

[227] Studeny M, Marini FC, Champlin RE, Zompetta C, Fidler IJ, Andreeff M (2002). Bone marrow-derived mesenchymal stem cells as vehicles for interferon-β delivery into tumors. Cancer Res.

[228] Xin H, Sun R, Kanehira M, Takahata T, Itoh J, Mizuguchi H, Saijo Y (2009). Intratracheal delivery of CX3CL1-expressing mesenchymal stem cells to multiple lung tumors. Mol Med.

[229] Yang X, Du J, Xu X, Xu C, Song W (2014). IFN-gamma-secreting-mesenchymal stem cells exert an antitumor effect in vivo via the TRAIL pathway. J Immunol Res.

[230] Ando M, Hoyos V, Yagyu S, Tao W, Ramos CA, Dotti G, Brenner MK, Bouchier-Hayes L (2014). Bortezomib sensitizes non-small cell lung cancer to mesenchymal stromal cell-delivered inducible caspase-9-mediated cytotoxicity. Cancer Gene Ther.

[231] Martinez-Quintanilla J, Bhere D, Heidari P, He D, Mahmood U, Shah K (2013). Therapeutic efficacy and fate of bimodal engineered stem cells in malignant brain tumors. Stem Cells.

[232] Sasportas LS, Kasmieh R, Wakimoto H, Hingtgen S, van de Water JA, Mohapatra G, Figueiredo JL, Martuza RL, Weissleder R, Shah K (2009). Assessment of therapeutic efficacy and fate of engineered human mesenchymal stem cells for cancer therapy. Proc Natl Acad Sci U S A.

[233] Jung JH, Kim AA, Chang DY, Park YR, Suh-Kim H, Kim SS (2015). Three-dimensional assessment of bystander effects of mesenchymal stem cells carrying a cytosine deaminase gene on glioma cells. Am J Cancer Res.

[234] Menon LG, Yang HW, Kim SK, Black PM, Carroll RS, KK (2009). Human bone marrow-derived mesenchymal stromal cells expressing S-TRAIL as a cellular delivery vehicle for human glioma therapy. Stem Cells Dev.

[235] Yang B, Wu X, Mao Y, Bao W, Gao L, Zhou P, Xie R, Zhou L, Zhu J (2009). Dual-targeted antitumor effects against brainstem glioma by intravenous delivery of tumor necrosis factor-related, apoptosis-inducing, ligand-engineered human mesenchymal stem cells. Neurosurgery.

[236] Nakamizo A, Marini F, Amano T, Khan A, Studeny M, Gumin J (2005). Human bone marrow-derived mesenchymal stem cells in the treatment of gliomas. Cancer Res.

[237] Wang Q, Zhang Z, Ding T, Chen Z, Zhang T (2013). Mesenchymal stem cells overexpressing PEDF decrease the angiogenesis of gliomas. Biosci Rep.

[238] Matuskova M, Hlubinova K, Pastorakova A, Hunakova L, Altanerova V, Altaner C, Kucerova L (2010). HSV-tk expressing mesenchymal stem cells exert bystander effect on human glioblastoma cells. Cancer Lett.

[239] Matuskova M, Baranovicova L, Kozovska Z, Durinikova E, Pastorakova A, Hunakova L, Waczulikova I, Nencka R, Kucerova L (2012). Intrinsic properties of tumor cells have a key impact on the bystander effect mediated by genetically engineered mesenchymal stromal cells. J Gene Med.

[240] Choi SA, Lee JY, Kwon SE, Wang KC, Phi JH, Choi JW, Jin X, Lim JY, Kim H, Kim SK (2015). Human adipose tissue-derived mesenchymal stem cells target brain tumor-initiating cells. PLoS One.

[241] de Melo SM, Bittencourt S, Ferrazoli EG, da Silva CS, da Cunha FF, da Silva FH, Stilhano RS, Denapoli PM, Zanetti BF, Martin PK, Silva LM, dos Santos AA, Baptista LS, Longo BM, Han SW (2015). The anti-tumor effects of adipose tissue mesenchymal stem cell transduced with HSV-tk gene on U-87-driven brain tumor. PLoS One.

[242] Ohta N, Ishiguro S, Kawabata A, Uppalapati D, Pyle M, Troyer D, De S, Zhang Y, Becker KG, Tamura M (2015). Human umbilical cord matrix mesenchymal stem cells suppress the growth of breast cancer by expression of tumor suppressor genes. PLoS One.

[243] Wang GX, Zhan YA, Hu HL, Wang Y, Fu B (2012). Mesenchymal stem cells modified to express interferon-beta inhibit the growth of prostate cancer in a mouse model. J Int Med Res.

[244] Xie C, Xie DY, Lin BL, Zhang GL, Wang PP, Peng L, Gao ZL (2013). Interferon-beta gene-modified human bone marrow mesenchymal stem cells attenuate hepatocellular carcinoma through inhibiting AKT/FOXO3a pathway. Br J Cancer.

[245] Kidd S, Caldwell L, Dietrich M, Samudio I, Spaeth EL, Watson K, Shi Y, Abbruzzese J, Konopleva M, Andreeff M, Marini FC (2010). Mesenchymal stromal cells alone or expressing interferon-beta suppress pancreatic tumors in vivo, an effect countered by anti-inflammatory treatment. Cytotherapy.

[246] Elzaouk L, Moelling K, Pavlovic J (2006). Anti-tumor activity of mesenchymal stem cells producing IL-12 in a mouse melanoma model. ExpDermatol.

[247] Zhang Y, Wang J, Ren M, Li M, Chen D, Chen J, Shi F, Wang X, Dou J (2014). Gene therapy of ovarian cancer using IL-21-secreting human umbilical cord mesenchymal stem cells in nude mice. Journal of Ovarian Research.

[248] Gao P, Ding Q, Wu Z, Jiang H, Fang Z (2010). Therapeutic potential of human mesenchymal stem cells producing IL-12 in a mouse xenograft model of renal cell carcinoma. Cancer Lett.

[249] Grisendi G, Bussolari R, Cafarelli L, Petak I, Rasini V, Veronesi E, De Santis G, Spano C, Tagliazzucchi M, Barti-Juhasz H, Scarabelli L, Bambi F, Frassoldati A (2010). Adipose-derived mesenchymal stem cells as stable source of tumor necrosis factor-related apoptosis-inducing ligand delivery for cancer therapy. Cancer Res.

[250] Ciavarella S, Grisendi G, Dominici M, Tucci M, Brunetti O, Dammacco F, Silvestris F (2012). In vitro anti-myeloma activity of TRAIL-expressing adipose-derived mesenchymal stem cells. Br J Haematol.

[251] Luetzkendorf J, Mueller LP, Mueller T, Caysa H, Nerger K, Schmoll HJ (2010). Growth inhibition of colorectal carcinoma by lentiviral TRAIL-transgenic human mesenchymal stem cells requires their substantial intratumoral presence. J Cell Mol Med.

[252] Yan C, Yang M, Li Z, Li S, Hu X, Fan D, Zhang Y, Wang J, Xiong D (2014). Suppression of orthotopically implanted hepatocarcinoma in mice by umbilical cord-derived mesenchymal stem cells with sTRAIL gene expression driven by AFP promoter. Biomaterials.

[253] Yan C, Song X, Yu W, Wei F, Li H, Lv M, Zhang X, Ren X (2016). Human umbilical cord mesenchymal stem cells delivering sTRAIL home to lung cancer mediated by MCP-1/CCR2 axis and exhibit antitumor effects. Tumor Biol.

[254] Ghaedi M, Soleimani M, Taghvaie NM, Sheikhfatollahi M, Azadmanesh K, Lotfi AS, Wu J (2011). Mesenchymal stem cells as vehicles for targeted delivery of anti-angiogenic protein to solid tumors. J Gene Med.

[255] Zhu Y, Cheng M, Yang Z, Zeng CY, Chen J, Xie Y, Luo SW, Zhang KH, Zhou SF, Lu NH (2014). Mesenchymal stem cell-based NK4 gene therapy in nude mice bearing gastric cancer xenografts. Drug Des Devel Ther.

[256] Tyciakova S, Matuskova M, Bohovic R, Polakova K, Toro L, Skolekova S, Kucerova L (2015). Genetically engineered mesenchymal stromal cells producing TNFα have tumor suppressing effect on human melanoma xenograft. J Gene Med.

[257] Zhang X, Xu W, Qian H, Zhu W, Zhang R (2011). J. Mesenchymal stem cells modified to express lentivirus TNF-α Tumstatin (45-132) inhibit the growth of prostate cancer. J Cell Mol Med.

[258] Wu N, Zhang YL, Wang HT, Li DW, Dai HJ, Zhang QQ, Zhang J, Ma Y, Xia Q, Bian JM, Hang HL (2016). Overexpression of hepatocyte nuclear factor 4alpha in human mesenchymal stem cells suppresses hepatocellular carcinoma development through Wnt/beta-catenin signaling pathway downregulation. Cancer Biol Ther.

[259] Wang S, El-Deiry WS (2003). TRAIL and apoptosis induction by TNF-family death receptors. Oncogene.

[260] Yagita H, Takeda K, Hayakawa Y, Smyth MJ, Okumura K (2004). TRAIL and its receptors as targets for cancer therapy. Cancer Sci.

[261] Ashkenazi A (2002). Targeting death and decoy receptors of the tumor-necrosis factor superfamily. Nat Rev Cancer.

[262] ClinicalTrials.gov. https://clinicaltrials.gov/. Accessed 2 Feb 2018.

[263] Zhang J, Kale V, Chen M (2015). Gene-directed enzyme prodrug therapy. AAPS J.

[264] Huber BE, Austin EA, Richards CA, Davis ST, Good SS (1994). Metabolism of 5-fluorocytosine to 5-fluorouracil in human colorectal tumor cells transduced with the cytosine deaminase gene: significant antitumor effects when only a small percentage of tumor cells express cytosine deaminase. Proc Natl Acad Sci U S A.

[265] Freeman SM, Abboud CN, Whartenby KA, Packman CH, Koeplin DS, Moolten FL, Abraham GN (1993). The “bystander effect”: tumor regression when a fraction of the tumor mass is genetically modified. Cancer Res.

[266] Elshami AA, Saavedra A, Zhang H, Kucharczuk JC, Spray DC, Fishman GI, Amin KM, Kaiser LR, Albelda SM (1996). Gap junctions play a role in the ‘bystander effect’ of the herpes simplex virus thymidine kinase/ganciclovir system in vitro. Gene Ther.

[267] Namba H, Iwadate Y, Kawamura K, Sakiyama S, Tagawa M (2001). Efficacy of the bystander effect in the herpes simplex virus thymidine kinase-mediated gene therapy is influenced by the expression of connexin43 in the target cells. Cancer Gene Ther.

[268] Colombo BM, Benedetti S, Ottolenghi S, Mora M, Pollo B, Poli G, Finocchiaro G (1995). The “bystander effect”: association of U-87 cell death with ganciclovir-mediated apoptosis of nearby cells and lack of effect in athymic mice. Hum Gene Ther.

[269] Freeman SM, Ramesh R, Marrogi AJ (1997). Immune system in suicide-gene therapy. Lancet.

[270] Yamamoto S, Suzuki S, Hoshino A, Akimoto M, Shimada T (1997). Herpes simplex virus thymidine kinase/ganciclovir-mediated killing of tumor cell induces tumor-specific cytotoxic T cells in mice. Cancer Gene Ther.

[271] Erbs P, Regulier E, Kintz J, Leroy P, Poitevin Y, Exinger F, Jund R, Mehtali M (2000). In vivo cancer gene therapy by adenovirus-mediated transfer of a bifunctional yeast cytosine deaminase/uracil phosphoribosyltransferase fusion gene. Cancer Res.

[272] Koyama F, Sawada H, Hirao T, Fujii H, Hamada H, Nakano H (2000). Combined suicide gene therapy for human colon cancer cells using adenovirus-mediated transfer of escherichia coli cytosine deaminase gene and Escherichia coli uracil phosphoribosyltransferase gene with 5-fluorocytosine. Cancer Gene Ther.

[273] Matuskova M, Kozovska Z, Toro L, Durinikova E, Tyciakova S, Cierna Z, Bohovic R, Kucerova L (2015). Combined enzyme/prodrug treatment by genetically engineered AT-MSC exerts synergy and inhibits growth of MDA-MB-231 induced lung metastases. J Exp Clin Cancer Res.

[274] Toro L, Bohovic R, Matuskova M, Smolkova B, Kucerova L. Metastatic ovarian cancer can be efficiently treated by genetically modified mesenchymal stromal cells. Stem Cells Dev. 2016. 10.1089/scd.2016.0064. 10.1089/scd.2016.0064 27539058

[275] Kucerova L, Matuskova M, Pastorakova A, Tyciakova S, Jakubikova J, Bohovic R, Altanerova V, Altaner C (2008). Cytosine deaminase expressing human mesenchymal stem cells mediated tumor regression in melanoma bearing mice. J Gene Med.

[276] NguyenThai QA, Sharma N, Luong do H, Sodhi SS, Kim JH, Kim N, Oh SJ, Jeong DK (2015). Targeted inhibition of osteosarcoma tumor growth by bone marrow-derived mesenchymal stem cells expressing cytosine deaminase/5-fluorocytosine in tumor-bearing mice. J Gene Med.

[277] Kucerova L, Altanerova V, Matuskova M, Tyciakova S, Altaner C (2007). Adipose tissue-derived human mesenchymal stem cells mediated prodrug cancer gene therapy. Cancer Res.

[278] Abrate A, Buono R, Canu T, Esposito A, Del Maschio A, Luciano R, Bettiga A, Colciago G, Guazzoni G, Benigni F, Hedlund P, Altaner C, Montorsi F (2014). Mesenchymal stem cells expressing therapeutic genes induce autochthonous prostate tumor regression. Eur J Cancer.

[279] Lee WY, Zhang T, Lau CP, Wang CC, Chan KM, Li G (2013). Immortalized human fetal bone marrow-derived mesenchymal stromal cell expressing suicide gene for anti-tumor therapy in vitro and in vivo. Cytotherapy.

[280] Vilalta M, Dégano IR, Bagó J, Aguilar E, Gambhir SS, Rubio N, Blanco J. Human adipose tissue-derived mesenchymal stromal cells as vehicles for tumor bystander effect: a model based on bioluminescence imaging. Gene Ther. 2009;16:547–57. 10.1038/gt.2008.176 19092860

[281] Thomas MN, Michl M, Angele MK, Huss R, Ganther C, Nelson PJ, Bruns CJ, Heinemann V, Niess H vEJ (2015). Treatment of advanced gastrointestinal tumorswith genetically modified autologous mesenchymal stromal cells (TREAT-ME1): study protocol of a phase I/II clinical trial. BMC Cancer.

[282] Kay MA, Glorioso JC, Naldini L (2001). Viral vectors for gene therapy: the art of turning infectious agents into vehicles of therapeutics. Nat Med.

[283] Marshall E (1999). CLINICAL TRIALS: gene therapy death prompts review of adenovirus vector. Science.

[284] Hacein-Bey-Abina S, von Kalle C, Schmidt M, Le Deist F, Wulffraat N, McIntyre E, Radford I, Villeval J-L, Fraser CC, Cavazzana-Calvo M, Fischer A (2003). A serious adverse event after successful gene therapy for x-linked severe combined immunodeficiency. N Engl J Med.

[285] Walther W, Stein U (2000). Viral vectors for gene transfer: a review of their use in the treatment of human diseases. Drugs.

[286] Zhang XY, La Russa VF, Bao L, Kolls J, Schwarzenberger P, Reiser J (2002). Lentiviral vectors for sustained transgene expression in human bone marrow-derived stromal cells. Mol Ther.

[287] Severino P, Szymanski M, Favaro M, Azzoni AR, Chaud MV, Santana MH, Silva AM, Souto EB (2015). Development and characterization of a cationic lipid nanocarrier as non-viral vector for gene therapy. Eur J Pharm Sci.

[288] Jiang X, Fitch S, Wang C, Wilson C, Li J, Grant GA, Yang F (2016). Nanoparticle engineered TRAIL-overexpressing adipose-derived stem cells target and eradicate glioblastoma via intracranial delivery. Proc Natl Acad Sci U S A.

[289] Kim SM, Lim JY, Park SI, Jeong CH, Oh JH, Jeong M, Oh W, Park SH, Sung YC, Jeun SS (2008). Gene therapy using TRAIL-secreting human umbilical cord blood-derived mesenchymal stem cells against intracranial glioma. Cancer Res.

[290] Bernardo ME, Fibbe WE (2013). Mesenchymal stromal cells: sensors and switchers of inflammation. Cell Stem Cell.

[291] Hanahan D, Coussens LM (2012). Accessories to the crime: functions of cells recruited to the tumor microenvironment. Cancer Cell.

