# Supplementary figures and images for: Mesenchymal stem cells in preclinical cancer cytotherapy: a systematic review
Source: Stem Cell Res Ther. 2018 Dec 7;9:336. doi: 10.1186/s13287-018-1078-8 (PMC6286545; doi:10.1186/s13287-018-1078-8)

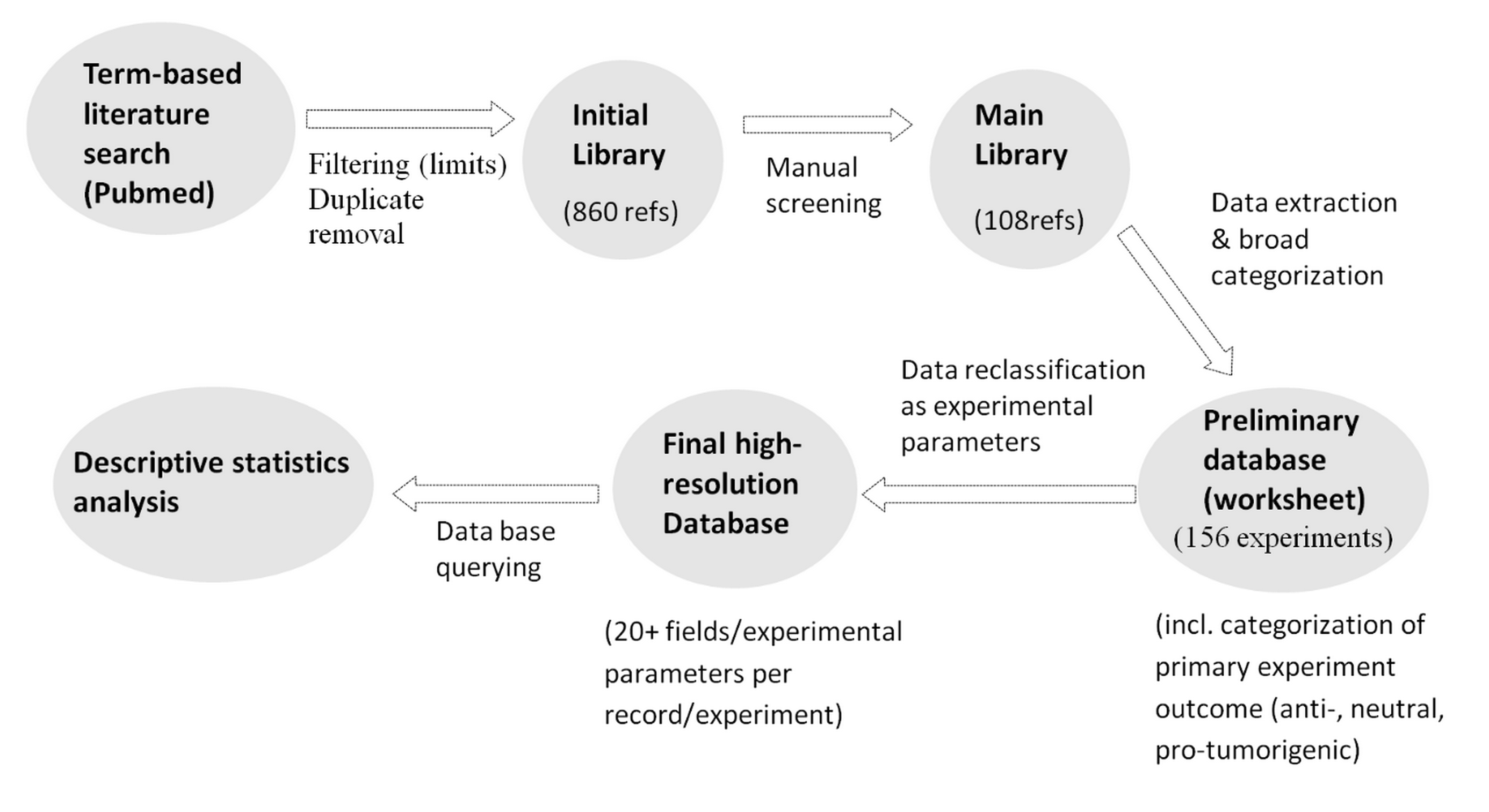

Supplement: Supplementary file 2 — Figure S2. Overview of meta-analysis methodology (TIF 12282 kb) [file 13287_2018_1078_MOESM2_ESM.tif]

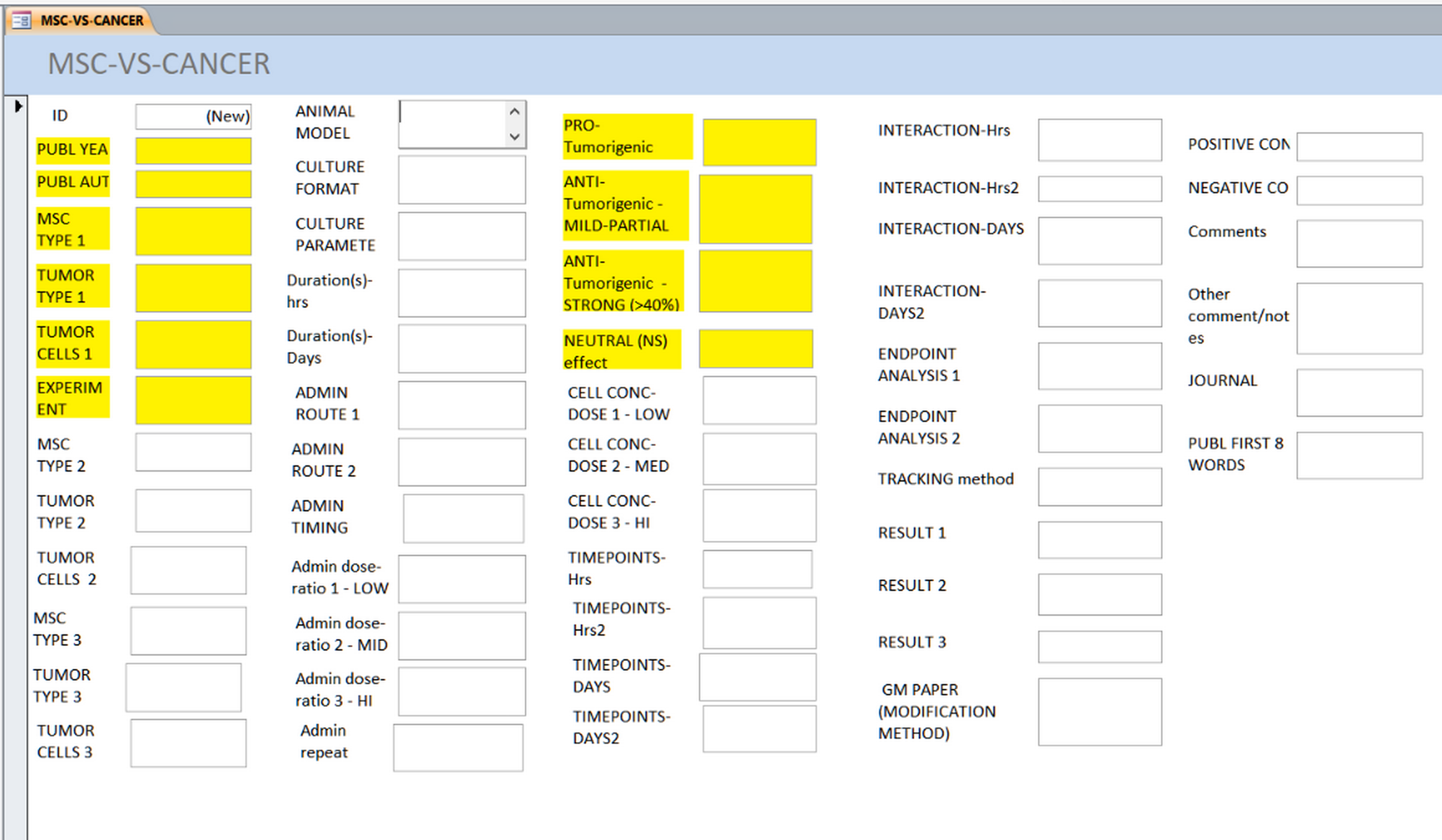

Supplement: Supplementary file 3 — Figure S3. Example of a database form used to record experimental data used in the meta-analysis. Field titles correspond to the parameters comprising each of the in vitro and in vivo experiments as described in the methodology and results sections of the relevant articles. (TIF 9196 kb) [file 13287_2018_1078_MOESM3_ESM.tif]

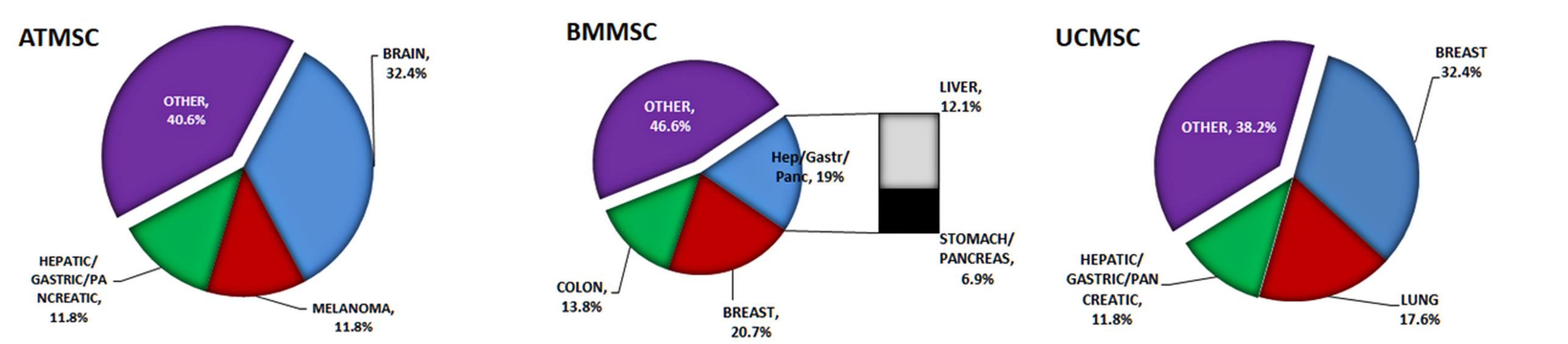

Supplement: Supplementary file 4 — Figure S4. Distribution of the three most frequently associated tumors in relation to MSC effectors. Sample sizes: adipose-derived MSC (AT-MSC) = 32, bone marrow-derived MSC (BM-MSC) = 56, umbilical cord-derived MSC (UC-MSC) = 34. (TIF 4256 kb) [file 13287_2018_1078_MOESM4_ESM.tif]

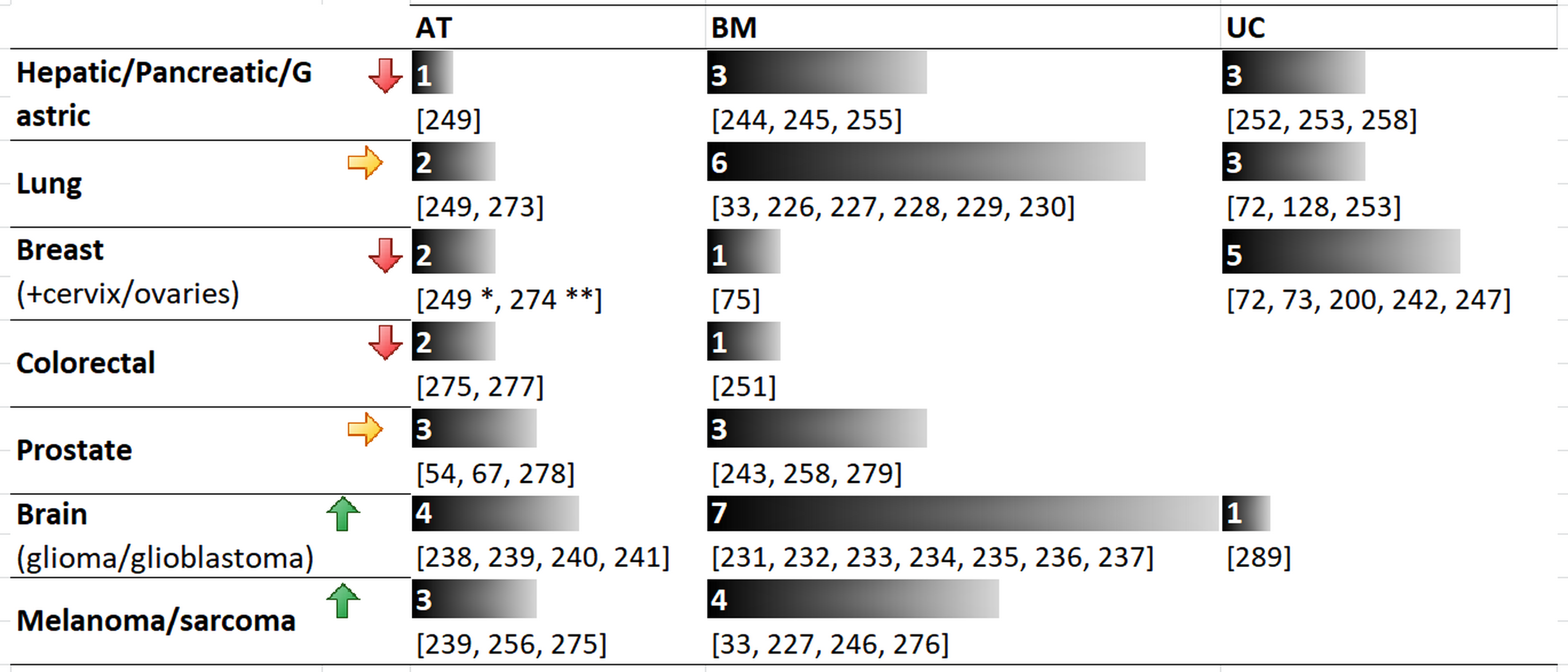

Supplement: Supplementary file 6 — Figure S6. List and frequency distribution of studies employing the use of genetically modified stem cells (GM-MSC) of human adipose tissue (AT), bone marrow (BM), and fetal umbilical cord (UC) matrix origin. In each row of the table, the length of black-gradient filled horizontal bars is proportional to the total number of studies (value within bar) relevant to specific GM-MSC/tumor combinations; the list of respective citations is shown under the bars. Cancer types are ranked in descending order of world incidence (see also Fig. 2). Only tumors whose use is described by three or more independent studies are shown. Arrows at the beginning of each row of the table symbolize deviation of the frequency of tumor targeted in experimental cytotherapy work from their respective incidence/frequency of occurrence globally (yellow = difference within 5%; green, up = difference > 5% in favor of cytotherapy—tumor over-representation; red, down = difference of > 5% in favor of incidence—tumor under-representation). */**/# Studies referring to cervical cancer/ ovarian cancer/ use of UC-blood MSC, respectively. (TIF 9450 kb) [file 13287_2018_1078_MOESM6_ESM.tif]
